# Nucleophilic fluorine substitution reaction of α-carbonyl benzyl bromide, phenylthiofluoroalkyl bromide, and 2-bromo-2-phenoxyacetonitrile†

**DOI:** 10.1039/d4ra03085k

**Published:** 2024-06-13

**Authors:** Satoshi Mizuta, Tomoko Yamaguchi, Takeshi Ishikawa

**Affiliations:** a Center for Bioinformatics and Molecular Medicine, Graduate School of Biomedical Sciences, Nagasaki University 1-14 Bunkyo Nagasaki 852-8521 Japan; b Department of Chemistry, Biotechnology, and Chemical Engineering, Graduate School of Science and Engineering, Kagoshima University 1-21-40 Korimoto Kagoshima 890-0065 Japan

## Abstract

We herein describe a new method for nucleophilic fluorine substitution of alkylbromides using Et_3_N·3HF. The process is characterized by a broad substrate scope, good functional-group compatibility, and mild conditions and provides a variety of alkylfluorides including tertiary alkylfluorides that are versatile and structurally attractive.

## Abstract 2

This manuscript discloses the nucleophilic fluorination of various alkylbromides using EtN_3_·3HF, producing monofluorinated compounds including tertiary benzylfluoride.

## Graphical abstract

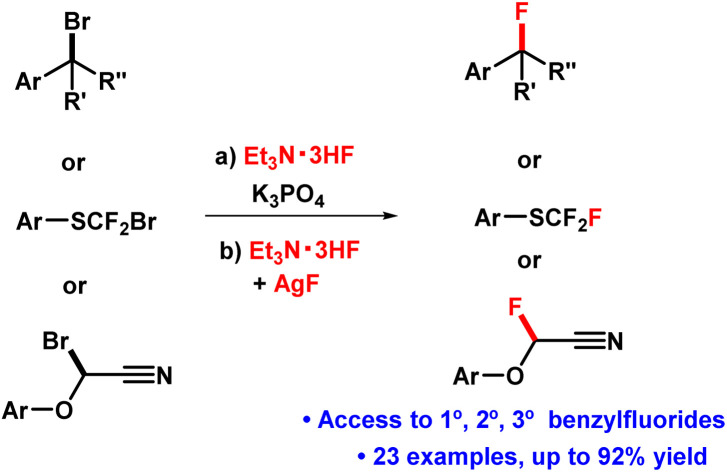

The development of methods for C(sp^3^)–F bond formation has been in great demand in medicinal and biological chemistry because organofluorine compounds are attractive structural motifs as agrochemicals, pharmaceuticals, and ^18^F-labeled radiotracers for positron emission tomography imaging.^1–4^ The nucleophilic substitution reaction involving S_N_1 and S_N_2 reactions is one of the most basic and substantial transformations in organic chemistry. Traditionally, various methods for preparing alkylfluorides from alkyl halides or sulfonates using nucleophilic fluorination reagents such as silver fluoride (AgF), potassium fluoride (KF), cesium fluoride (CsF), tetrabutyl ammonium fluoride (TBAF), anhydrous HF, and amine/HF reagents [*e.g.* triethylamine tris(hydrogen fluoride) (Et_3_N·3HF) and Olah's reagent (pyridine·9HF)] have been developed extensively.^5–8^ Among the most applied was the halogen exchange fluorination with metal fluoride in an S_N_2 reaction that is generally amenable to primary and secondary alkyl halides including benzylic halides, α-halo ketones, and related electrophiles. Meanwhile, for alkyl C(sp^3^)–F bond formation, C–H fluorination,^9^ hydrofluorination,^10^ and decarboxylative fluorination^11^ using nucleophilic fluorine sources have been developed. Despite these advances, only a few examples have been reported on nucleophilic substitution reactions of alkylhalide access to alkylfluorides including tertiary alkylfluorides which limit its potential reaction window.^12–14^

Although it is generally preferable to use neutral nucleophiles for the S_N_1 reaction, the highly toxic HF gas causes undesirable reactions due to its acidity. In 1980, Franz prepared weakly corrosive Et_3_N·3HF which is a colorless liquid with its pH close to neutral.^15^ Since that time, Et_3_N·3HF as a fluoride source for nucleophilic monofluorination in organic molecules has been used frequently.^16^ In particular, Et_3_N·3HF has frequently been used in electrochemical fluorination of thioethers and *O*,*S*-acetal derivatives bearing α-electron-withdrawing groups in the last decades.^6,17^ However, due to its weak nucleophilicity, the fluorination reactions of alkylhalides with Et_3_N·3HF usually require harsh reaction conditions, particularly high temperatures. Thus, it is of interest to find mild conditions and a class of alkylbromides forming carbocation intermediates that allow fluorine-bromine exchanges.

Recently, Doyle and co-workers developed a method for nucleophilic fluorination of *N*-hydroxyphthalimide esters using Et_3_N·3HF as a fluorine source *via* the carbocation generation under photocatalysis (Fig. 1A).^18^ Recent advances in this area: the direct replacement of bromide or chloride at tertiary carbon centers using AgF under phosphine catalysis have been reported by Fu (Fig. 1B).^19^ From prior arts, we envisaged that Et_3_N·3HF and AgF reagents would be potential for employing as fluorine sources in the halogen exchange of alkylhalides *via* the S_N_1 process. In this paper, we describe the development of methods for the nucleophilic fluorine substitution to α-carbonyl benzyl bromides, phenylthiofluoroalkyl bromides, and 2-bromo-2-phenoxyacetonitriles with Et_3_N·3HF, leading to the desired alkylfluorides (Fig. 1C). Notably, a combination of AgF and Et_3_N·3HF enhanced the reactivity compared with the single use of AgF, exhibiting an increase yield for monofluorinated products.

**Fig. 1:**
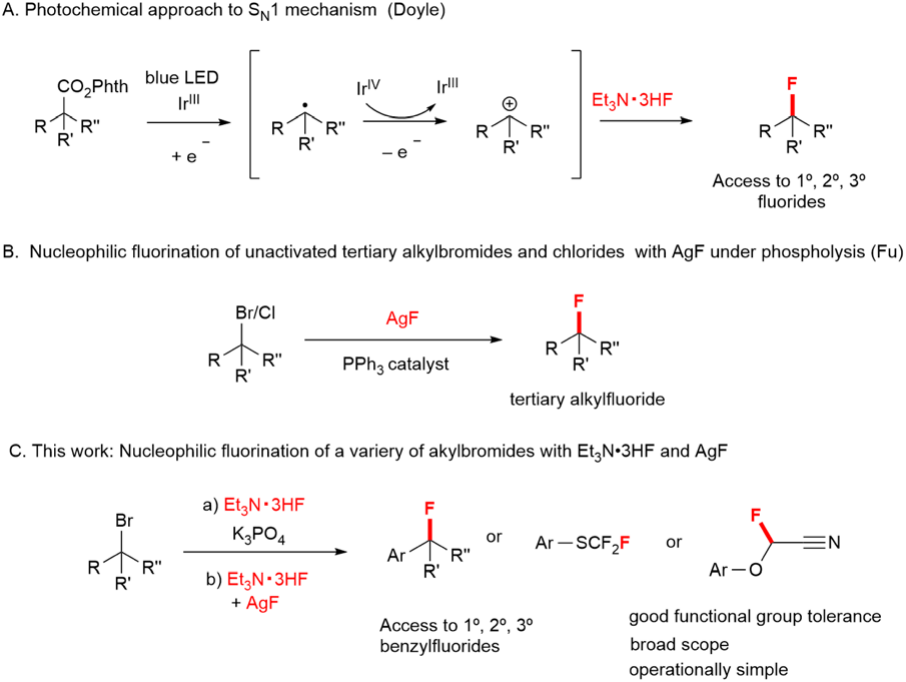
Nucleophilic substitution reactions with nucleophilic fluorinating reagents.

Our initial efforts focused on the halogen exchange reactions of α-bromo phenylacetate (1a) as a model substrate in a variety of fluorine sources for optimal conditions (Table 1). The reaction of the substrate 1a using 8.0 equivalent of Et_3_N·3HF in ethylene glycol dimethyl ether (DME) at 80 °C obtained the desired fluorinated product 2a in 18% yield (entry 1). The yield of 2a was increased by the addition of K_3_PO_4_ as the base up to 62% (entry 2). Then, it was found that solvent significantly affects the reactivity of fluorination (entries 3–5): acetonitrile was superior to other solvents, giving the product 2a in 68% isolated yield. Further screening of the nucleophilic fluorinating reagents such as Olar's reagent, KF, and CsF (entries 6–9), in which use of them provided a loss yield. In contrast, AgF as a fluorine source successfully employed the nucleophilic fluorination of benzylbromide 1a without a base at room temperature under mild conditions, providing the desired fluorinated product 2a in 40% yield, regardless of the heterogeneous reaction (entry 9). Interestingly, the addition of Et_3_N·3HF aids in dissolving AgF salt in acetonitrile. Then, the combination of Et_3_N·3HF and AgF as fluorine sources improved the fluorine–bromine exchange, furnishing the product with high yield (entry 10).

**Table 1:** Optimization of conditions for the nucleophilic fluorination of 1a

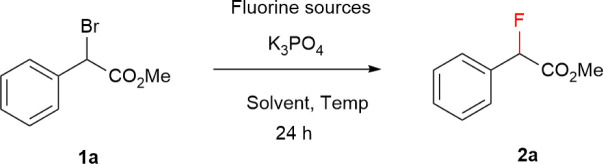					
Entry	Fluorine sources (equiv.)	K_3_PO_4_ (equiv.)	Solvent	Temp. (°C)	Yield^a^ (%)
1	Et_3_N·3HF (8)	None	DME	80	18
2	Et_3_N·3HF (8)	1.2	DME	80	62
3	Et_3_N·3HF (8)	1.2	THF	80	63
4	Et_3_N·3HF (8)	1.2	MeCN	80	78 (68)^b^
5	Et_3_N·3HF (8)	1.2	DMF	80	38
6	Py·HF (8)	1.2	MeCN	80	0
7	KF (8)	1.2	MeCN	80	0
8	CsF (8)	1.2	MeCN	80	30
9	AgF (2)	None	MeCN	r.t.	40
10	AgF (2), Et_3_N·3HF (3)	None	MeCN	r.t.	83 (74)^b^

a Yields determined by ^19^F NMR spectroscopy using fluorobenzene as an internal standard.

b Isolated yield in parentheses.

The fluorination reactions of benzylbromides 1b–t were carried out using 8 equiv. of Et_3_N·3HF, and 1.2 equiv. of K_3_PO_4_ in MeCN at 80 °C (Method A). On the other hand, as in Method B: reaction conditions with benzyl bromides 1b–t, a combination of AgF (2.0 equiv.) and Et_3_N·3HF (3.0 equiv.) in MeCN at room temperature was also used. With these optimized conditions in hand, we examined the scope and limitations of this method for the fluorine substitution of alkyl halides. As shown in Fig. 2, α-bromo benzylacetates 1b–j bearing substituents including halogen and electron-withdrawing group and electron-donating group underwent fluorine–bromine exchange to afford the desired products 2b–h in satisfactory yields as well as tertiary benzylic fluorides (2i, and 2j). In a gram scale reaction of methyl 2-bromo 2-(2-methoxyphenyl)acetate 1g, the fluorinated product 2g could be obtained in a higher yield (92%) than that of a 0.15 mmol scale reaction (76%). Furthermore, we found that DME was a suitable solvent for the fluorination reactions of α-bromo benzylacetic acid under reaction conditions of Method A (see, Table S1†). A class of α-bromo benzylacetic acid substrates 1k–m was subject to the fluorination reaction followed by esterification to isolate the corresponding methyl ester. When Method B was used, the substitution reaction of α-bromo benzylamide 1n afforded alkylfluoride 2n in 74% yield. The primary and secondary benzylfluorides 2o–q were obtained with moderate yield under the optimization conditions. Still, this new method is not applied to substrates such as α-chloro benzylacetate (1r), trityl bromide (1s), and α-bromo phenyl propanoate (1t). For 1r and 1s, their poor reactivity resulted in the recovered starting material, and for 1t the elimination reaction occurred rapidly.

**Fig. 2:**
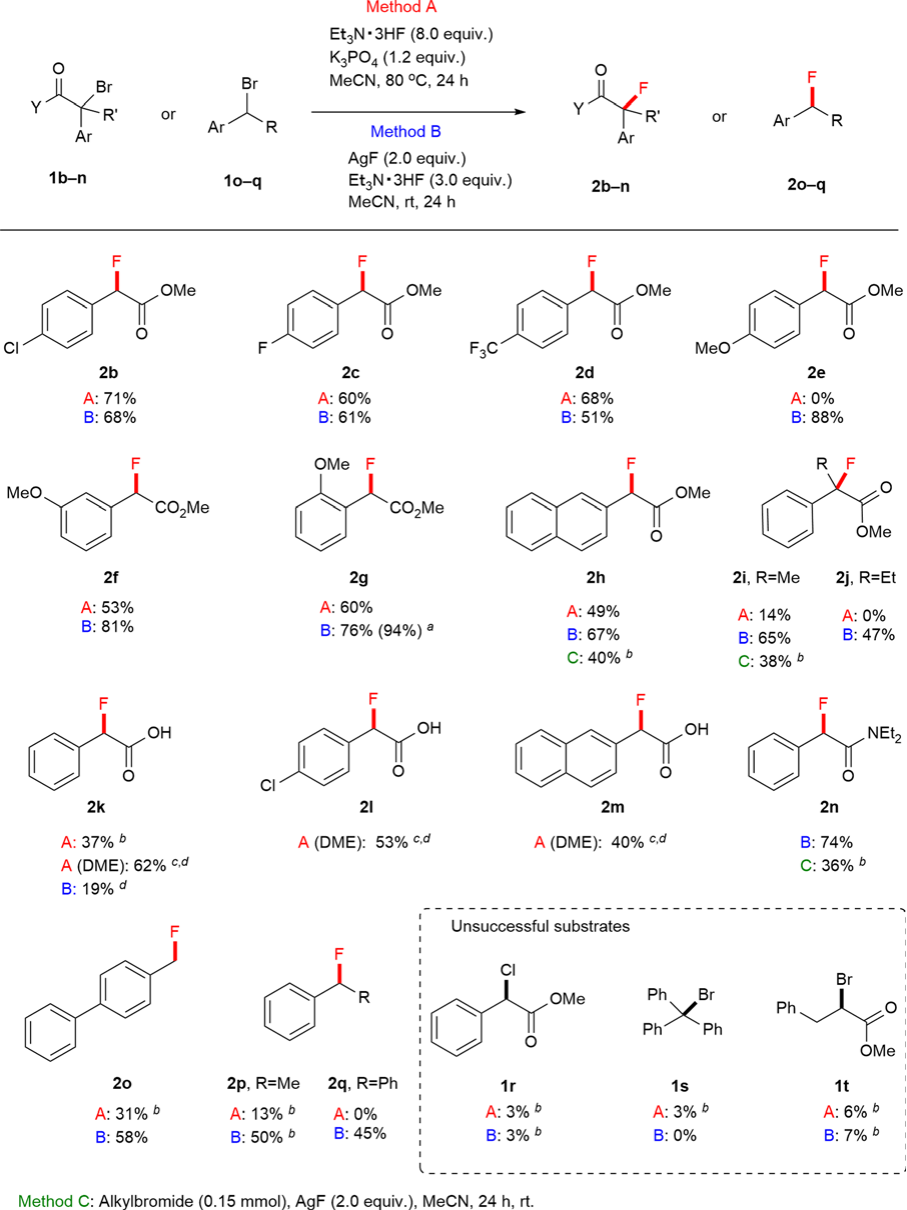
Scope of benzylic fluorination (0.15 mmol scale). ^*a*^Performed on a 4.0 mmol scale, affording 748 mg of product 2g (94% isolated yield). ^*b*19^F NMR yields was determined using fluorobenzene as an internal standard. ^*c*^DME was used as a solvent. ^*d*^Yield of isolated methyl ester with esterification using H_2_SO_4_ as a catalyst in MeOH.

The thio carbenium and oxocarbenium ions can be stabilized by the interaction between the lone-pair electron of sulfur and oxygen atom with the unoccupied p-orbital of the carbocation, those reported in the literature.^20^ The phenylthiofluoroalkyl bromide, and 2-bromo-2-phenoxyacetonitrile are precursors to form thio carbenium and oxocarbenium ions, respectively. We demonstrated the introduction of fluorine to phenylthiofluoroalkyl bromide 3a–c, and 2-bromo-2-phenoxyacetonitrile 4a–c bearing neighbouring groups such as fluorine, sulfur, and oxygen atoms, as illustrated in Fig. 3. When Method B was used, the substitution reaction of phenylthiofluoroalkyl bromide 3a–c provided a good yield of aryl trifluoromethyl sulfide to be a biologically effective functional group.^21^ Additionally, 2-fluoro-2-phenoxyacetonitrile compounds 6a–c were readily obtained from alkylbromide substrates 4a–c in better yields *via* fluorine–bromine exchange when both Method A and B were used, compared to when the substitution using AgF as a fluorine source.

**Fig. 3:**
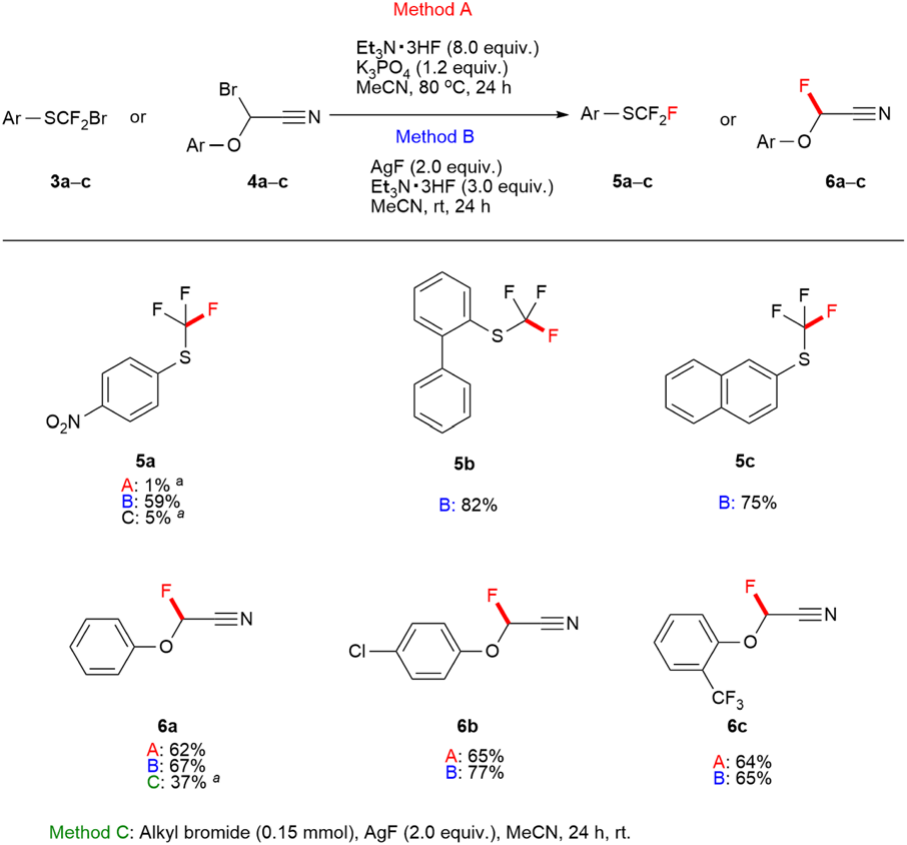
Scope of phenylthiofluoroalkyl bromides, and 2-bromo-2-phenoxyacetonitriles (0.15 mmol scale). ^*a*19^F NMR yields were determined using fluorobenzene as an internal standard.

To gain insight into the reaction mechanism, a stereochemical study was conducted. At first, the substitution reactions of α-racemic α-bromo phenyllactate (7) afforded the corresponding product 8 without stereoselectivity under reaction conditions of Methods A and B. Subsequently, we synthesized a highly diastereoenriched (αR)-α-bromo phenylacetate (9) though crystallization induced dynamic resolution (CIDR) following the previous work reported by Park.^22^ The fluorine substitution of (αR)-α-bromo arylacetate (9) *via* the S_N_1 process should occur epimerization of the α-carbon because it forms a carbocation intermediate. Virtually, the substitution reaction of (αR)-9 under standard conditions of Method A observed the epimerization in the fluorinated product (10). In contrast, the stereochemistry in the reaction with AgF showed the predominate inversion with dr of 92 : 8 and 59% yield. The substitution reaction in the presence of Et_3_N·3HF and AgF revealed the stereochemistry with dr of 83 : 17 and a high yield of 92%. These results indicate that two pathways including S_N_1 and S_N_2 processes exist in fluorination reactions of Method B (Scheme 1).

**Scheme 1:**
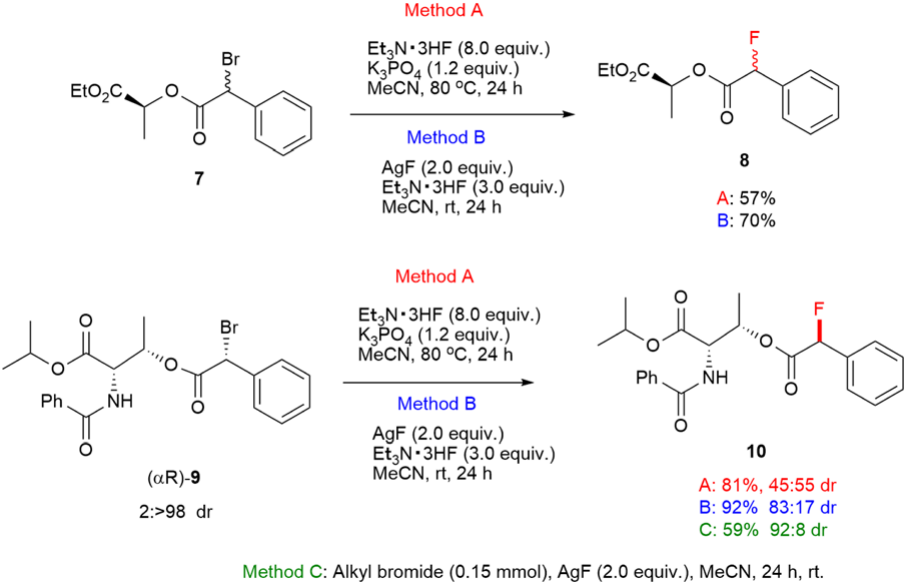
Asymmetric fluorination with α-bromo phenyllactate 7 and (αR)-α-bromo phenylacetate (αR)-9.

To investigate the stability of Et_3_N·3HF under standard conditions, ^19^F NMR studies were performed. Et_3_N·3HF complex consists of N–H⋯F hydrogen bond and the central fluoride coordinated with an H_2_F_3_^−^ or [F(HF)_2_]^−^ ion according to an equilibrium.^23^ However, Et_3_N·3HF in acetonitrile converts to Et_3_N·2HF, Et_3_N·HF, and HF after 12 h at 80 °C. This was confirmed by ^19^F NMR spectroscopy, of which spectra showed that the chemical shift of Et_3_N·3HF (−166 ppm) completely disappeared, and three additional signals appeared at −127, −151, and −152 ppm as same as those reported in the literature (Fig. S2†).^24^ Further studies found that the presence of K_3_PO_4_ decreased the signal for acidic HF. Previously reported literature indicates the nucleophilicity varies in the order Et_3_N·2HF > Et_3_N·3HF > Et_3_N·HF.^25^ Therefore, *in situ* generated Et_3_N·2HF may act as a part of active species for fluorine substitution to putative carbocation intermediate when Method A is used. Whereas, Et_3_N·3HF assists in solubilizing AgF in acetonitrile and the resulting solution seems to stabilize Et_3_N·3HF after 16 h at room temperature. In the obtained ^19^F NMR spectra, the most was a signal of Et_3_N·3HF (162 ppm) and weak signals of HF and HF_2_^−^ (−128 and −145 ppm) were observed without signals of Et_3_N·2HF, Et_3_N·HF (Fig. S3†). Based on these results, the reaction pathway of Method A might proceed through the generation of a carbocation intermediate from α-bromo phenylacetate (1a) followed by the nucleophilic attack of *in situ* generated Et_3_N·2HF, Et_3_N·HF, or HF (Fig. 4a). Concerning the mechanism for Method B, we speculate that while the S_N_2 reaction pathway mediated by AgF is proceeding, the S_N_1 type reaction is also proceeding through Ag-assisted dissociation of bromide to form carbocation intermediate followed by the fluorine substitution with Et_3_N·3HF (Fig. 4b).

**Fig. 4:**
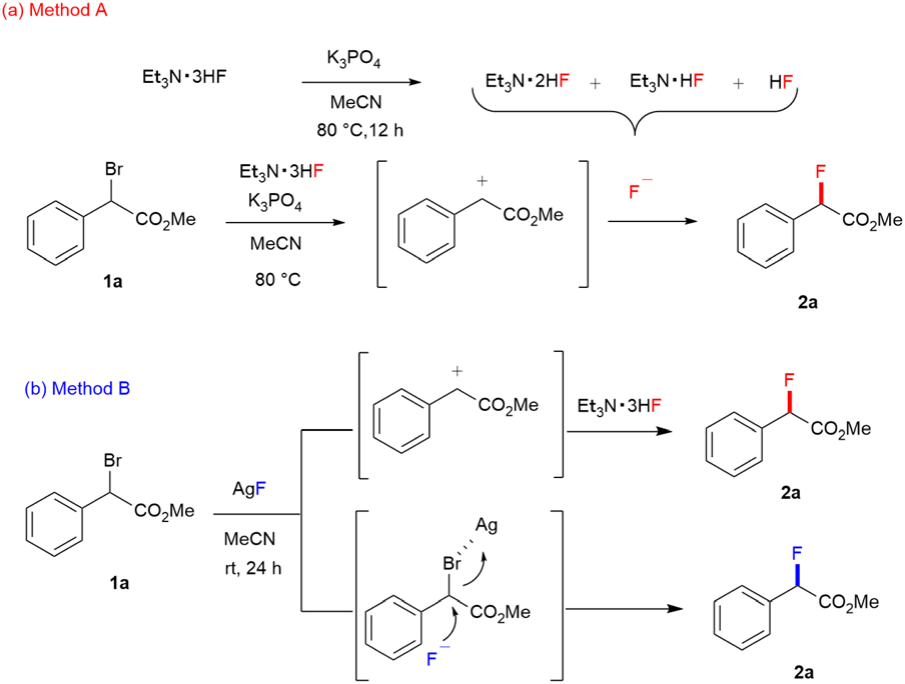
(a) The plausible reaction pathway of Method A with Et_3_N·3HF in the presence of K_3_PO_4_. (b) The plausible reaction pathway of Method B using a combination of Et_3_N·3HF and AgF.

## Conclusions

We demonstrated that Et_3_N·3HF is an efficient reagent to accomplish bromine–fluorine exchange on α-carbonyl benzyl bromides, phenylthiofluoroalkyl bromides, and 2-bromo-2-phenoxyacetonitriles. One strategy for the fluorine substitution with Et_3_N·3HF in the presence of K_3_PO_4_ has been developed. Another strategy for fluorine substitution using a combination of Et_3_N·3HF and AgF has also been developed. These methods showed good advantages such as a broad scope of substrates involving tertiary alkylbromides, functional group tolerance, mild conditions, and operationally simple. Further investigation on the ability of Et_3_N·3HF for the nucleophilic fluorination reactions is undergoing.

## Author contributions

The synthesis of alkylfluorides was carried out by SM and TY. The characterization and data curation were made by SM and TI. SM and TI were responsible for financial resource. The manuscript was written through contributions of ESI.† All authors have given approval to the final version of the manuscript.

## Conflicts of interest

There is no conflict of interest to declare.

## Supplementary Material

RA-014-D4RA03085K-s001
