# Supplementary material for: Nucleophilic fluorine substitution reaction of α-carbonyl benzyl bromide, phenylthiofluoroalkyl bromide, and 2-bromo-2-phenoxyacetonitrile
Source: RSC Adv. 2024 Jun 13;14(27):19062–6. doi: 10.1039/d4ra03085k (PMC11172408; doi:10.1039/d4ra03085k)
Supplement: RA-014-D4RA03085K-s001 [file RA-014-D4RA03085K-s001.pdf]

Electronic Supplementary Material (ESI) for RSC Advances.
This journal is © The Royal Society of Chemistry 2024

Nucleophilic fluorine substitution reaction of *α*-carbonyl benzyl bromide, phenylthiofluoroalkyl bromide, and 2-bromo-2-phenoxyacetnitirle

Satoshi Mizuta,[a]* Tomoko Yamaguchi,[a] and Takeshi Ishikawa[b]

[a] Center for Bioinformatics and Molecular Medicine, Graduate School of Biomedical Sciences, Nagasaki University, 1-14 Bunkyo, Nagasaki, 852-8521, Japan
[b] Department of Chemistry, Biotechnology, and Chemical Engineering, Graduate School of Science and Engineering, Kagoshima University, 1-21-40 Korimoto, Kagoshima 890-0065, Japan

E-mail S.M. s-mizuta@nagasaki-u.ac.jp

Table of contents

1. **General Information....................S2**
2. **Select Optimization Results for α-Bromo Phenylacetic acid (1k) S3**
3. **Experimental Procedures and Characterization Data....................S4−S16**
4. **Investigation on The Stability of $Et_3N·3HF$....................S17−S18**
5. **References....................S18**
6. **NMR Spectra....................S19−S51**

1. General Information

All nuclear magnetic resonance (NMR) spectra were recorded on a Varian 500PS spectrometer, or on JEOL JMN-ECZ400R. ^{1}H, ^{13}C and ^{19}F NMR spectra were reported as chemical shifts (δ) in parts per million (ppm) relative to the solvent peak using tetramethylsilane (^{1}H and ^{13}C) as an internal standard. Chemical shifts (δ) are quoted in parts per million (ppm), and coupling constants (*J*) are measured in hertz (Hz). The following abbreviations were used to describe multiplicities: s = singlet, d = doublet, t = triplet, q = quartet, quint. = quintet, sext. = sextet, sept. = septet, br = broad, m = multiplet. The NMR spectra were processed using ACD/SpecManager. High-resolution mass spectra (HRMS, *m*/*z*) were obtained on a JEOL JMS-700N for fast atom bombardment (FAB) using m-nitrobenzyl alcohol as a matrix or electron ionization (EI). All the reactions were performed in an apparatus with magnetic stirring under an inert atmosphere. Flash column chromatography was performed using Fuji Silysia Chemical Ltd. Silica Gel C60 (50–200 μm) using an eluent system, as described in the next section, i.e., Experimental Procedures. Thin-layer chromatography was performed using TLC Silica Gel 60 F_{254} aluminum sheets (Merck) and Silica Gel F_{254} glass plates (Merck).

Materials. Unless otherwise stated, all starting materials and reagents were obtained from commercial suppliers and were used without further purification. Chemicals were purchased from Aldrich, Nacalai Tesque, Tokyo Chemical Industry, and Wako Pure Chemical Industries and used as received. All solvents were purchased from Wako Pure Chemical Industries.

Safety and Hazards. According to general procedures A and B as described below, a part of $Et_3N/3HF$ converts to hydrogen fluoride. Thus, the solution involving $Et_3N/3HF$ has to be handled carefully and any contact with the skin must be avoided.

2. Select Optimization Results for α-Bromo Phenylacetic acid (1k)

Table S1. Optimization of conditions for the nucleophilic fluorination of α-bromo benzylacetic acid (**1k**)

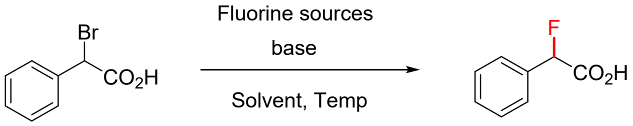

Entry	Fluorine sources (equiv)	Base (equiv)	Solvent	Temp (°C)	Time (h)	Yield[a] (%)
1	$Et_3N{\cdot}3HF$ (3.6)	K_3PO_4 (1.2)	THF	rt	24	11
2	$Et_3N{\cdot}3HF$ (3.6)	K_3PO_4 (1.2)	THF	80	12	31
3	Py·9HF (3.6)	K_3PO_4 (1.2)	THF	80	12	0
4	$Et_3N{\cdot}3HF$ (3.6)	K_3PO_4 (1.2)	MeCN	80	12	22
5	$Et_3N{\cdot}3HF$ (3.6)	K_3PO_4 (1.2)	DMF	80	12	3
6	$Et_3N{\cdot}3HF$ (3.6)	K_3PO_4 (1.2)	DCE	80	12	6
7	$Et_3N{\cdot}3HF$ (3.6)	K_3PO_4 (1.2)	Toluene	80	12	29
8	$Et_3N{\cdot}3HF$ (3.6)	K_3PO_4 (1.2)	1,4-dioxane	80	12	40
9	$Et_3N{\cdot}3HF$ (3.6)	K_3PO_4 (1.2)	DME	80	12	42
10	$Et_3N{\cdot}3HF$ (3.6)	K_3PO_4 (2.0)	DME	80	12	28
11	$Et_3N{\cdot}3HF$ (3.6)	none	DME	80	12	19
12	$Et_3N{\cdot}3HF$ (3.6)	iPr_2NEt (1.2)	DME	80	12	44
13	$Et_3N{\cdot}3HF$ (3.6)	DBU (1.2)	DME	80	12	38
14	$Et_3N{\cdot}3HF$ (3.6)	*t*BuOK (1.2)	DME	80	12	29
15	$Et_3N{\cdot}3HF$ (8.0)	K_3PO_4 (1.2)	MeCN	80	24	37
16	$Et_3N{\cdot}3HF$ (8.0)	K_3PO_4 (1.2)	DME	80	24	62 (62)[b]

[a] Yields determined by ^{19}F NMR spectroscopy using fluorobenzene as an internal standard. [b] Yield of isolated methyl ester with esterification using H_2SO_4 as a catalyst in MeOH.

3. Experimental Procedures and Characterization Data

General procedure A for the synthesis of alkylfluorides

A 15 mL test tube quipped with a magnetic stirring bar was charged with alkylbromide (0. 15 mmol), K_3PO_4 (1.2 equiv), $Et_3N\cdot3HF$ (8.0 equiv) and MeCN (0.15 M). The resulting mixture was stirred at 80 °C for 24 h. The reaction was quenched with saturated aqueous NH_4Cl after cooling to room temperature; the crude mixture was extracted with AcOEt. The combined organic layers were washed with brine, dried over $MgSO_4$ and evaporated in vacuo. The residue was purified by column chromatography on SiO_2 gel and eluted with hexane-AcOEt, affording the alkylfluoride .

Note: This reaction system can be used in ordinary borosilicate glassware, but after some experiments it seems to be corrosive on the glass face.

General procedure B for the synthesis of alkylfluorides

A 15 mL test tube quipped with a magnetic stirring bar was charged with alkylbromide (0.15 mmol), AgF (2.0 equiv), $Et_3N\cdot3HF$ (3.0 equiv) and MeCN (0.15 M). The resulting mixture was stirred at room temperature for 24 h. For the precipitate removal, the mixture was filtered through a Celite pad and washed with AcOEt. To the crude mixture added saturated aqueous NH_4Cl, the combined organic layers were washed with brine, dried over $MgSO_4$ and evaporated in vacuo. The residue was purified by column chromatography on SiO_2 gel and eluted with hexane-AcOEt, affording the alkylfluoride .

General procedure C for the synthesis of alkylfluorides

A 15 mL test tube quipped with a magnetic stirring bar was charged with alkylbromide (0.15 mmol), AgF (2.0 equiv), and MeCN (0.15 M). The resulting mixture was stirred at room temperature for 24 h. For the precipitate removal, the mixture was filtered through a Celite pad and washed with AcOEt. To the crude mixture added saturated aqueous NH_4Cl, the combined organic layers were washed with brine, dried over $MgSO_4$ and evaporated in vacuo. The residue was purified by column chromatography on SiO_2 gel and eluted with hexane-AcOEt, affording the alkylfluoride .

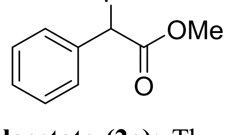

Synthesis of methyl 2-fluoro-2-phenylacetate (2a): The general procedure A was followed with methyl 2-bromo-2-phenylacetate (34.3 mg, 0.15 mmol), K_3PO_4 (38.2 mg, 0.18 mmol), $Et_3N\cdot3HF$ (202 μL, 1.2 mmol) and MeCN (1.0 mL). The crude was purified by column chromatography and eluted with hexane−AcOEt (4:1). This afforded the title compound as a colorless oil (17.2 mg, 68%

yield). ^{1}H NMR (500 MHz, $CDCl_3$): δ 7.51−7.45 (m, 2H), 7.43−7.42 (m, 3H), 5.81 (d, J = 47.4 Hz, 1H), 3.80 (s, 3H); $^{13}C\{^1H\}$ NMR (125 MHz, $CDCl_3$): δ 169.9 (d, J = 27.5 Hz), 134.1 (d, J = 19.9 Hz), 129.6 (d, J = 18.9 Hz), 128.8, 126.6 (d, J = 5.7 Hz), 89.3 (d, J = 184.7 Hz), 52.6; ^{19}F NMR (470 MHz, $CDCl_3$): −179.9 (dd, J =4.4, 46.9 Hz, 1F); HRMS (EI) *m/z* Calcd for $C_9H_9FO_2$ $[M]^+$ 168.0586 found 168.0587.

The general procedure B was followed with methyl 2-bromo-2-phenylacetate (34.3 mg, 0. 15 mmol), AgF (38.1 mg, 0.30 mmol), $Et_3N{\cdot}3HF$ (74 μL, 0.45 mmol) and MeCN (1.0 mL), giving the title compound (18.7 mg, 74% yield).

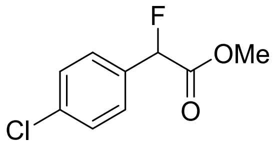

Synthesis of methyl 2-(4-chlorophenyl)-2-fluoroacetate (2b): The general procedure A was followed with methyl 2-bromo-2-(4-chlorophenyl)acetate (39.5 mg, 0.15 mmol), K_3PO_4 (38.2 mg, 0.18 mmol), $Et_3N{\cdot}3HF$ (202 μL, 1.2 mmol) and MeCN (1.0 mL). The crude was purified by column chromatography and eluted with hexane−AcOEt (8:1). This afforded the title compound as a colorless oil (21.6 mg, 71% yield). ^{1}H NMR (500 MHz, $CDCl_3$): δ 7.42−7.39 (m, 4H), 5.78 (d, J = 47.2 Hz, 1H), 3.79 (s, 3H); $^{13}C\{^1H\}$ NMR (125 MHz, $CDCl_3$): δ 168.6 (d, J = 27.5 Hz), 135.7 (d, J = 2.8 Hz), 132.6 (d, J = 19.9 Hz), 129.1, 127.9 (d, J = 6.6 Hz), 88.6 (d, J = 185.7 Hz), 52.8; ^{19}F NMR (470 MHz, $CDCl_3$): −179.9 (dd, J =4.3, 46.9 Hz, 1F); HRMS (EI) *m/z* Calcd for $C_9H_8ClFO_2$ $[M]^+$ 202.0197 found 202.0197.

The general procedure B was followed with methyl 2-bromo-2-(4-chlorophenyl)acetate (39.5 mg, 0. 15 mmol), AgF (38.1 mg, 0.30 mmol), $Et_3N{\cdot}3HF$ (74 μL, 0.45 mmol) and MeCN (1.0 mL), giving the title compound (18.7 mg, 61% yield).

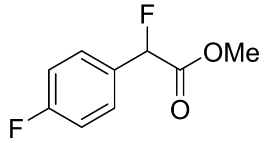

Synthesis of methyl 2-(4-fluorophenyl)-2-fluoroacetate (2c): The general procedure A was followed with methyl 2-bromo-2-(4-fluorophenyl)acetate (37.0 mg, 0.15 mmol), K_3PO_4 (38.2 mg, 0.18 mmol), $Et_3N{\cdot}3HF$ (202 μL, 1.2 mmol) and MeCN (1.0 mL). The crude was purified by column chromatography and eluted with hexane−AcOEt (10:1). This afforded the title compound as a colorless oil (16.8 mg, 60% yield). ^{1}H NMR (400 MHz, $CDCl_3$): δ 7.48−7.44 (m, 2H), 7.11 (t, J = 9.2 Hz, 2H), 5.78 (d, J = 47.4 Hz, 1H), 3.80 (s, 3H); $^{13}C\{^1H\}$ NMR (100 MHz, $CDCl_3$): δ 168.8 (d, J = 28.8 Hz), 163.5 (d, J = 248.2 Hz), 130.1 (d, J = 21.1 Hz), 128.8 (d, J = 8.6 Hz), 128.7 (d, J = 8.6 Hz), 88.7 (d, J = 185.0 Hz), 52.7; ^{19}F NMR (376 MHz, $CDCl_3$): −111.1− −111.2 (m, 1F), −178.5 (dd,

J = 2.4, 46.8 Hz, 1F); HRMS (EI) *m/z* Calcd for $C_9H_8F_2O_2$ $[M]^+$ 186.0492 found 186.0491.

The general procedure B was followed with methyl 2-bromo-2-(4-chlorophenyl)acetate (37.0 mg, 0. 15 mmol), AgF (38.1 mg, 0.30 mmol), $Et_3N{\cdot}3HF$ (74 µL, 0.45 mmol) and MeCN (1.0 mL), giving the title compound (17.0 mg, 61% yield).

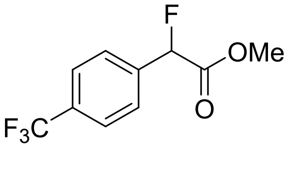

Synthesis of methyl 2-fluoro-2-(4-(trifluoromethyl)phenyl)acetate (2d): The general procedure A was followed with methyl 2-bromo-2-(4-(trifluoromethyl)phenyl)acetate (44.6 mg, 0.15 mmol), K_3PO_4 (38.2 mg, 0.18 mmol), $Et_3N{\cdot}3HF$ (202 µL, 1.2 mmol) and MeCN (1.0 mL). The crude was purified by column chromatography and eluted with hexane−AcOEt (9:1). This afforded the title compound as a colorless oil (23.9 mg, 68% yield). ^{1}H NMR (500 MHz, $CDCl_3$): δ 7.69 (d, J = 8.4 Hz, 2H), 7.61 (d, J = 8.3 Hz, 2H), 5.88 (d, J = 47.4 Hz, 1H), 3.81 (s, 3H); $^{13}C\{^1H\}$ NMR (125 MHz, $CDCl_3$): δ 168.2 (d, J = 27.5 Hz), 137.8 (d, J = 19.9 Hz), 131.7 (q, J = 32.2 Hz), 126.6 (d, J = 6.6 Hz), 125.8 (q, J = 3.8 Hz), 123.7 (q, J = 270.9 Hz), 88.5 (d, J = 186.6 Hz), 52.9; ^{19}F NMR (376 MHz, $CDCl_3$): −62.7 (s, 3F), −184.2 (d, J = 47.6 Hz, 1F); HRMS (EI) *m/z* Calcd for $C_{10}H_8F_4O_2$ $[M]^+$ 236.0460 found 236.0468.

The general procedure B was followed with methyl 2-bromo-2-(4-(trifluoromethyl)phenyl)acetate (44.6 mg, 0.15 mmol), AgF (38.1 mg, 0.30 mmol), $Et_3N{\cdot}3HF$ (74 µL, 0.45 mmol) and MeCN (1.0 mL), giving the title compound (18.1 mg, 51% yield).

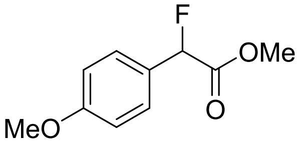

Synthesis of methyl 2-fluoro-2-(4-methoxyphenyl)acetate (2e): The general procedure B was followed with methyl 2-bromo-2-(4-methoxyphenyl)acetate (38.8 mg, 0. 15 mmol), AgF (38.1 mg, 0.30 mmol), $Et_3N{\cdot}3HF$ (74 µL, 0.45 mmol) and MeCN (1.0 mL). The crude was purified by column chromatography and eluted with hexane−AcOEt (9:1). This afforded the title compound as a colorless oil (26.3 mg, 88% yield). ^{1}H NMR (400 MHz, $CDCl_3$): δ 7.39 (dd, J = 1.6, 8.5 Hz, 2H), 6.93 (dd, J = 0.7, 8.9 Hz, 2H), 5.74 (d, J = 47.6 Hz, 1H), 3.83 (s, 3H), 3.79 (s, 3H); $^{13}C\{^1H\}$ NMR (100 MHz, $CDCl_3$): δ 169.3 (d, J = 28.7 Hz), 160.7, 128.6, 128.5, 126.2 (d, J = 21.1 Hz), 114.2, 89.1 (d, J = 184.0 Hz), 55.3, 52.5; ^{19}F NMR (470 MHz, $CDCl_3$): −174.6 (d, J = 47.6 Hz, 1F); HRMS (EI) *m/z* Calcd for $C_{10}H_{11}FO_3$ $[M]^+$ 198.0692 found 198.0692.

Note: Methyl 2-bromo-2-(4-methoxyphenyl)acetate (**1e**) decomposed spontaneously due to its stability after it left at room temperature for a few days.

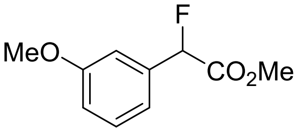

Synthesis of methyl 2-fluoro-2-(3-methoxyphenyl)acetate (2f): The general procedure A was followed with methyl 2-bromo-2-(2-methoxyphenyl)acetate (38.8 mg, 0.15 mmol), K_3PO_4 (38.2 mg, 0.18 mmol), $Et_3N \cdot 3HF$ (202 μL, 1.2 mmol) and MeCN (1.0 mL). The crude was purified by column chromatography and eluted with hexane−AcOEt (10:1). This afforded the title compound as a colorless oil (15.8 mg, 53% yield). ^1H NMR (400 MHz, $CDCl_3$): δ 7.33 (t, J = 8.0 Hz, 1H), 7.04 (d, J = 7.6 Hz, 1H), 2.00 (s, 1H), 6.96−6.93 (m, 1H), 5.77 (d, J = 47.6 Hz, 1H), 3.83 (s, 3H), 3.79 (s, 3H); $^{13}C\{^1H\}$ NMR (100 MHz, $CDCl_3$): δ 168.9 (d, J = 27.8 Hz), 159.8, 135.4 (d, J = 21.0 Hz), 129.9, 118.9 (d, J = 6.7 Hz), 115.4 (d, J = 2.9 Hz), 111.8 (d, J = 6.7 Hz), 89.2 (d, J = 185.0 Hz), 55.3, 52.7; ^{19}F NMR (376 MHz, $CDCl_3$): −180.2 (d, J = 47.7 Hz, 1F); HRMS (EI) *m/z* Calcd for $C_{10}H_{11}FO_3$ $[M]^+$ 198.0692 found 198.0692.

The general procedure B was followed with methyl 2-bromo-2-(3-methoxyphenyl)acetate (38.8 mg, 0. 15 mmol), AgF (38.1 mg, 0.30 mmol), $Et_3N \cdot 3HF$ (74 μL, 0.45 mmol) and MeCN (1.0 mL), giving the title compound (24.2 mg, 81% yield).

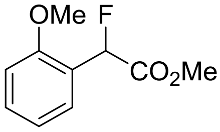

Synthesis of methyl 2-fluoro-2-(2-methoxyphenyl)acetate (2g): The general procedure A was followed with methyl 2-bromo-2-(2-methoxyphenyl)acetate (38.8 mg, 0.15 mmol), K_3PO_4 (38.2 mg, 0.18 mmol), $Et_3N \cdot 3HF$ (202 μL, 1.2 mmol) and MeCN (1.0 mL). The crude was purified by column chromatography and eluted with hexane−AcOEt (8:1). This afforded the title compound as a colorless oil (17.8 mg, 60% yield). ^1H NMR (400 MHz, $CDCl_3$): δ 7.42−7.37 (m, 2H), 7.00 (t, J = 7.6 Hz, 1H), 6.94 (d, J = 8.2 Hz, 1H), 6.13 (d, J = 47.4 Hz, 1H), 3.87 (s, 3H), 3.80 (s, 3H); $^{13}C\{^1H\}$ NMR (100 MHz, $CDCl_3$): δ 169.5 (d, J = 27.8 Hz), 157.2 (d, J = 3.8 Hz), 131.4 (d, J = 2.9 Hz), 129.2 (d, J = 4.8 Hz), 122.7 (d, J = 19.2 Hz), 120.8 (d, J = 1.9 Hz), 111.2, 84.8 (d, J = 181.1 Hz), 55.7, 52.5; ^{19}F NMR (376 MHz, $CDCl_3$): −178.1 (d, J = 47.6 Hz, 1F); HRMS (EI) *m/z* Calcd for $C_{10}H_{11}FO_3$ $[M]^+$ 198.0692 found 198.0692.

The general procedure B was followed with methyl 2-bromo-2-(2-methoxyphenyl)acetate (38.8 mg, 0. 15 mmol), AgF (38.1 mg, 0.30 mmol), $Et_3N \cdot 3HF$ (74 μL, 0.45 mmol) and MeCN (1.0 mL), giving the title compound (22.6 mg, 76% yield).

The scale up reaction of methyl 2-bromo-2-(2-methoxyphenyl)acetate (1g): The general procedure B was followed with methyl 2-bromo-2-(2-methoxyphenyl)acetate (1.04 g, 4.0 mmol), AgF (1.01 g, 8.0 mmol), $Et_3N \cdot 3HF$ (2.02 mL, 12.0 mmol) and MeCN (26 mL), giving the title

compound (745 mg, 94% yield).

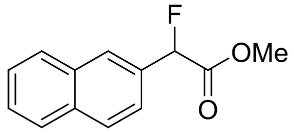

Synthesis of methyl 2-fluoro-2-(naphthalen-2-yl)acetate (2h): The general procedure A was followed with methyl 2-bromo-2-(naphthalen-2-yl)acetate (41.9 mg, 0.15 mmol), K_3PO_4 (38.2 mg, 0.18 mmol), $Et_3N·3HF$ (202 μL, 1.2 mmol) and MeCN (1.0 mL). The crude was purified by column chromatography and eluted with hexane−AcOEt (8:1). This afforded the title compound as a white solid (15.9 mg, 49% yield). ^{1}H NMR (500 MHz, $CDCl_3$): 7.96 (s, 1H), 7.91−7.86 (m, 3H), 7.58−7.53 (m, 3H), 5.98 (d, J = 47.7 Hz, 1H), 3.80 (s, 3H); ^{13}C{^{1}H} NMR (125 MHz, $CDCl_3$): δ 169.0 (d, J = 28.4 Hz), 133.7, 132.9, 131.4 (d, J = 19.9 Hz), 128.8, 128.3, 127.8, 127.0, 126.7, 126.6 (d, J = 6.6 Hz), 123.4 (d, J = 5.7 Hz), 89.5 (d, J = 184.7 Hz), 52.7; ^{19}F NMR (376 MHz, $CDCl_3$): −179.4 (d, J = 47.7 Hz, 1F); HRMS (EI) *m/z* Calcd for $C_{13}H_{11}FO_2$ [M]$^+$ 202.0197 found 202.0197.

The general procedure B was followed with methyl 2-bromo-2-(naphthalen-2-yl)acetate (41.9 mg, 0. 15 mmol), AgF (38.1 mg, 0.30 mmol), $Et_3N·3HF$ (74 μL, 0.45 mmol) and MeCN (1.0 mL), giving the title compound (22.0 mg, 67% yield).

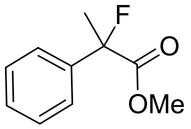

Synthesis of methyl 2-fluoro-2-phenylpropanoate (2i): The general procedure A was followed with methyl 2-bromo-2-phenylpropanoate (36.4 mg, 0.15 mmol), K_3PO_4 (38.2 mg, 0.18 mmol), $Et_3N·3HF$ (202 μL, 1.2 mmol) and MeCN (1.0 mL). The crude was purified by column chromatography and eluted with hexane−AcOEt (8:1). This afforded the title compound as a colorless oil (3.8 mg, 14% yield). ^{1}H NMR (500 MHz, $CDCl_3$): δ 7.52−7.45 (m, 2H), 7.42−7.36 (m, 3H), 3.78 (s, 3H), 1.95 (d, J = 22.2 Hz, 3H); ^{13}C{^{1}H} NMR (100 MHz, $CDCl_3$): δ 171.4 (d, J = 26.8 Hz), 139.1 (d, J = 22.0 Hz), 128.6, 128.5(d, J = 1.9 Hz), 124.6, 94.7 (d, J = 185.9 Hz), 52.9, 24.8 (d, J = 24.0 Hz); ^{19}F NMR (376 MHz, $CDCl_3$): −151.5 (q, J =23.0 Hz, 1F); HRMS (EI) *m/z* Calcd for $C_{10}H_{11}FO_2$ [M]$^+$ 182.0743 found 182.0743.

The general procedure B was followed with methyl 2-bromo-2-phenylpropanoate (36.4 mg, 0.15 mmol), AgF (38.1 mg, 0.30 mmol), $Et_3N·3HF$ (74 μL, 0.45 mmol) and MeCN (1.0 mL), giving the title compound (17.8 mg, 65% yield).

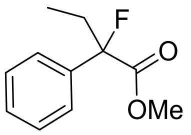

Synthesis of methyl 2-fluoro-2-phenylbutanoate (2j): The general procedure B was followed with methyl 2-bromo-2-phenylbutanoate (38.6 mg, 0.15 mmol), AgF (38.1 mg, 0.30 mmol), $Et_3N{\cdot}3HF$ (74 μL, 0.45 mmol) and MeCN (1.0 mL). The crude was purified by column chromatography and eluted with benzene. This afforded the title compound as a colorless oil (17.8 mg, 47% yield). ^{1}H NMR (400 MHz, $CDCl_3$): δ 7.51 (d, J = 7.6 Hz, 2H), 7.41−7.33 (m, 3H), 3.78 (s, 3H), 2.48−2.32 (m, 1H), 2.26−2.12 (m, 1H), 0.97 (t, J = 7.3 Hz, 3H); $^{13}C\{^1H\}$ NMR (100 MHz, $CDCl_3$): δ 171.2 (d, J = 26.8 Hz), 138.1 (d, J = 22.1 Hz), 128.5 (d, J = 1.9 Hz), 124.8, 124.7, 97.6 (d, J = 189.8 Hz), 52.9, 31.6 (d, J = 22.0 Hz), 7.60 (d, J = 3.8 Hz); ^{19}F NMR (376 MHz, $CDCl_3$): δ −167.2 (dd, J = 21.4. 27.1 Hz, 1F); HRMS (EI) m/z Calcd for $C_{11}H_{13}FO_2$ $[M]^+$ 196.0900 found 196.0900.

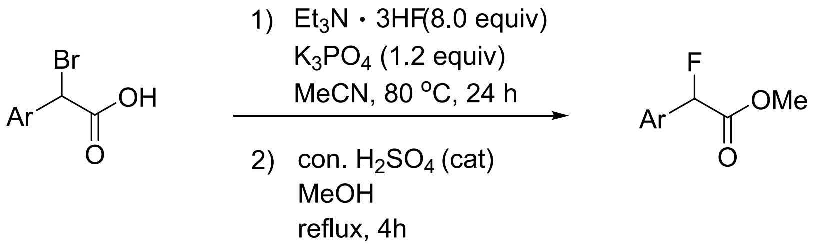

Synthesis of 2-fluoro-2-phenylacetic acid (2k−m): A 15 mL test tube quipped with a magnetic stirring bar was charged with 2-bromo-2-arylacetic acid (32.2mg, 0.15 mmol), K_3PO_4 (76.4 mg, 0.18 mmol), $Et_3N{\cdot}3HF$ (202 μL, 1.2 mmol) and DME (1.0 mL). The resulting mixture was stirred at 80 °C for 24 h. The reaction was quenched with saturated aqueous NH_4Cl after cooling to room temperature, the crude mixture was extracted with AcOEt. The combined organic layers were washed with brine, dried over $MgSO_4$ and evaporated in vacuo. The residue was dissolved in MeOH (2.0 mL), added con. H_2SO_4 (5.0 μL). The resulting mixture was stirred at 65 °C for 4 h. The reaction was quenched with saturated aqueous $NaHCO_3$ after cooling to room temperature; the crude mixture was extracted with AcOEt. The combined organic layers were washed with brine, dried over $MgSO_4$ and evaporated in vacuo. The residue was purified by preparative column chromatography on SiO_2 gel and eluted with hexane-AcOEt, affording the corresponding methyl ester.

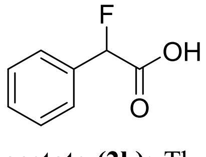

Synthesis of methyl 2-phenyl)-2-fluoroacetate (2k): The general procedure A was followed with 2-bromo-2-phenylacetic acid (32.2 mg, 0.15 mmol), K_3PO_4 (38.2 mg, 0.18 mmol), $Et_3N{\cdot}3HF$ (202 μL, 1.2 mmol) and DME (1.0 mL). The solvent was removed *in vacuo* after working out and NMR analysis was performed for the crude. The yield for **2k** was determined by ^{19}F NMR using fluorobenzene (–113 ppm) as an internal standard (62% yield). The crude undergoes the esterification with MeOH (2.0 mL) and con. H_2SO_4 (5.0 μL). This afforded the corresponding

methyl ester (15.6 mg, 62% yield).

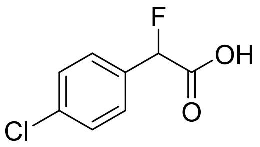

Synthesis of methyl 2-(4-chlorophenyl)-2-fluoroacetate (2l): The general procedure A was followed with 2-bromo-2-(4-chlorophenyl)acetic acid (37.4 mg, 0.15 mmol), K_3PO_4 (38.2 mg, 0.18 mmol), $Et_3N{\cdot}3HF$ (202 μL, 1.2 mmol) and DME (1.0 mL). The crude undergoes the esterification with MeOH (2.0 mL) and con. H_2SO_4 (5.0 μL). This afforded the corresponding methyl ester (16.2 mg, 53 % yield).

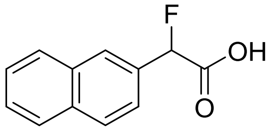

Synthesis of 2-fluoro-2-(naphthalen-2-yl)acetic acid (2m): The general procedure A was followed with 2-bromo-2-(naphthalen-2-yl)acetic acid (46.2 mg, 0.15 mmol), K_3PO_4 (38.2 mg, 0.18 mmol), $Et_3N{\cdot}3HF$ (202 μL, 1.2 mmol) and DME (1.0 mL). The yield for **2m** was determined by ^{19}F NMR using fluorobenzene (–113 ppm) as an internal standard (48% yield). The crude undergoes the esterification with MeOH (2.0 mL) and con. H_2SO_4 (5.0 μL). This afforded the corresponding methyl ester (15.7 mg, 48 % yield).

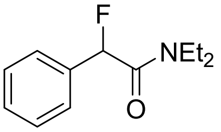

Synthesis of *N,N*-diethyl-2-fluoro-2-phenylacetamide (2n): The general procedure B was followed with *N,N*-diethyl-2-bromo-2-phenylacetamide (40.5 mg, 0.15 mmol), AgF (38.1 mg, 0.30 mmol), $Et_3N{\cdot}3HF$ (74 μL, 0.45 mmol) and MeCN (1.0 mL). The crude was purified by column chromatography and eluted with hexane−AcOEt (4:1). This afforded the title compound as a colorless oil (23.2 mg, 74% yield). ^1H NMR (400 MHz, $CDCl_3$): δ 7.47−7.40 (m, 5H), 6.02 (d, J = 49.7 Hz, 1H), 3.44−3.38 (m, 2H), 3.29−3.11 (m, 2H), 1.15 (t, J = 7.1 Hz, 3H), 1.00 (t, J = 7.1 Hz, 3H); $^{13}C\{^1H\}$ NMR (100 MHz, $CDCl_3$): δ 166.9 (d, J = 22.0 Hz), 134.7 (d, J = 20.1 Hz), 129.3 (d, J = 2.9 Hz), 128.9, 126.8 (d, J = 5.8 Hz), 90.5 (d, J = 182.1 Hz), 41.1 (d, J = 3.8 Hz), 40.4, 13.8, 12.5; ^{19}F NMR (376 MHz, $CDCl_3$): δ −172.3 (d, J = 49.3 Hz, 1F); HRMS (FAB) *m/z* Calcd for $C_{12}H_{17}FNO$ $[M+H]^+$ 210.1294 found 210.1294.

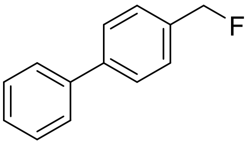

Synthesis of 4-(fluoromethyl)-1,1'-biphenyl (2o): The general procedure B was followed with 4-(bromomethyl)-1,1'-biphenyl (37.1 mg, 0.15 mmol), AgF (38.1 mg, 0.30 mmol), $Et_3N{\cdot}3HF$ (74 µL, 0.45 mmol) and MeCN (1.0 mL). The crude was purified by column chromatography and eluted with hexane−AcOEt (20:1). This afforded the title compound as a white solid (16.2 mg, 58% yield). ^{1}H NMR (400 MHz, $CDCl_3$): δ 7.65−7.61 (m, 4H), 7.49−7.45 (m, 4H), 7.40 (t, *J* = 7.1 Hz, 1H), 5.44 (d, *J* = 47.8 Hz); ^{13}C{^{1}H} NMR (100 MHz, $CDCl_3$): δ 141.7 (d, *J* = 2.9 Hz), 140.6, 135.1 (d, *J* = 17.3 Hz), 128.8, 128.1 (d, *J* = 5.7 Hz), 127.5, 127.3 (d, *J* = 1.9 Hz), 127.1, 84.4 (d, *J* = 164.8 Hz); ^{19}F NMR (376 MHz, $CDCl_3$): δ −206.0 (t, *J* = 47.6 Hz, 1F); HRMS (EI) *m/z* Calcd for $C_{13}H_{11}F$ $[M]^+$ 186.0845 found 186.0841.

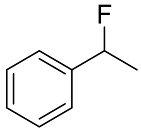

Synthesis of (1-fluoroethyl)benzene (2p): The general procedure A was followed with (1-bromoethyl)benzene (27.8 mg, 0.15 mmol), K_3PO_4 (38.2 mg, 0.18 mmol), $Et_3N{\cdot}3HF$ (202 µL, 1.2 mmol) and MeCN (1.0 mL). The solvent was removed *in vacuo* after working out and NMR analysis was performed for the crude. The yield of product **2p** was determined by ^{19}F NMR using fluorobenzene (–113 ppm) as an internal standard (13% yield).

The general procedure B was followed with (1-bromoethyl)benzene (27.8 mg, 0.15 mmol), AgF (38.1 mg, 0.30 mmol), $Et_3N{\cdot}3HF$ (74 µL, 0.45 mmol) and MeCN (1.0 mL), giving the title compound (50% ^{19}F NMR yield).

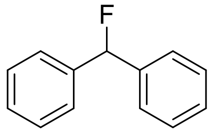

Synthesis of (fluoromethylene)dibenzene (2q): The general procedure B was followed with 4-(bromomethylene)dibenzene (37.1 mg, 0.15 mmol), AgF (38.1 mg, 0.30 mmol), $Et_3N{\cdot}3HF$ (74 µL, 0.45 mmol) and MeCN (1.0 mL). The crude was purified by column chromatography and eluted with hexane−AcOEt (20:1). This afforded the title compound as a white solid (12.7 mg, 45% yield). ^{1}H NMR (400 MHz, $CDCl_3$): δ 7.41−7.33 (m, 10H), 6.49 (d, *J* = 47.4 Hz, 1H); ^{13}C{^{1}H} NMR (100 MHz, $CDCl_3$): δ 139.8 (d, *J* = 21.1 Hz), 128.5, 128.4 (d, *J* = 1.9 Hz), 126.6 (d, *J* = 5.8 Hz), 94.4 (d, *J* = 171.5 Hz); ^{19}F NMR (376 MHz, $CDCl_3$): δ −166.6 (d, *J* = 47.7 Hz, 1F); HRMS (EI) *m/z* Calcd for $C_{13}H_{11}F$ $[M]^+$ 186.0845 found 186.0836.

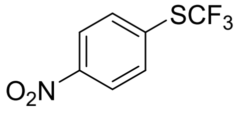

Synthesis of (4-nitrophenyl)(trifluoromethyl)sulfane (5a): The general procedure B was followed with (bromodifluoromethyl)(4-nitrophenyl)sulfane (42.6 mg, 0.15 mmol), AgF (38.1 mg, 0.30

mmol), $Et_3N·3HF$ (74 μL, 0.45 mmol) and MeCN (1.0 mL). The crude was purified by column chromatography and eluted with hexane−AcOEt (50:1). This afforded the title compound as a wthie solid (19.8 mg, 59% yield). ^{1}H NMR (400 MHz, $CDCl_3$): δ 8.28 (d, J = 8.9 Hz, 2H), 7.84 (d, J = 8.7 Hz, 2H); $^{13}C\{^1H\}$ NMR (100 MHz, $CDCl_3$): δ 149.1, 136.1, 132.5, 128.9 (q, J = 307.6 Hz), 124.3; ^{19}F NMR (376 MHz, $CDCl_3$): δ −41.2 (s, 3F); HRMS (EI) *m/z* Calcd for $C_7H_4F_3NO_2S$ $[M]^+$ 222.9915 found 222.9915.

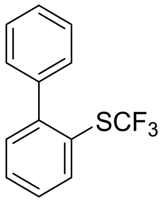

Synthesis of [1,1'-biphenyl]-2-yl(trifluoromethyl)sulfane (5b): The general procedure B was followed with [1,1'-biphenyl]-2-yl(bromodifluoromethyl)sulfane (47.3 mg, 0.15 mmol), AgF (38.1 mg, 0.30 mmol), $Et_3N·3HF$ (74 μL, 0.45 mmol) and MeCN (1.0 mL). The crude was purified by column chromatography and eluted with hexane−AcOEt (50:1). This afforded the title compound as a colorless oil (31.3 mg, 82% yield). ^{1}H NMR (400 MHz, $CDCl_3$): δ 7.81 (d, J = 7.8 Hz, 1H), 7.54−7.50 (m, 1H), 7.46−7.41 (m, 5H), 7.33−7.31 (m, 2H); $^{13}C\{^1H\}$ NMR (100 MHz, $CDCl_3$): δ 147.8, 140.2, 137.1, 131.3, 130.6, 129,7, 129.6 (q, J = 308.8 Hz), 129.6, 128.2, 127.9, 127.6, 123.1 (m); ^{19}F NMR (376 MHz, $CDCl_3$): δ −41.9 (s, 3F); HRMS (EI) *m/z* Calcd for $C_{13}H_9F_3S$ $[M]^+$ 254.0377 found 254.0375. These spectra are consistent with those of the previous report. [1]

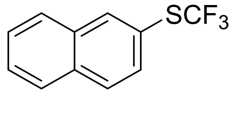

Synthesis of naphthalen-2-yl(trifluoromethyl)sulfane (5c): The general procedure B was followed with (bromodifluoromethyl)(naphthalen-2-yl)sulfane (43.3 mg, 0.15 mmol), AgF (38.1 mg, 0.30 mmol), $Et_3N·3HF$ (74 μL, 0.45 mmol) and MeCN (1.0 mL). The crude was purified by column chromatography and eluted with hexane−AcOEt (50:1). This afforded the title compound as a colorless oil (25.7 mg, 75% yield). ^{1}H NMR (400 MHz, $CDCl_3$): δ 8.21 (s, 1H), 7.90−7.88 (m, 3H), 7.67 (dd, J = 1.6, 8.7 Hz, 1H), 7.62−7.55 (m, 2H); $^{13}C\{^1H\}$ NMR (100 MHz, $CDCl_3$): δ 137.0, 134.4, 133.9, 131.8, 129.7 (q, J = 308.6 Hz), 129.2 , 128.2, 127.9, 127.8, 127.0, 121.5; ^{19}F NMR (376 MHz, $CDCl_3$): δ −41.2 (s, 3F); HRMS (EI) *m/z* Calcd for $C_{11}H_7F_3S$ $[M]^+$ 228.0221 found 228.0221.

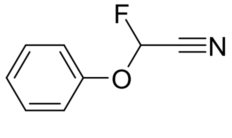

Synthesis of 2-fluoro-2-phenoxyacetonitrile (6a): The general procedure A was followed with 2-bromo-2-phenoxyacetonitrile (31.8 mg, 0.15 mmol), K_3PO_4 (38.2 mg, 0.18 mmol), $Et_3N·3HF$ (202

μL, 1.2 mmol) and MeCN (1.0 mL). The crude was purified by column chromatography and eluted with hexane−AcOEt (10:1). This afforded the title compound as a colorless oil (14.0 mg, 62% yield).
^{1}H NMR (500 MHz, $CDCl_3$): δ 7.42−7.39 (m, 2H), 7.27−7.24 (m, 1H), 7.16−7.14 (m, 2H), 6.25 (d, *J* = 55.4 Hz, 1H); ^{13}C{^{1}H} NMR (125 MHz, $CDCl_3$): δ 154.3 (d, *J* = 2.8 Hz), 130.1, 125.9, 118.4, 111.1 (d, *J* = 43.6 Hz), 95.6 (d, *J* = 224.5 Hz); ^{19}F NMR (376 MHz, $CDCl_3$): −116.3 (d, *J* = 54.9 Hz, 1F); HRMS (EI) *m/z* Calcd for C_8H_6FNO $[M]^+$ 151.0433 found 151.0433.
The general procedure B was followed with 2-bromo-2-phenoxyacetonitrile (31.8 mg, 0. 15 mmol), AgF (38.1 mg, 0.30 mmol), $Et_3N·3HF$ (74 μL, 0.45 mmol) and MeCN (1.0 mL), giving the title compound (15.2 mg, 67% yield).

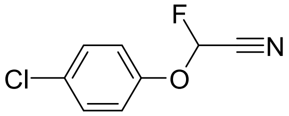

Synthesis of 2-(4-chlorophenoxy)-2-fluoroacetonitrile (6b): The general procedure A was followed with 2-bromo-2-(4-chlorophenoxy)acetonitrile (36.9 mg, 0.15 mmol), K_3PO_4 (38.2 mg, 0.18 mmol), $Et_3N·3HF$ (202 μL, 1.2 mmol) and MeCN (1.0 mL). The crude was purified by column chromatography and eluted with hexane−AcOEt (20:1). This afforded the title compound as a colorless oil (18.0 mg, 65% yield).
^{1}H NMR (400 MHz, $CDCl_3$): δ 7.39−7.35 (m, 2H), 7.12−7.08 (m, 2H), 6.22 (d, *J* = 54.9 Hz, 1H); ^{13}C{^{1}H} NMR (100 MHz, $CDCl_3$): δ 152.6 (d, *J* = 2.9 Hz), 131.4, 130.1, 120.0 (d, *J* = 1.9 Hz), 111.8 (d, *J* = 43.1 Hz), 95.1 (d, *J* = 226.2 H); ^{19}F NMR (376 MHz, $CDCl_3$): −117.2 (d, *J* = 54.9 Hz, 1F); HRMS (EI) *m/z* Calcd for C_8H_5ClFNO $[M]^+$ 185.0044 found 185.0044.
The general procedure B was followed with 2-bromo-2-(4-chlorophenoxy)acetonitrile (36.9 mg, 0. 15 mmol), AgF (38.1 mg, 0.30 mmol), $Et_3N·3HF$ (74 μL, 0.45 mmol) and MeCN (1.0 mL), giving the title compound (21.3 mg, 77% yield).

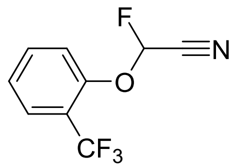

Synthesis of 2-fluoro-2-(2-(trifluoromethyl)phenoxy)acetonitrile (6c): The general procedure A was followed with 2-bromo-2-(2-(trifluoromethyl)phenoxy)acetonitrile (42.0 mg, 0.15 mmol), K_3PO_4 (38.2 mg, 0.18 mmol), $Et_3N·3HF$ (202 μL, 1.2 mmol) and MeCN (1.0 mL). The crude was purified by column chromatography and eluted with hexane−AcOEt (8:1). This afforded the title compound as a colorless oil (21.0 mg, 64% yield).
^{1}H NMR (400 MHz, $CDCl_3$): δ 7.71 (d, *J* = 7.8 Hz, 1H), 7.62 (t, *J* = 8.9 Hz, 1H), 7.40−7.33 (m, 2H), 6.25 (d, *J* = 54.9 Hz); ^{13}C{^{1}H} NMR (125 MHz, $CDCl_3$): δ 151.8, 133.8, 127.6 (q, *J* = 4.0 Hz), 125.9, 122.6 (q, *J* = 270.9 Hz), 122.0 (m), 119.2, 117.8, 111.4 (d, *J* = 42.2 Hz), 95.2 (d, *J* = 227.1 Hz); ^{19}F

NMR (376 MHz, $CDCl_3$): −61.4 (s, 3F), −117.3 (d, J = 54.3 Hz, 1F); HRMS (EI) *m/z* Calcd for $C_9H_5F_4NO$ $[M]^+$ 219.0307 found 219.0307.

The general procedure B was followed with 2-bromo-2-(2-(trifluoromethyl)phenoxy)acetonitrile (42.0 mg, 0.15 mmol), AgF (38.1 mg, 0.30 mmol), $Et_3N{\cdot}3HF$ (74 μL, 0.45 mmol) and MeCN (1.0 mL), giving the title compound (21.7 mg, 65% yield).

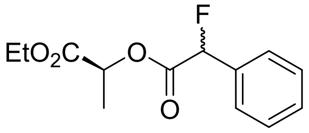

Synthesis of ethyl (2S)-2-(2-fluoro-2-phenylacetoxy)propanoate (8): The general procedure A was followed with ethyl (2S)-2-(2-bromo-2-phenylacetoxy)propanoate (47.3 mg, 0.15 mmol), K_3PO_4 (38.2 mg, 0.18 mmol), $Et_3N{\cdot}3HF$ (202 μL, 1.2 mmol) and MeCN (1.0 mL). The crude was purified by column chromatography and eluted with hexane−AcOEt (4:1). This afforded the title compound as a colorless oil (21.8 mg, 57% yield).

^{1}H NMR (400 MHz, $CDCl_3$): δ 7.53−7.50 (m, 2H), 7.45−7.42 (m, 3H), 5.88 (d, J = 47.6 Hz, 1/2H), 5.86 (d, J = 47.8 Hz, 1/2H), 5.21 (q, J = 7.1 Hz, 1/2H), 5.15 (q, J = 7.1 Hz, 1/2H), 4.19 (q, J = 7.1 Hz, 1H), 4.10 (dq, J = 1.8, 7.3 Hz, 1H), 1.53 (d, J = 6.9 Hz, 3/2H), 1.47 (d, J = 7.1 Hz, 3/2H), 1.24 (t, J = 7.3 Hz, 3/2H), 1.14 (t, J = 7.1 Hz, 3/2H); $^{13}C\{^1H\}$ NMR (100 MHz, $CDCl_3$): δ 169.9, 167.8 (d, J = 14.4 Hz), 133.9 (d, J = 20.1 Hz), 133.6 (d, J = 20.1 Hz), 129.7 (m), 128.8, 128.6, 127.0 (d, J = 5.8 Hz), 126.9 (d, J = 5.8 Hz), 89.2 (d, J = 185.9 Hz), 89.1 (d, J = 184.0 Hz), 69.7, 61.6, 61.5, 16.8, 16.7, 14.0, 13.9; ^{19}F NMR (376 MHz, $CDCl_3$): −179.2 (d, J = 47.6 Hz, 1/2F), −179.3 (d, J = 47.7 Hz, 1/2F); HRMS (EI) *m/z* Calcd for $C_{13}H_{16}FO_4$ $[M+H]^+$ 255.1033 found 255.1033.

The general procedure B was followed with ethyl (2S)-2-(2-bromo-2-phenylacetoxy)propanoate (47.3 mg, 0.15 mmol), AgF (38.1 mg, 0.30 mmol), $Et_3N{\cdot}3HF$ (74 μL, 0.45 mmol) and MeCN (1.0 mL), giving the title compound (26.6 mg, 70% yield).

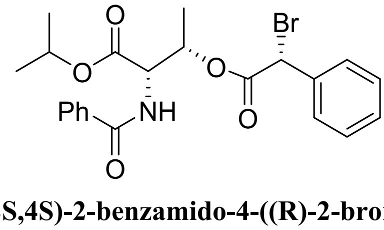

Synthesis of isopropyl (2S,4S)-2-benzamido-4-((R)-2-bromo-2-phenylacetoxy)pentanoate [(αR)-9] following a previous report [2]

A 100 mL round bottom flask quipped with a magnetic stirring bar was charged with N-benzoyl-l-threonine isopropyl ester (796 mg, 3.0 mmol), racemic 2-bromo-2-phenylacetic acid (645 mg, 3.0 mmol), DCC (680 mg, 2.0 mmol, 1.0 equiv), and DMAP (37 mg, 0.3 mmol, 0.1 equiv) in CH_2Cl_2 (30 mL). The resulting mixture was stirred at room temperature for 10 h. For the precipitate removal, the mixture was filtered through a glass filter, and washed with CH_2Cl_2. The organic layers were dried over $MgSO_4$ and evaporated in vacuo. The residue was purified by column chromatography on

SiO_2 gel and eluted with hexane-AcOEt (8:1 to 4:1), affording the title compound (846 mg, 61% yield) though crystallization induced dynamic resolution (CIDR) .

^{1}H NMR (500 MHz, $CDCl_3$): δ 7.76 (d, *J* = 7.3 Hz, 2H), 7.56−7.52 (m, 3H), 7.47 (t, *J* = 7.8 Hz, 2H), 7.38−7.35 (m, 3H), 6.65 (d, *J* = 9.1 Hz, 1H), 5.56 (dq, *J* = 2.7, 6.4 Hz, 1H), 5.33 (s, 1H), 4.98 (dd, *J* = 2.7, 9.0 Hz, 1H), 4.91 (sept, *J* = 6.4 Hz, 1H), 1.38 (d, *J* = 6.4 Hz, 3H), 1.23 (d, *J* = 6.1 Hz, 3H), 1.10 (d, *J* = 6.4 Hz, 3H); $^{13}C\{^1H\}$ NMR (125 MHz, $CDCl_3$): δ 168.9, 167.6, 167.0, 135.4, 133.6, 132.0, 129.4, 128.9, 128.7, 128.6, 127.1, 73.4, 70.2, 55.7, 46.7, 21.7, 21.4, 16.9; HRMS (FAB) *m/z* Calcd for $C_{22}H_{25}BrNO_5$ $[M+H]^+$ 462.0916 found 462.0916. These spectra are consistent with those of the previous report. [2]

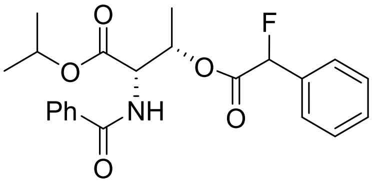

Synthesis of isopropyl (2S,4S)-2-benzamido-4-(2-fluoro-2-phenylacetoxy)pentanoate (10): The general procedure A was followed with isopropyl (2S,4S)-2-benzamido-4-((R)-2-bromo-2-phenylacetoxy)pentanoate (69.3 mg, 0.15 mmol), K_3PO_4 (38.2 mg, 0.18 mmol), $Et_3N{\cdot}3HF$ (202 μL, 1.2 mmol) and MeCN (1.0 mL). The diastereomeric ratio of the crude was determined by ^{19}F NMR using fluorobenzene (–113 ppm) as an internal standard (45 : 55 dr) (See, Figure S1). The crude was purified by column chromatography and eluted with hexane−AcOEt (4:1). This afforded the title compound as a colorless oil (49.3 mg, 81% yield).

Synthesis of isopropyl (2S,4S)-2-benzamido-4-((S)-2-fluoro-2-phenylacetoxy)pentanoate (10): The general procedure B was followed with isopropyl (2S,4S)-2-benzamido-4-((R)-2-bromo-2-phenylacetoxy)pentanoate (69.3 mg, 0.15 mmol), AgF (38.1 mg, 0.30 mmol), $Et_3N{\cdot}3HF$ (74 μL, 0.45 mmol) and MeCN (1.0 mL), giving the title compound (55.2 mg, 92% yield). The diastereomeric ratio of the crude was determined by ^{19}F NMR using fluorobenzene (–113 ppm) as an internal standard (83 : 17 dr) (See, Figure S1).

^{1}H NMR (400 MHz, $CDCl_3$, major diastereomer): δ 7.78 (d, *J* = 7.1 Hz, 2H), 7.55 (t, *J* = 7.3 Hz, 1H), 7.41−7.39 (m, 7H), 6.73 (d, *J* = 9.0 Hz, 1H), 5.77 (d, *J* = 47.6 Hz, 1H), 5.63−5.58 (m, 1H), 5.01 (dd, *J* = 3.8, 9.2 Hz, 1H), 4.97 (sept, *J* = 6.2 Hz, 1H), 1.28 (d, *J* = 6.4 Hz, 3H), 1.25 (d, *J* = 6.4 Hz, 3H), 1.10 (d, *J* = 6.4 Hz, 3H); $^{13}C\{^1H\}$ NMR (100 MHz, $CDCl_3$, major diastereomer): δ 168.9, 167.6, 167.4 (d, *J* = 25.9 Hz), 133.8 (d, *J* = 20.1 Hz), 133.6, 132.0, 129.7 (d, *J* = 2.9 Hz), 128.8, 128.7, 127.1, 126.5 (d, *J* = 5.8 Hz), 89.1 (d, *J* = 186.9 Hz), 72.7, 70.2, 55.7, 21.6, 21.3, 16.7; ^{19}F NMR (376 MHz, $CDCl_3$, major diastereomer): −180.3 (d, *J* = 47.7 Hz, 1F); HRMS (FAB) *m/z* Calcd for $C_{22}H_{125}FNO_5$ $[M+H]^+$ 402.1717 found 402.1717.

Synthesis of isopropyl (2S,4S)-2-benzamido-4-((S)-2-fluoro-2-phenylacetoxy)pentanoate (10): The general procedure C was followed with isopropyl (2S,4S)-2-benzamido-4-((R)-2-bromo-2-phenylacetoxy)pentanoate (69.3 mg, 0.15 mmol), AgF (38.1 mg, 0.30 mmol), and MeCN (1.0 mL). The diastereomeric ratio of the crude was determined by ^{19}F NMR using fluorobenzene (–113 ppm) as an internal standard (92 : 8 dr) (See, Figure S1).

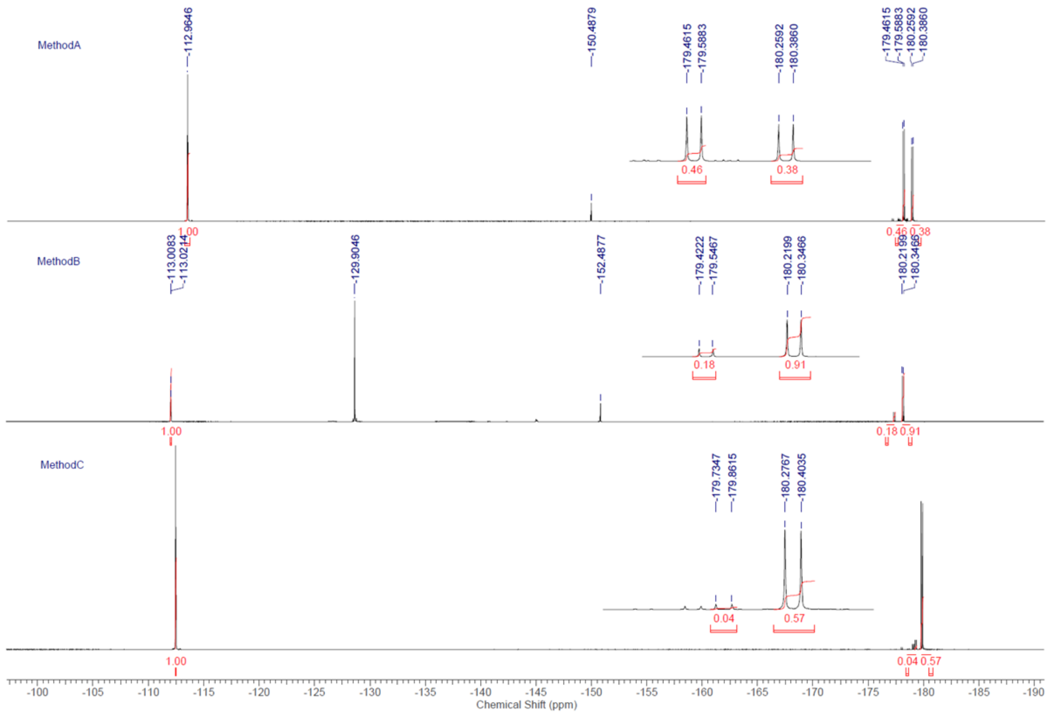

Figure S1. ^{19}F NMR spectrum of the crude mixture for each method.

4. Investigation on The Stability of $Et_3N{\cdot}3HF$

To investigate on the stability of $Et_3N{\cdot}3HF$ under reaction conditions of Method A and B, we demonstrated a study of ^{19}F NMR spectroscopy. A solution of $Et_3N{\cdot}3HF$ in d-acetonitrile (0.1 M) heated at 80 °C for 12 h. The ^{19}F NMR spectra showed that $Et_3N{\cdot}3HF$ (−166 ppm) completely decomposed to products such as $Et_3N{\cdot}2HF$, $Et_3N{\cdot}HF$, and HF (−152, −151 and −128 ppm) (Figure S1). The addition of K_3PO_4 decreased a signal of HF. Whereas AgF (0.1 mmol) is insoluble in d-acetonitrile. When $Et_3N{\cdot}3HF$ (0.1 mmol) was added, the solution turned to be clear. The signal of $Et_3N{\cdot}3HF$ and AgF (−163 ppm) in d-acetonitrile mostly remained after 16h, and the additional signals appeared at −128 ppm for HF and at −144 ppm for HF_2^- (Figure S2).

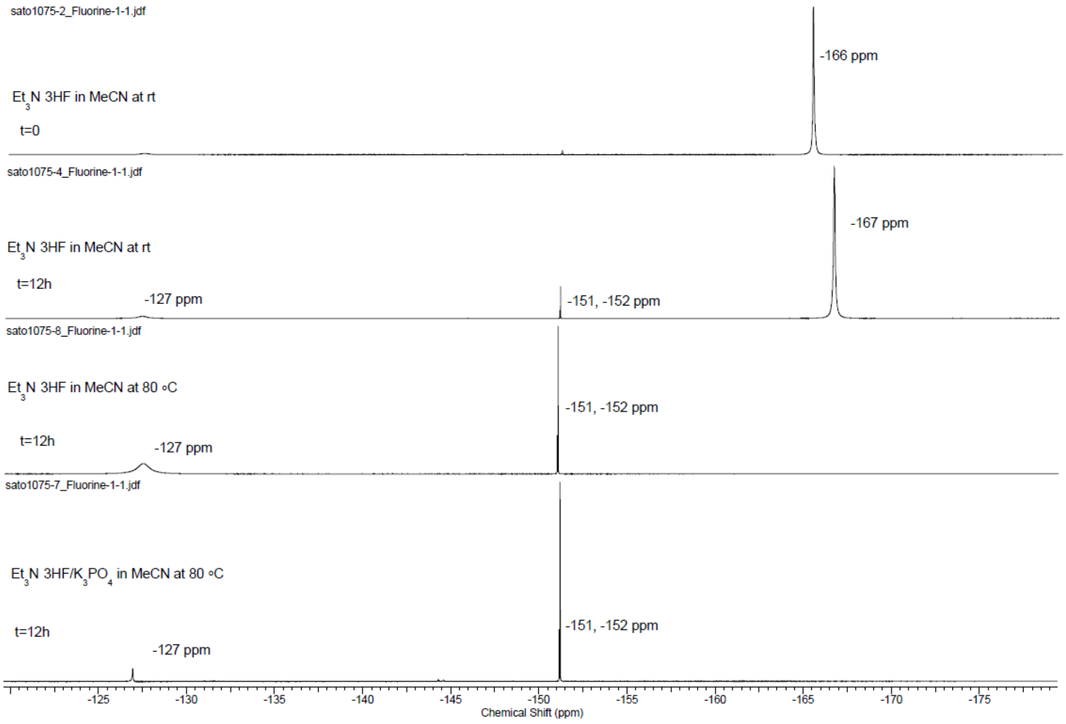

Figure S2. ^{19}F NMR spectra on the stability of $Et_3N{\cdot}3HF$ in the presence of K_3PO_4

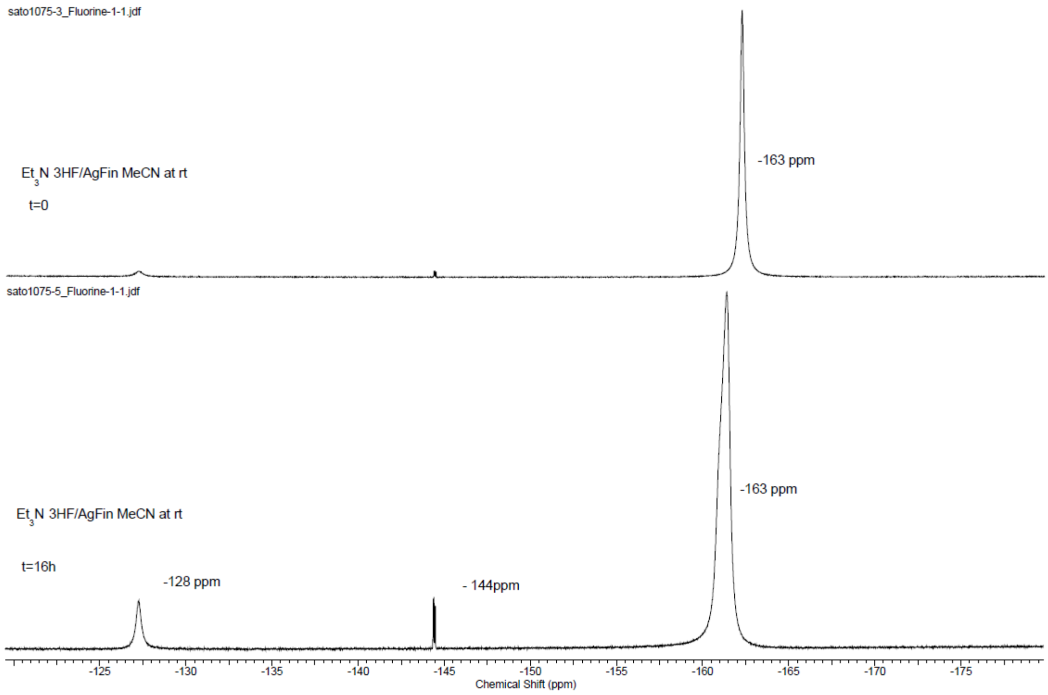

Figure S3. ^{19}F NMR spectra on the stability of a combination of $Et_3N{\cdot}3HF$ and AgF

5. References

1. D. J. Adams, A. Goddard, J. H. Clark, D. J. Macquarrie, Trifluoromethylthiodediazoniation: A Simple, Efficient Route to Trifluoromethyl Aryl Sulfides. Chem. Commun. 2000, 987-988.

2. K. J. Park, Y. Kim, M.-S. Lee, Y. S. Park, *N*-Benzoyl-L-Threonine-Isopropyl-Ester-Mediated Crystallization-Induced Dynamic Resolution of α-Bromo Arylacetates for the Asymmetric Synthesis of α-Thio and α-Oxy Arylacetates, Eur. J. Org. Chem. 2014, 1645.

6. NMR spectra

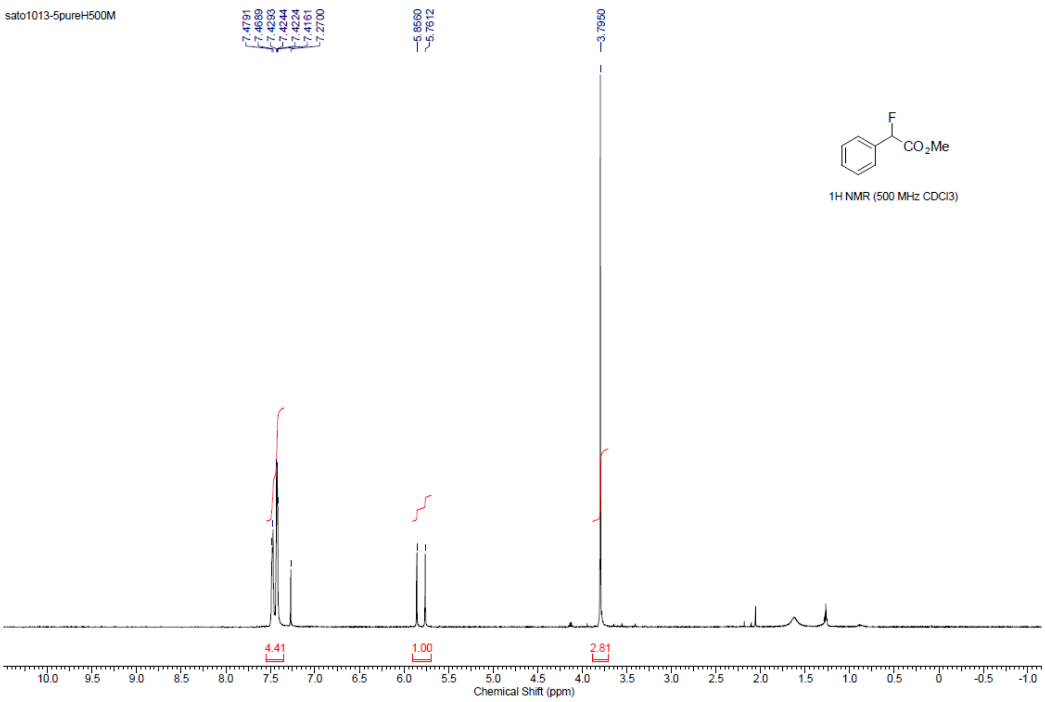

Figure S4. ^{1}H NMR of **2a** (500 MHz, $CDCl_3$)

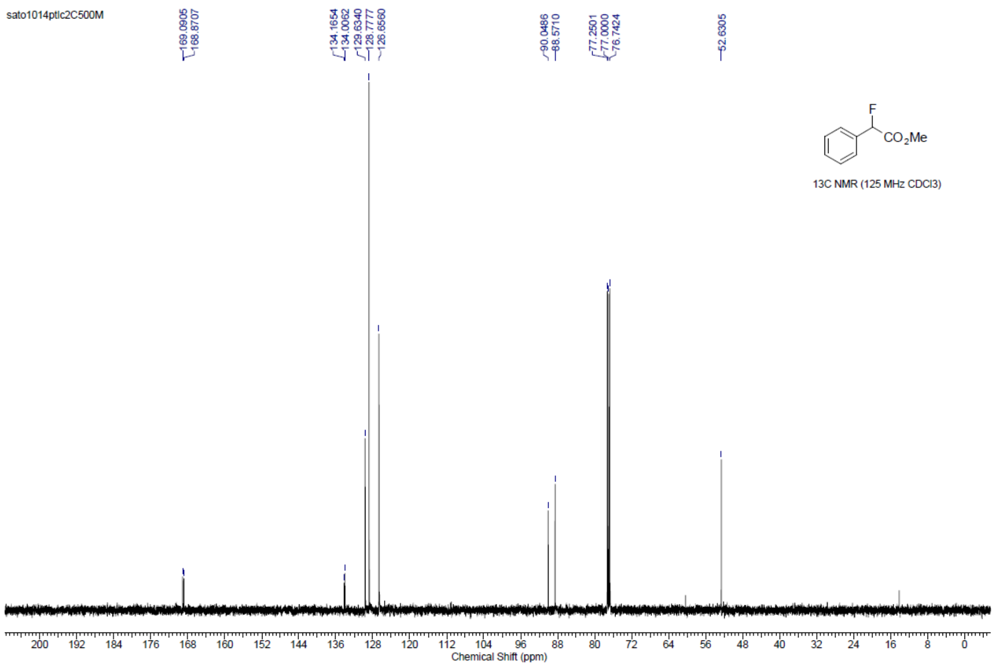

Figure S5. ^{13}C NMR of **2a** (125 MHz, $CDCl_3$)

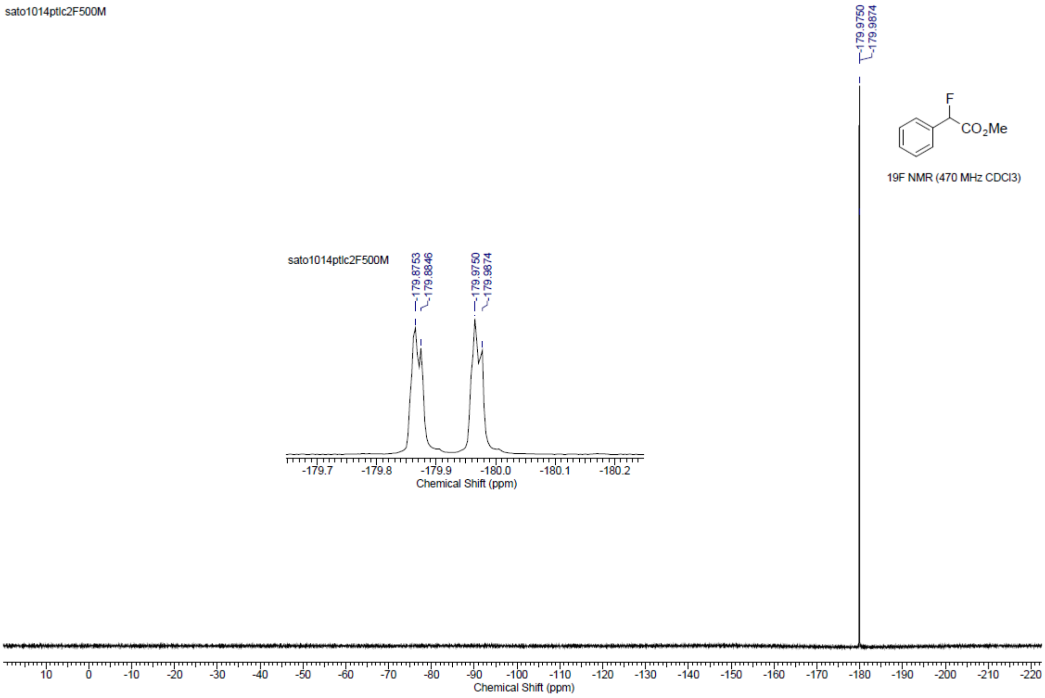

Figure S6. ^{19}F NMR of **2a** (470 MHz, $CDCl_3$)

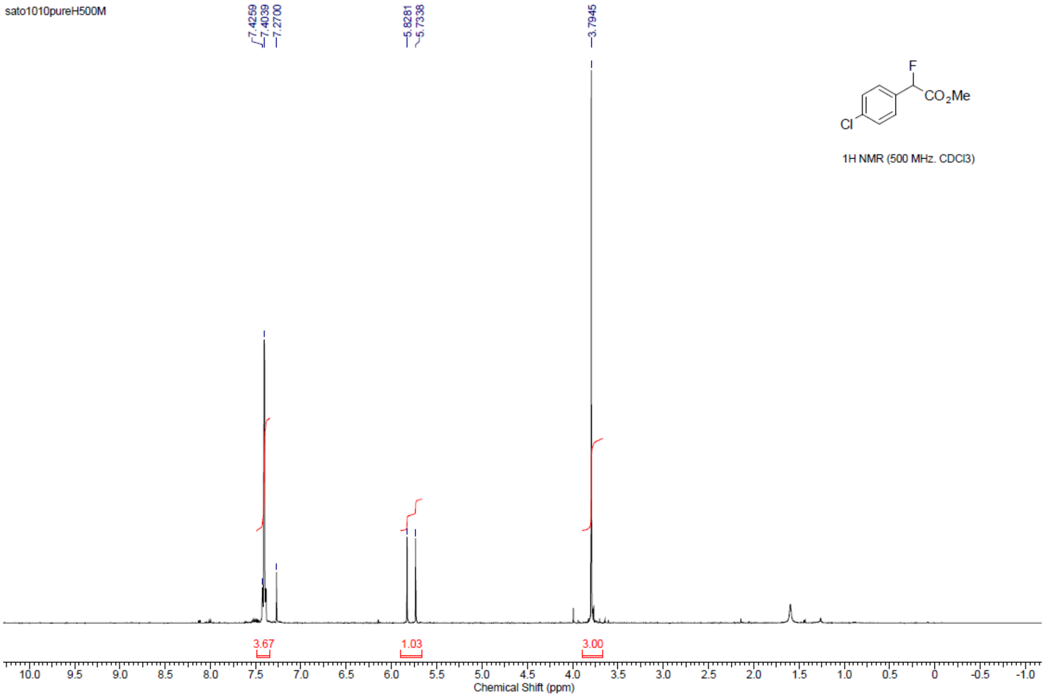

Figure S7. ^{1}H NMR of **2b** (500 MHz, $CDCl_3$)

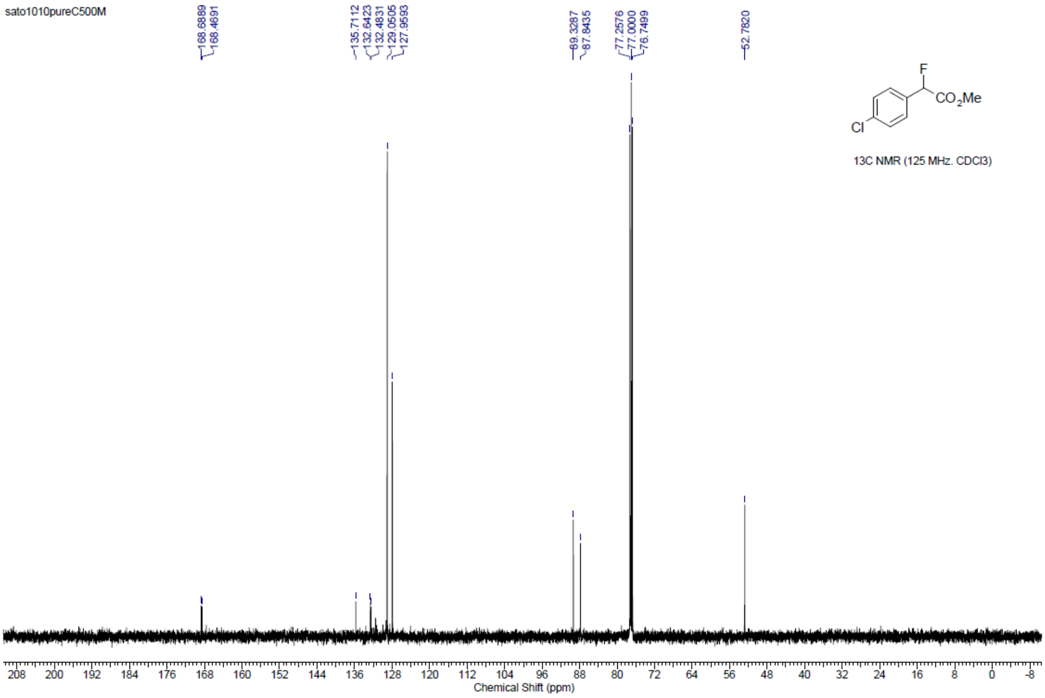

Figure S8. ^{13}C NMR of **2b** (125 MHz, $CDCl_3$)

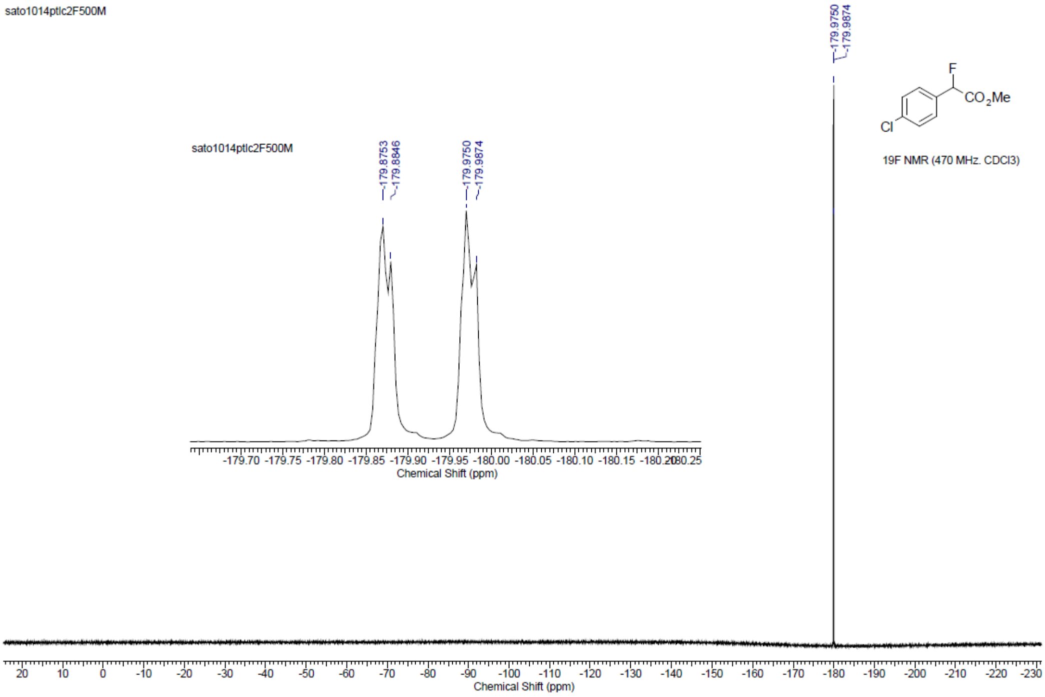

Figure S9. ^{19}F NMR of **2b** (470 MHz, $CDCl_3$)

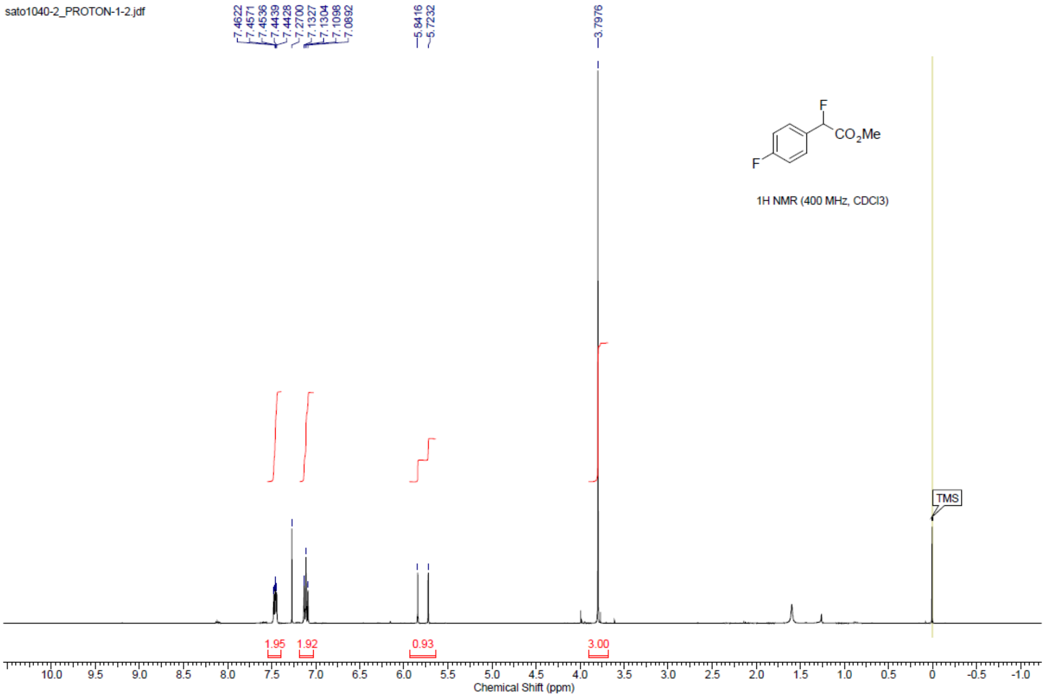

Figure S10. ^{1}H NMR of **2c** (400 MHz, $CDCl_3$)

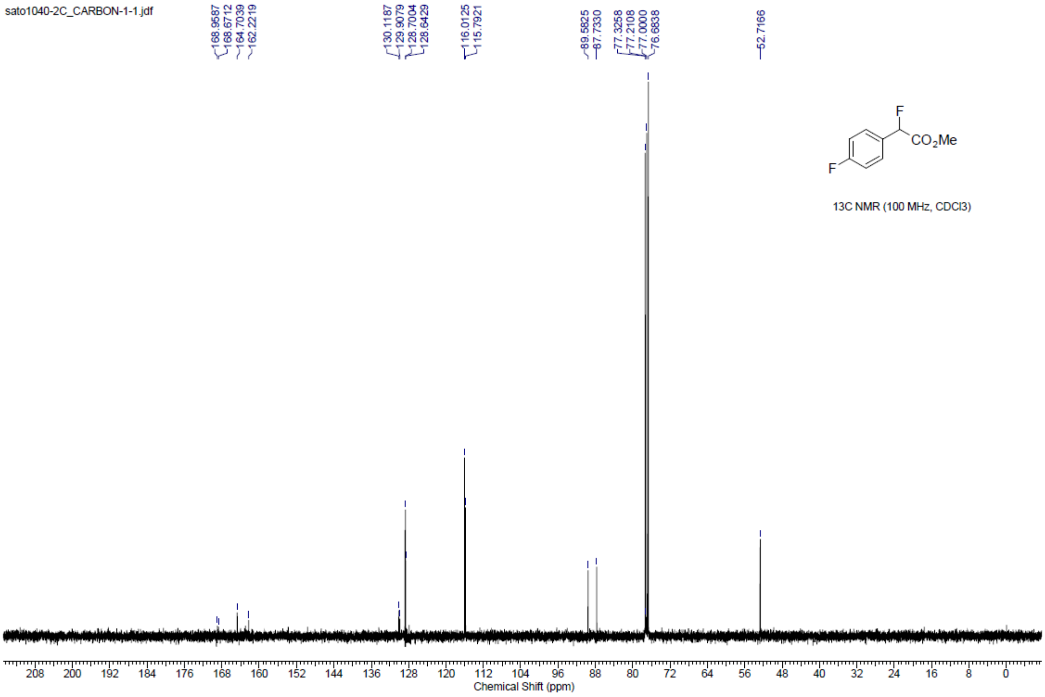

Figure S11. ^{13}C NMR of **2c** (100 MHz, $CDCl_3$)

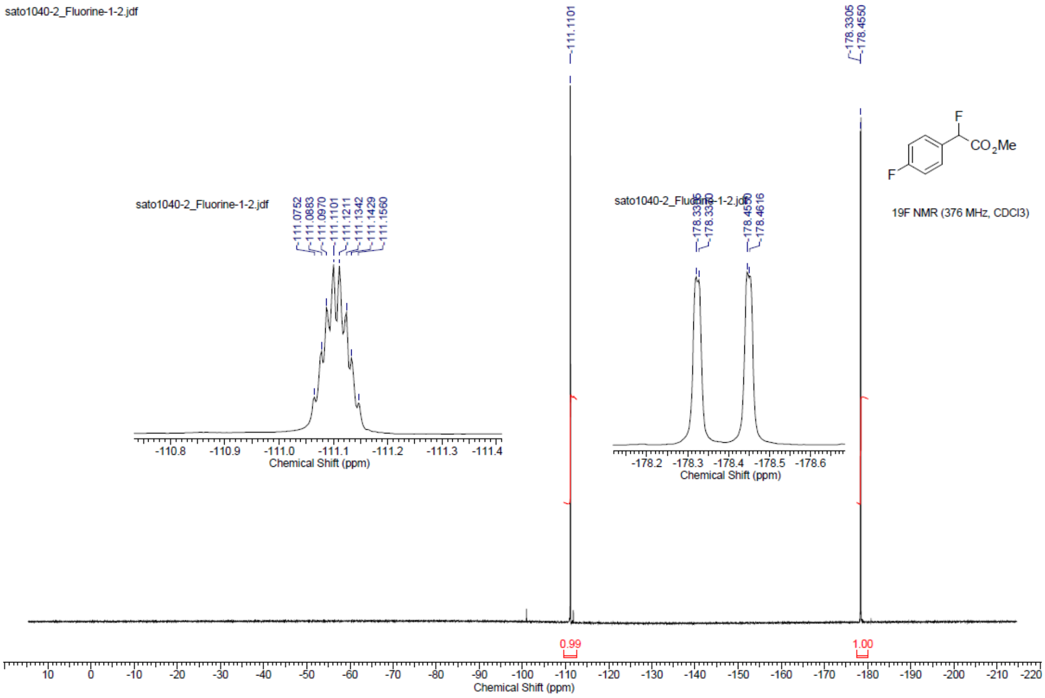

Figure S12. ^{19}F NMR of **2c** (376 MHz, $CDCl_3$)

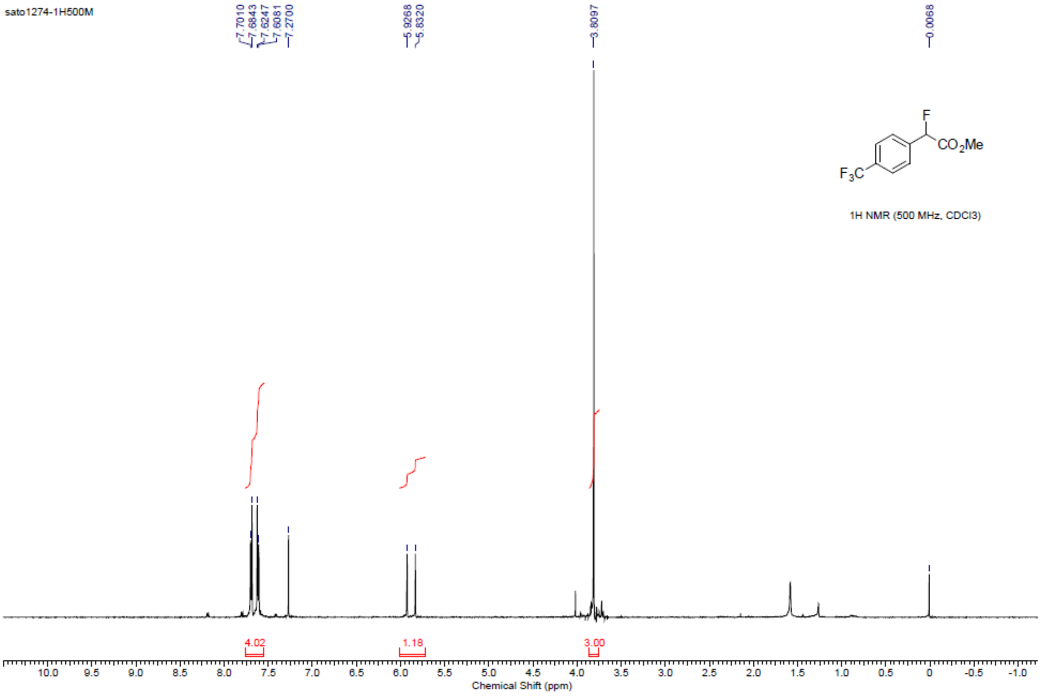

Figure S13. ^{1}H NMR of **2d** (500 MHz, $CDCl_3$)

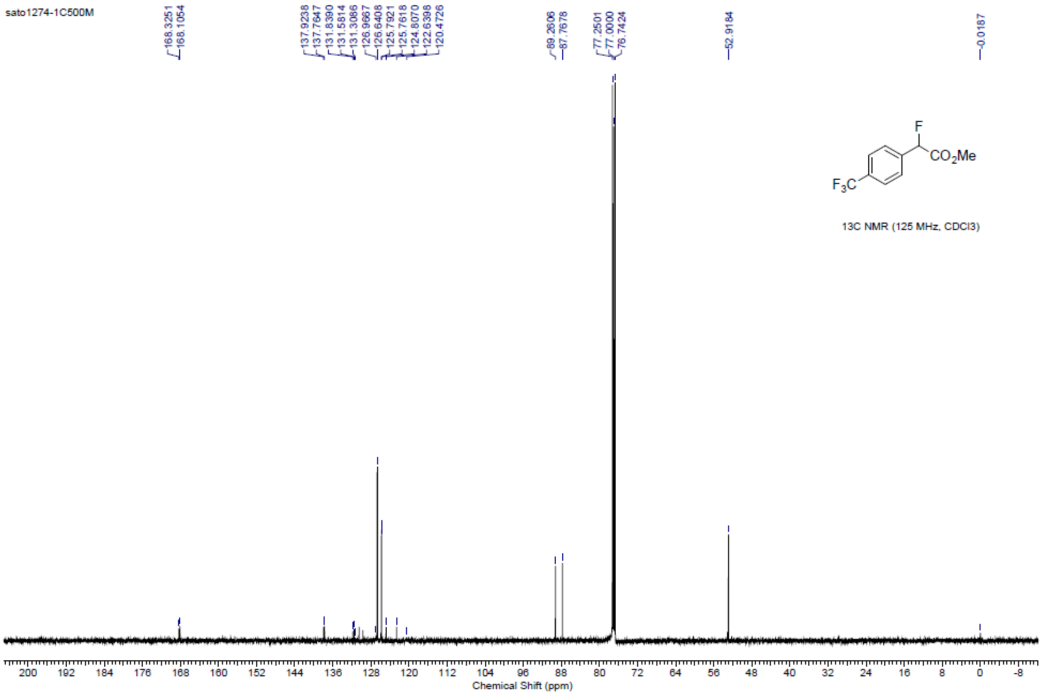

Figure S14. ^{13}C NMR of **2d** (125 MHz, $CDCl_3$)

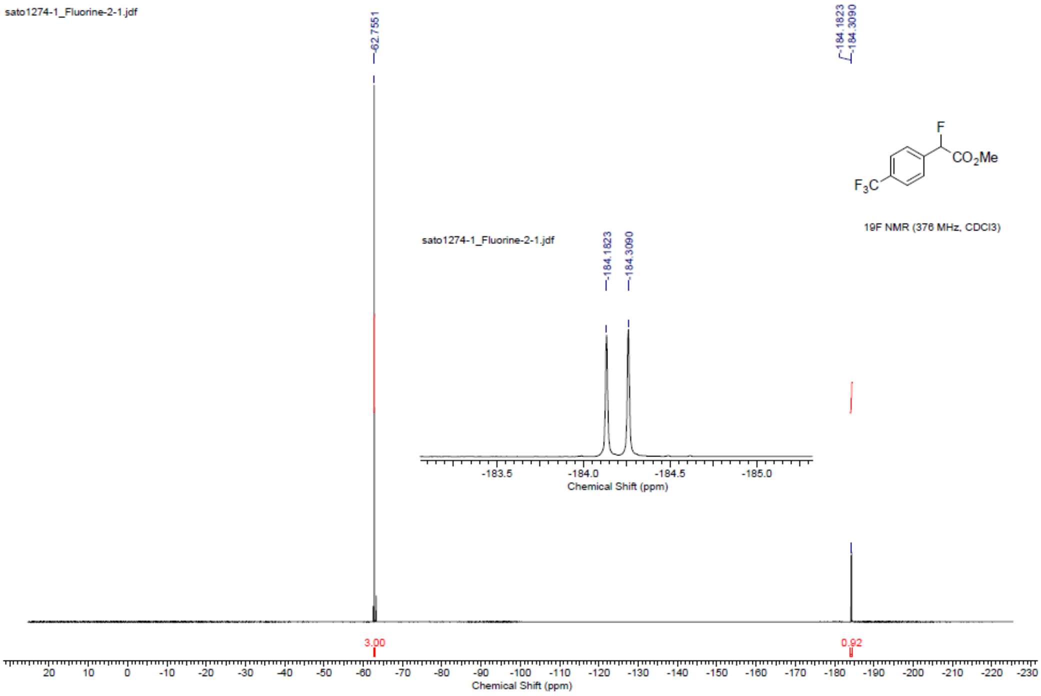

Figure S15. ^{19}F NMR of **2d** (376 MHz, $CDCl_3$)

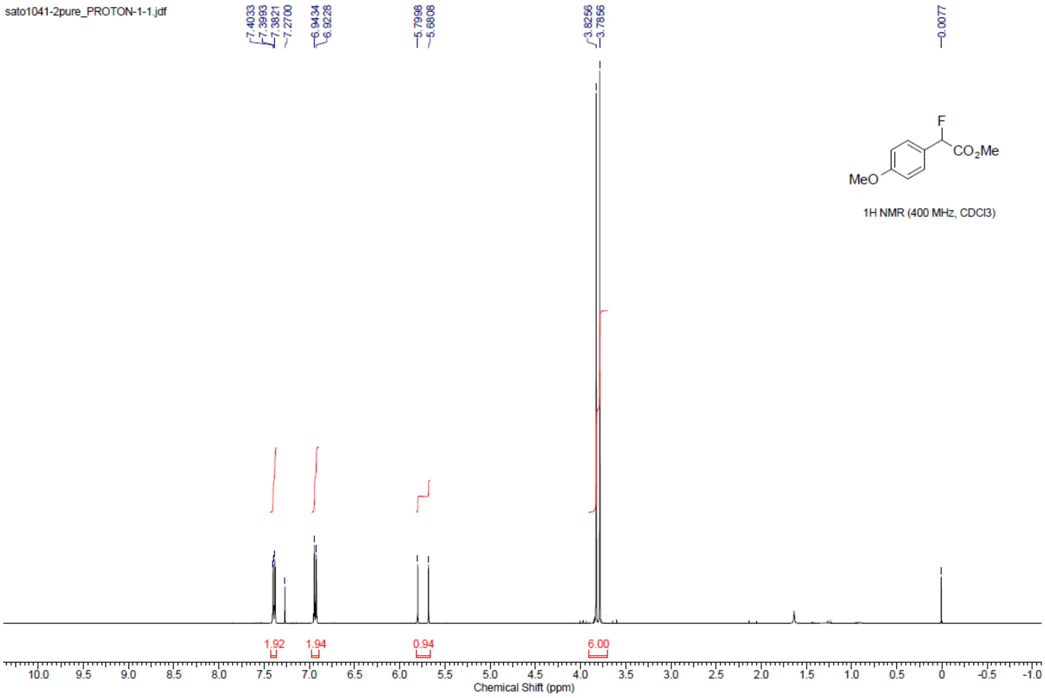

Figure S16. ^{1}H NMR of **2e** (400 MHz, $CDCl_3$)

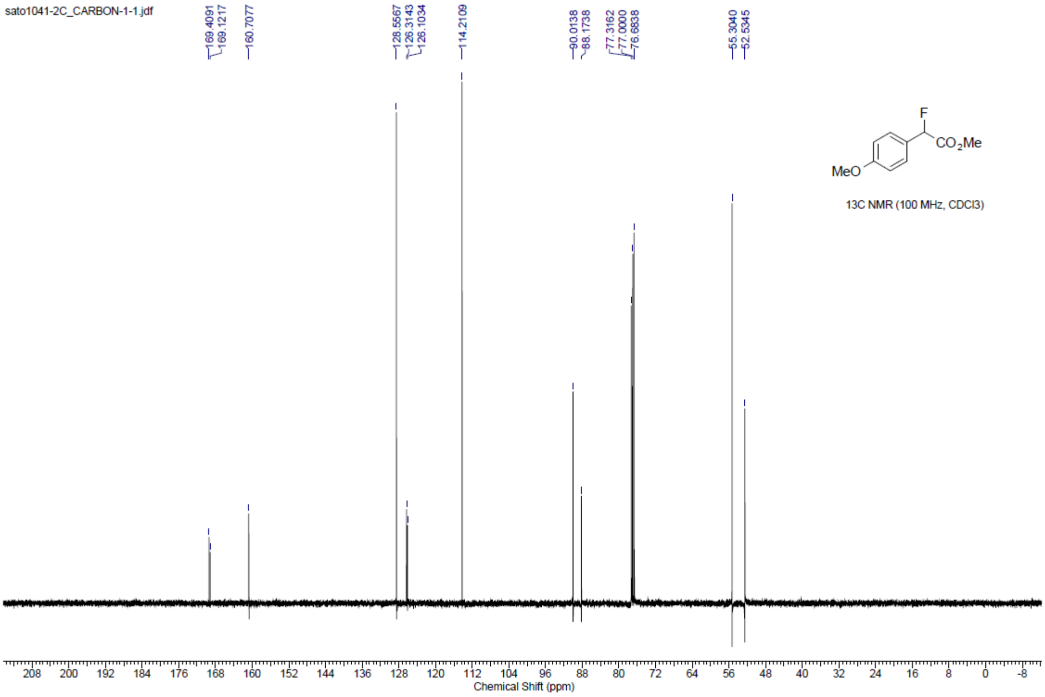

Figure S17. ^{13}C NMR of **2e** (100 MHz, $CDCl_3$)

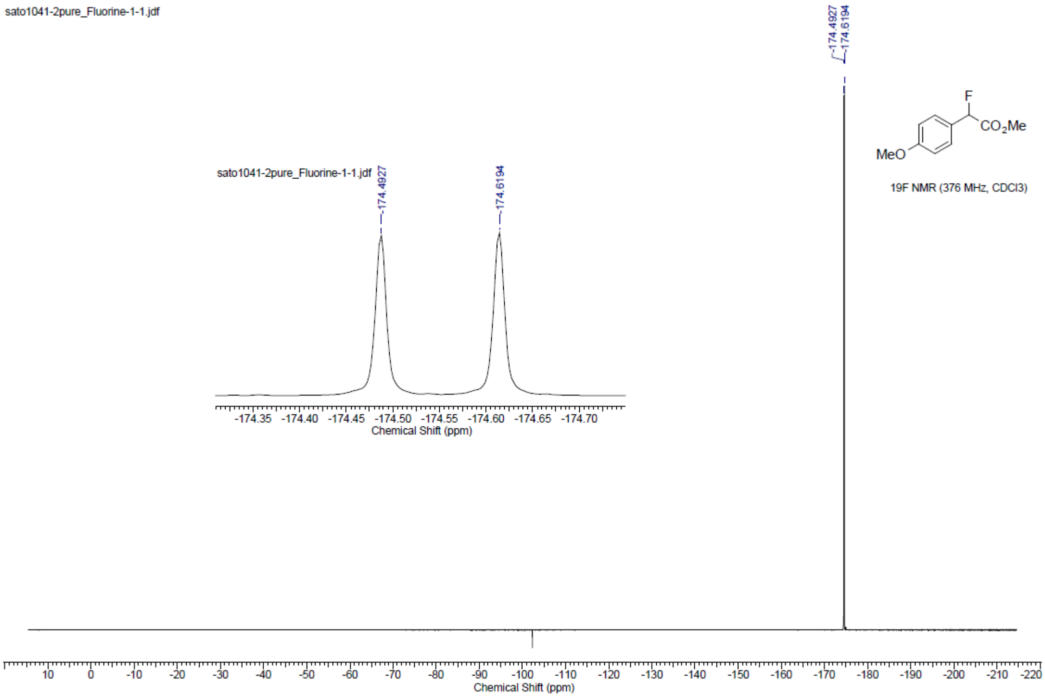

Figure S18. ^{19}F NMR of **2e** (376 MHz, $CDCl_3$)

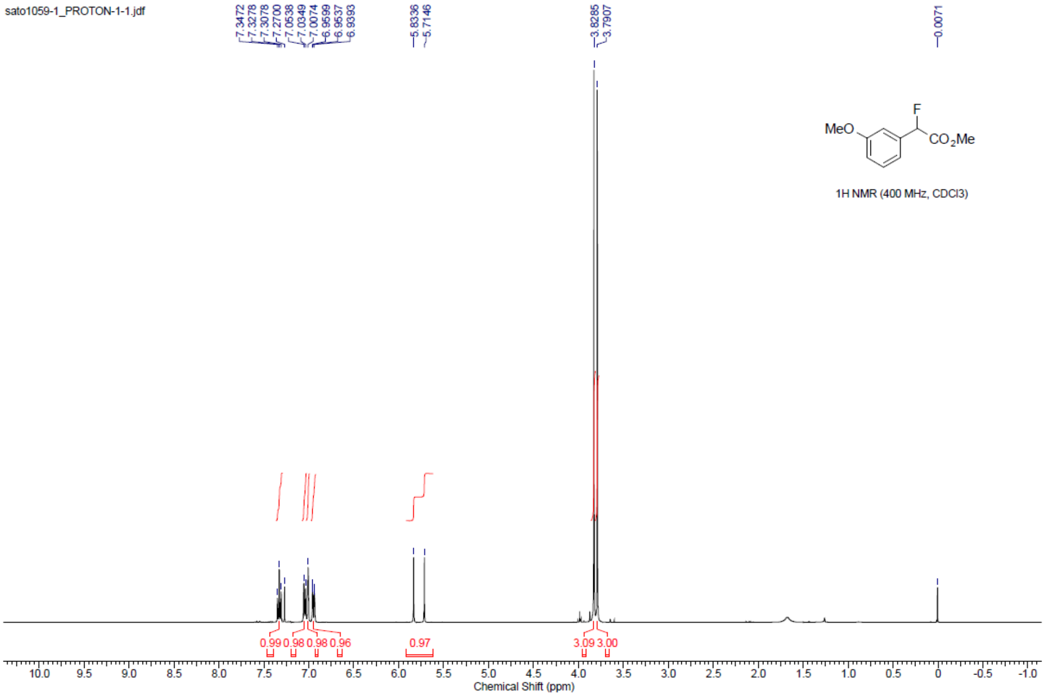

Figure S19. ^{1}H NMR of **2f** (400 MHz, $CDCl_3$)

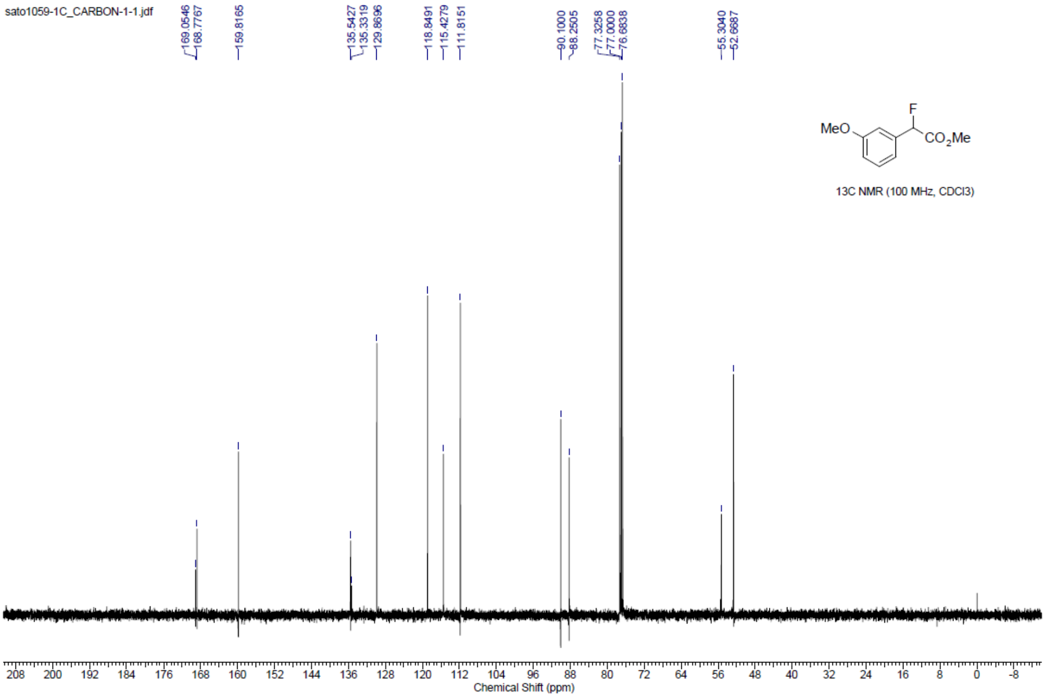

Figure S20. ^{13}C NMR of **2f** (100 MHz, $CDCl_3$)

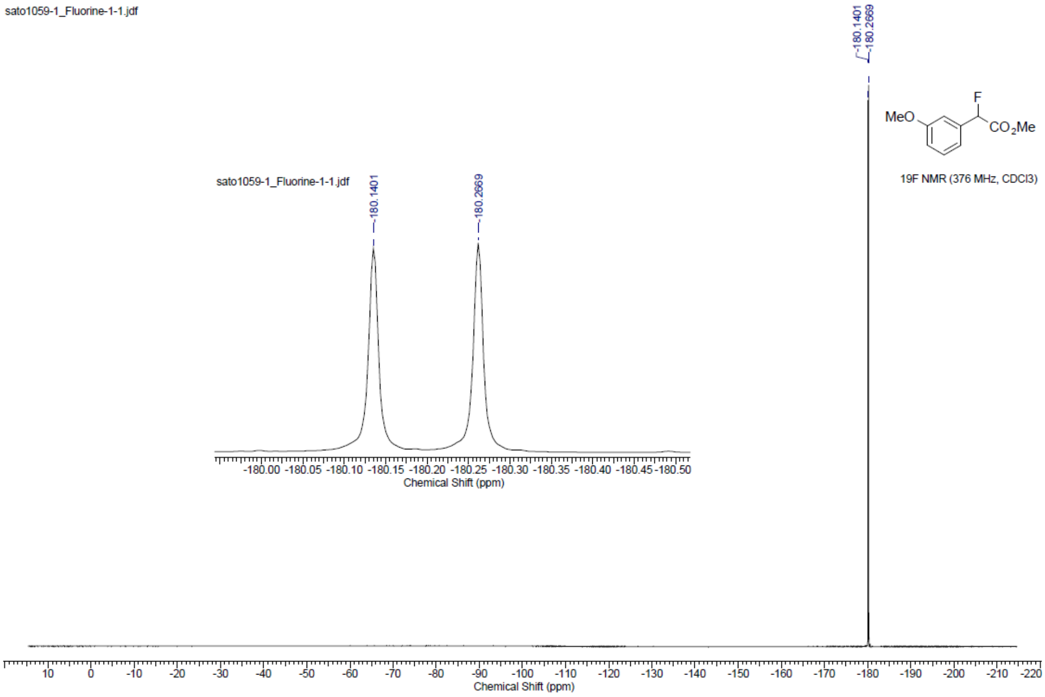

Figure S21. ^{19}F NMR of **2f** (376 MHz, $CDCl_3$)

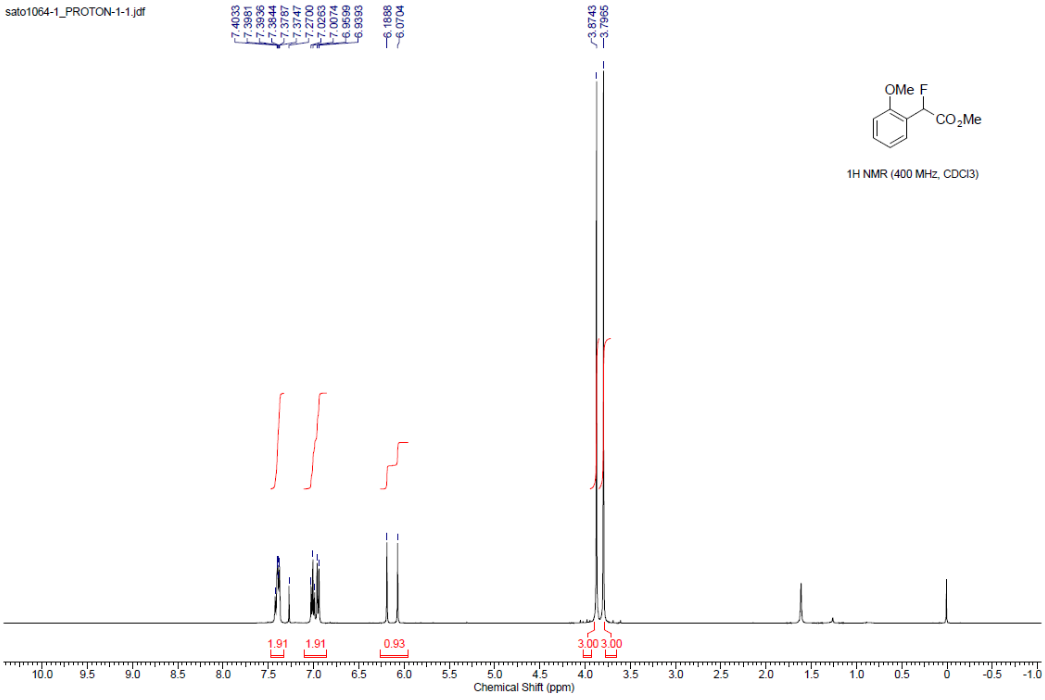

Figure S22. ^{1}H NMR of **2g** (400 MHz, $CDCl_3$)

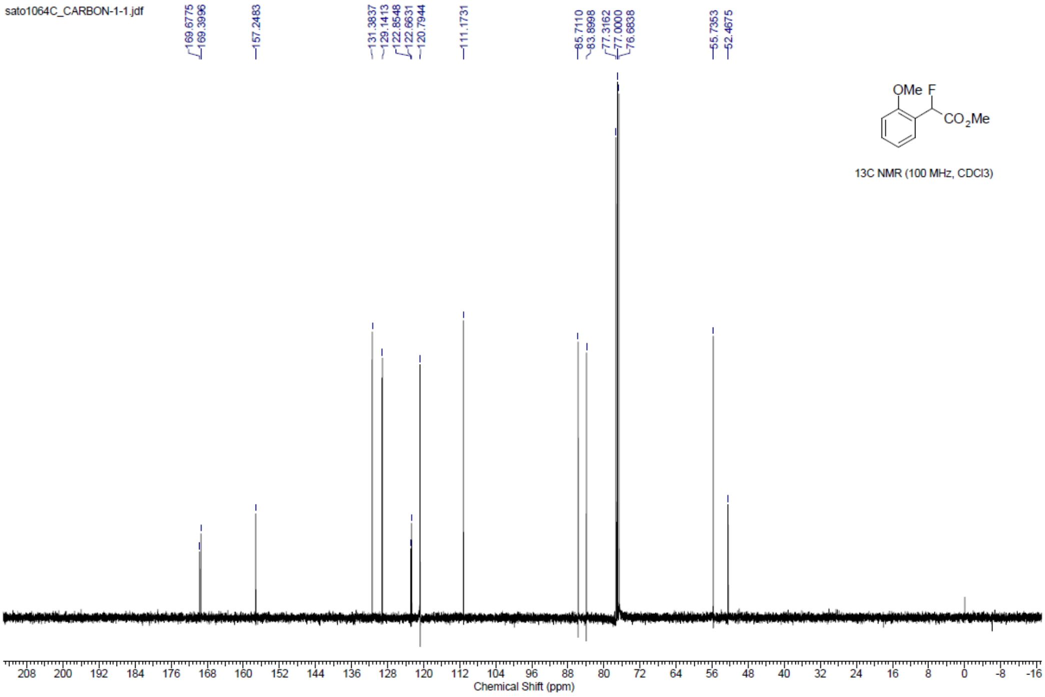

Figure S23. ^{13}C NMR of **2g** (100 MHz, $CDCl_3$)

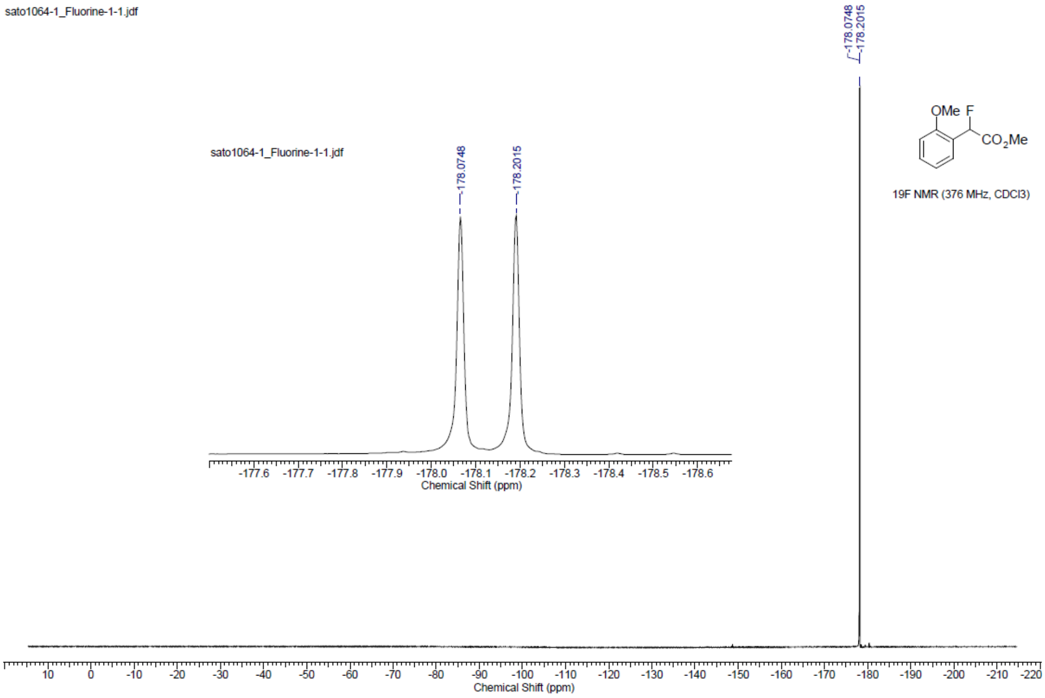

Figure S24. ^{19}F NMR of **2g** (376 MHz, $CDCl_3$)

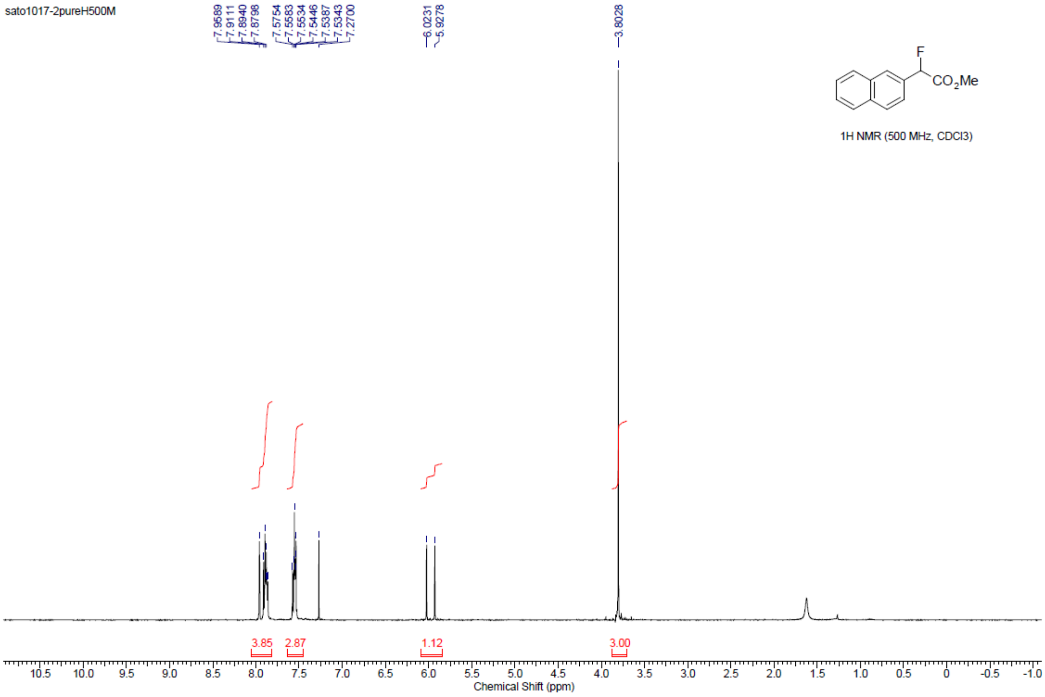

Figure S25. ^{1}H NMR of **2h** (400 MHz, $CDCl_3$)

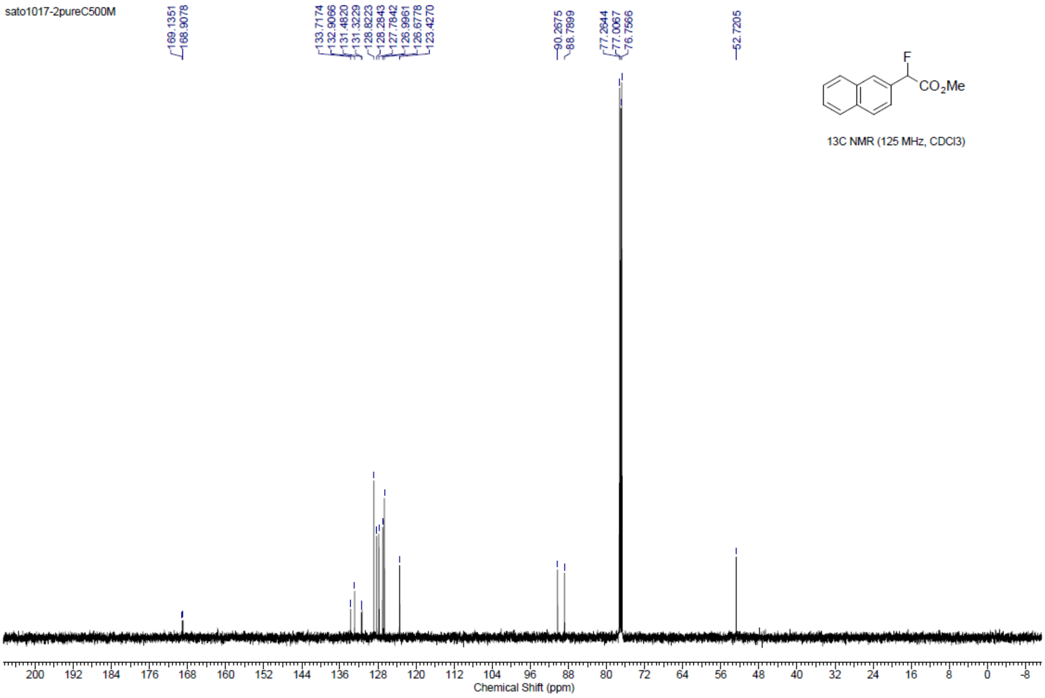

Figure S26. ^{13}C NMR of **2h** (100 MHz, $CDCl_3$)

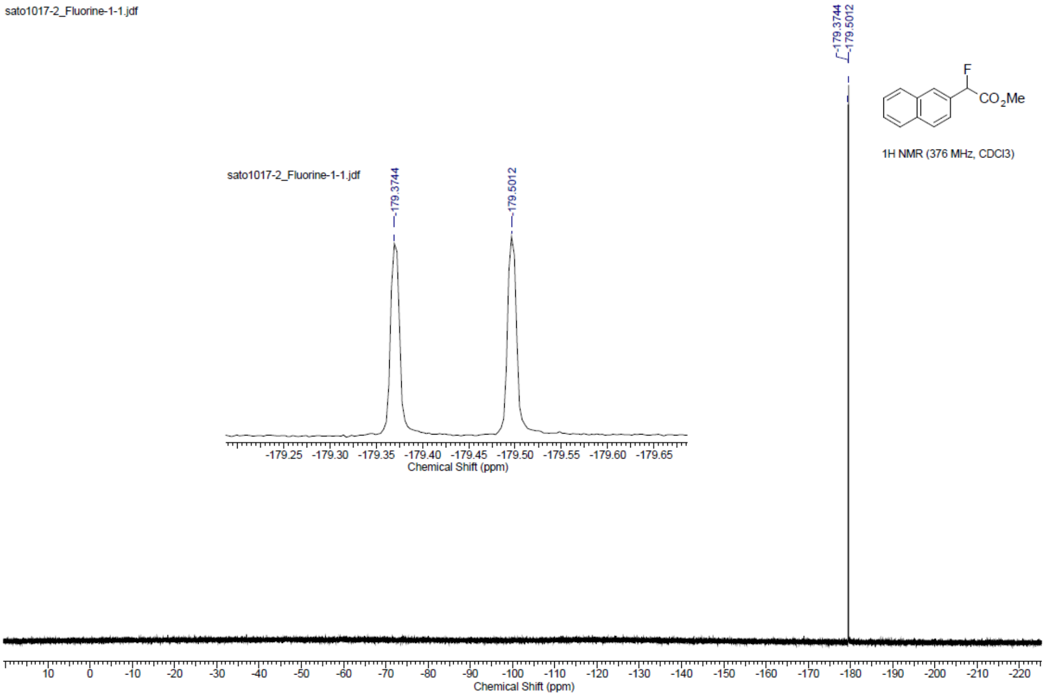

Figure S27. ^{19}F NMR of **2h** (376 MHz, $CDCl_3$)

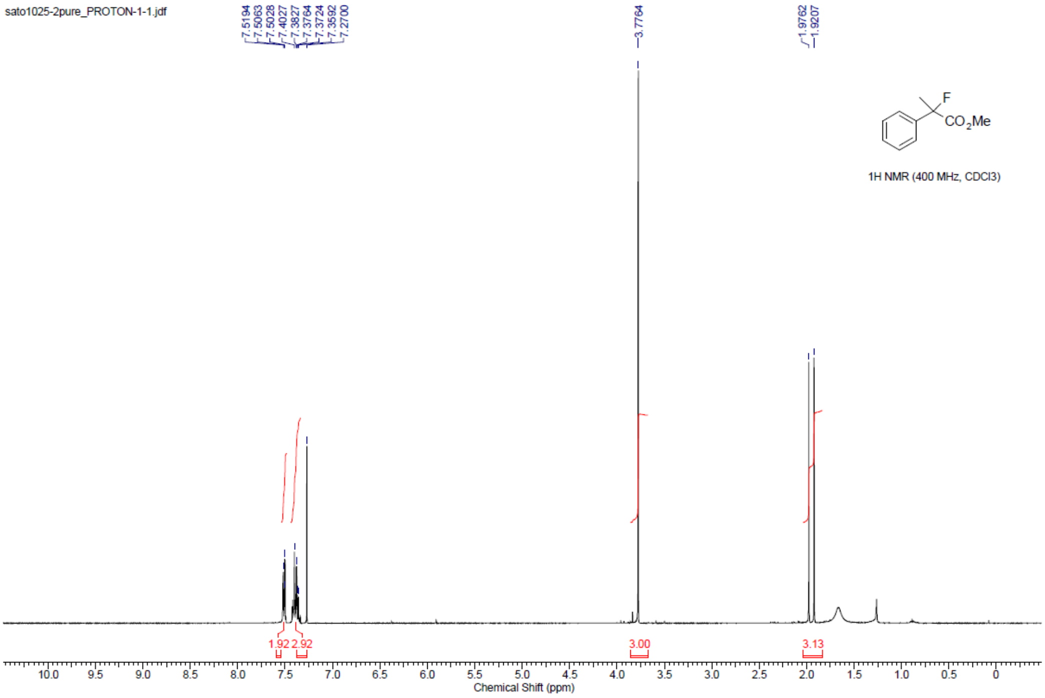

Figure S28. ^{1}H NMR of **2i** (400 MHz, $CDCl_3$)

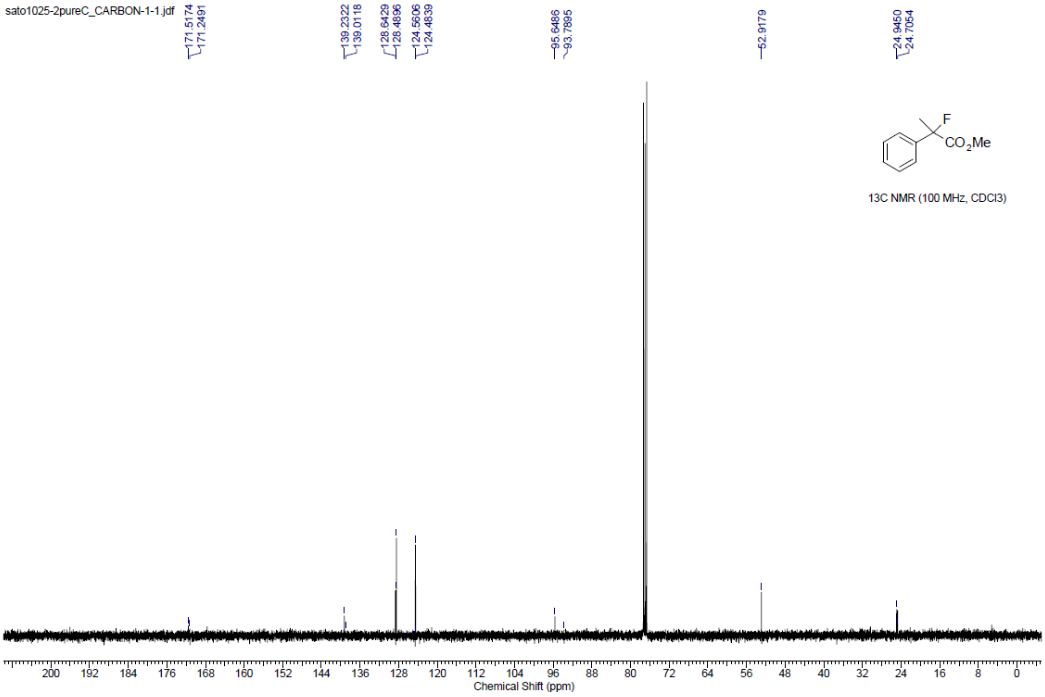

Figure S29. ^{13}C NMR of **2i** (100 MHz, $CDCl_3$)

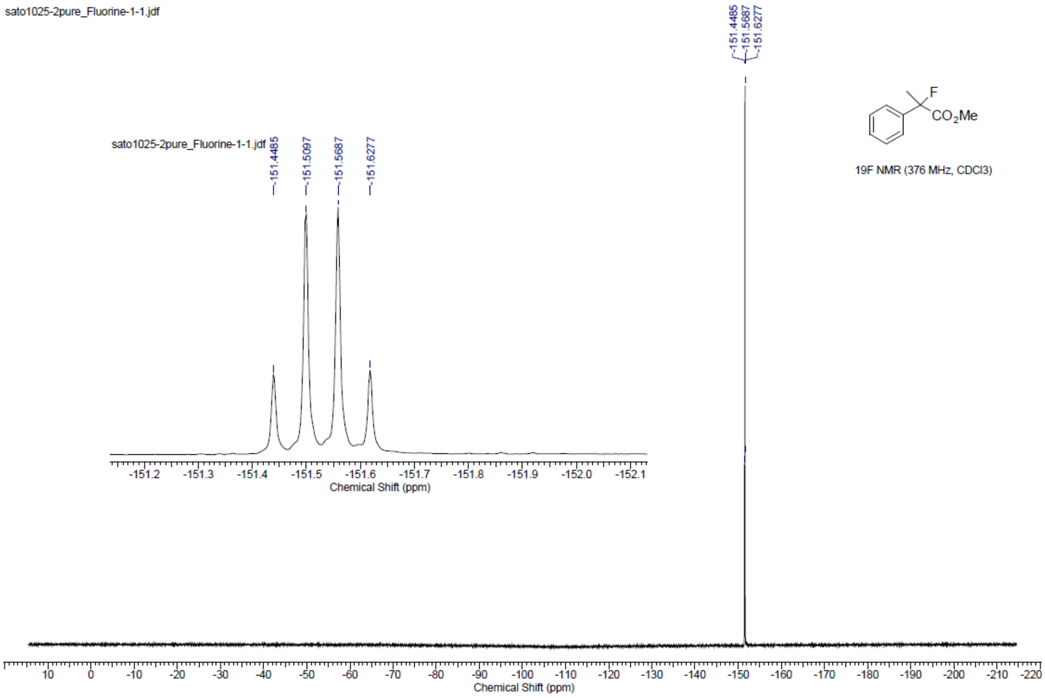

Figure S30. ^{19}F NMR of **2i** (376 MHz, $CDCl_3$)

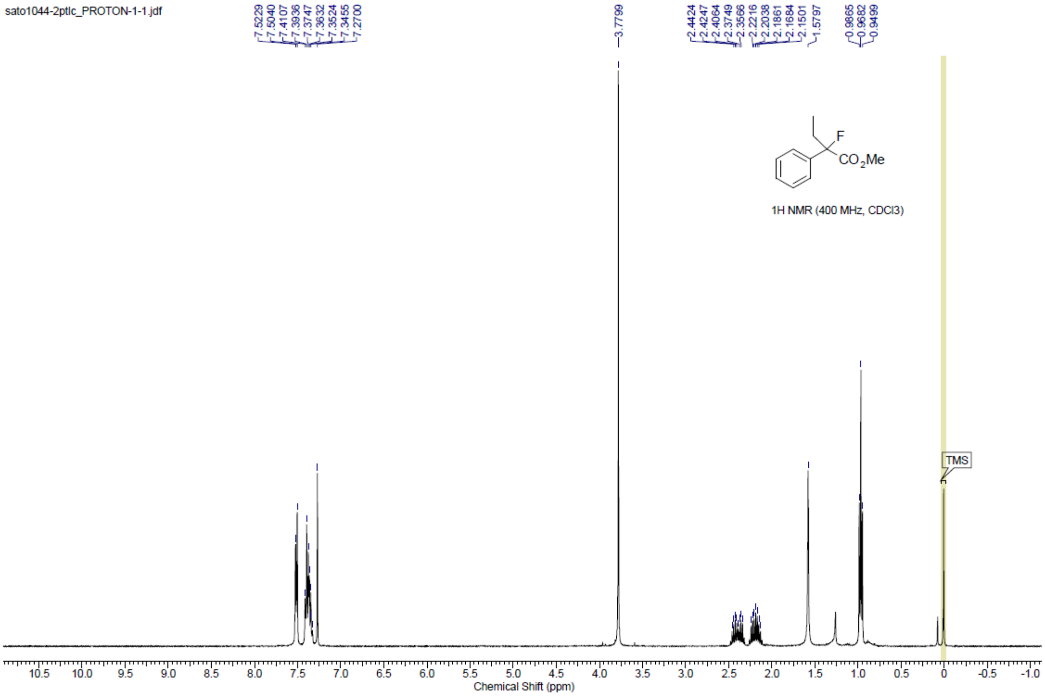

Figure S31. ^{1}H NMR of **2j** (400 MHz, $CDCl_3$)

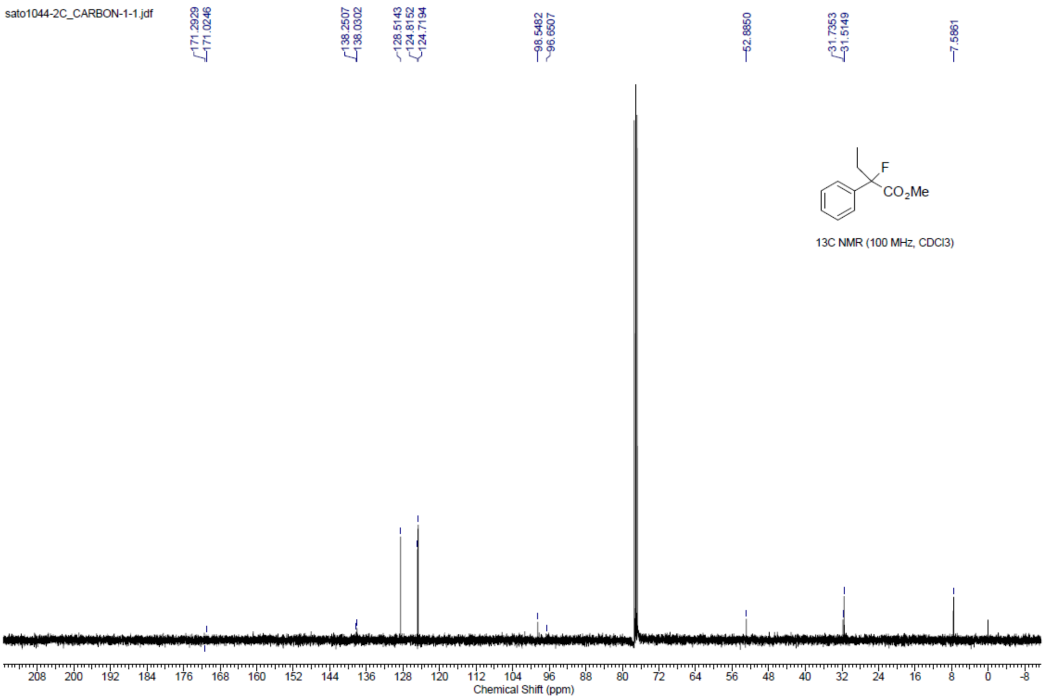

Figure S32. ^{13}C NMR of **2j** (100 MHz, $CDCl_3$)

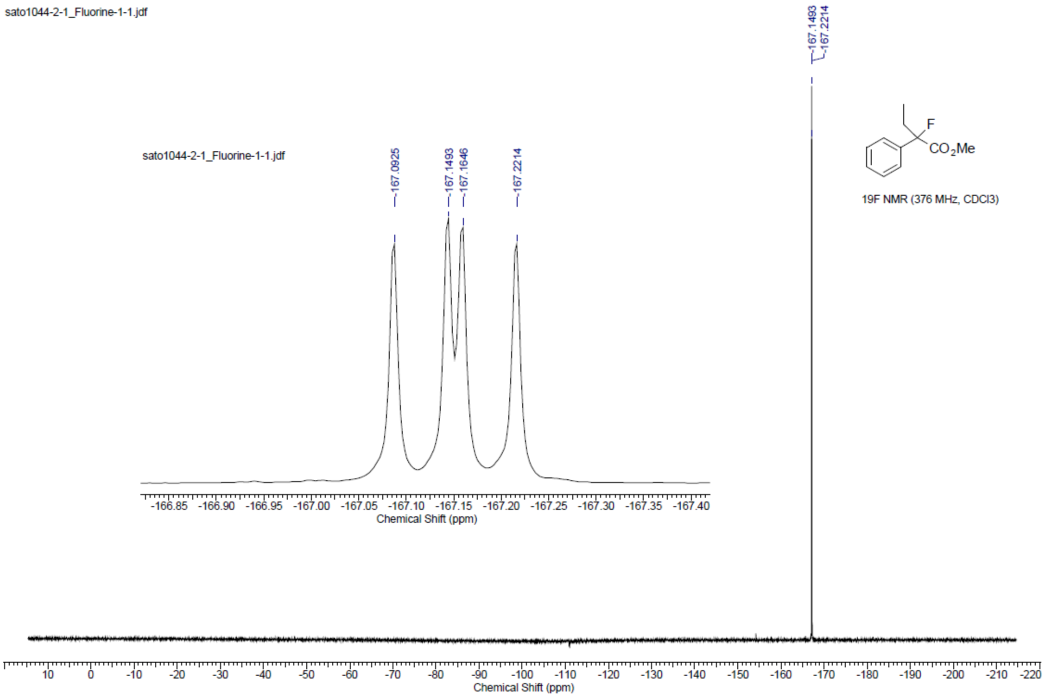

Figure S33. ^{19}F NMR of **2j** (376 MHz, $CDCl_3$)

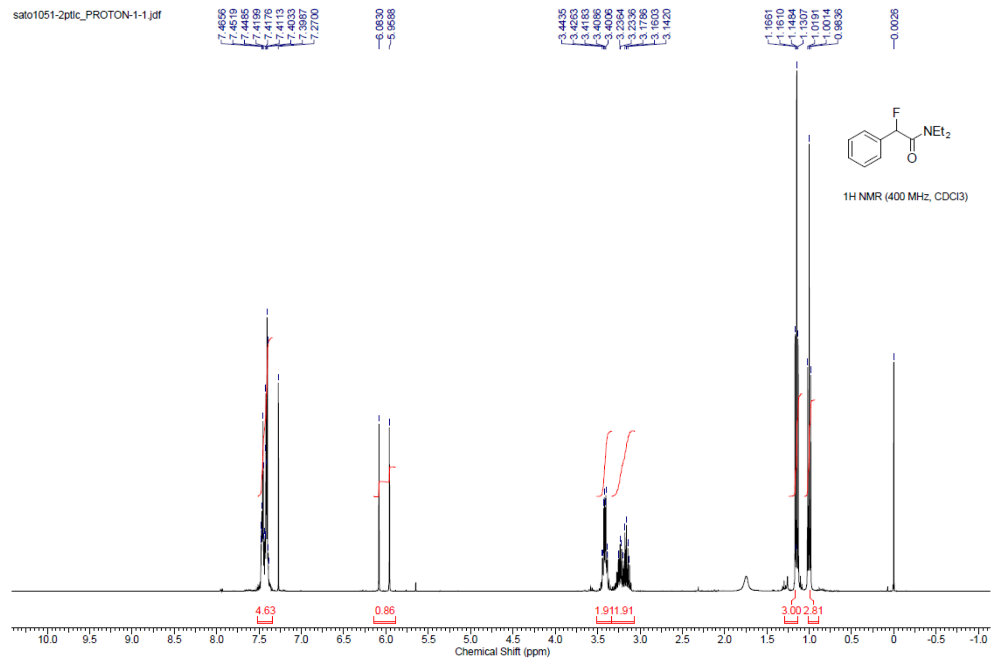

Figure S34. ^{1}H NMR of **2n** (400 MHz, $CDCl_3$)

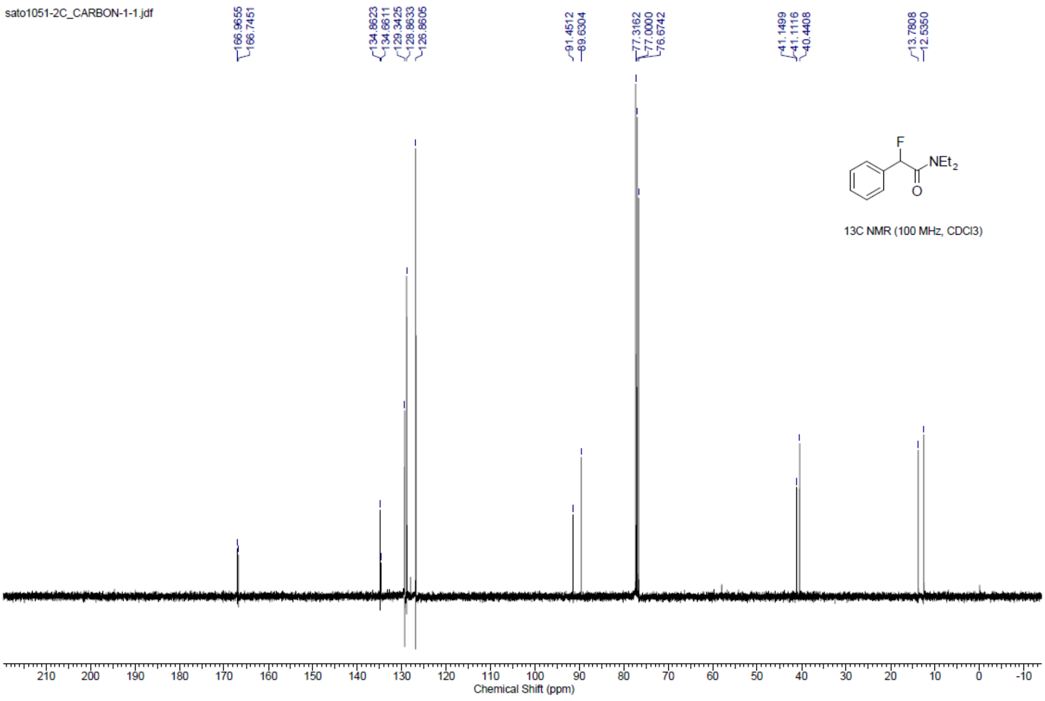

Figure S35. ^{13}C NMR of **2n** (100 MHz, $CDCl_3$)

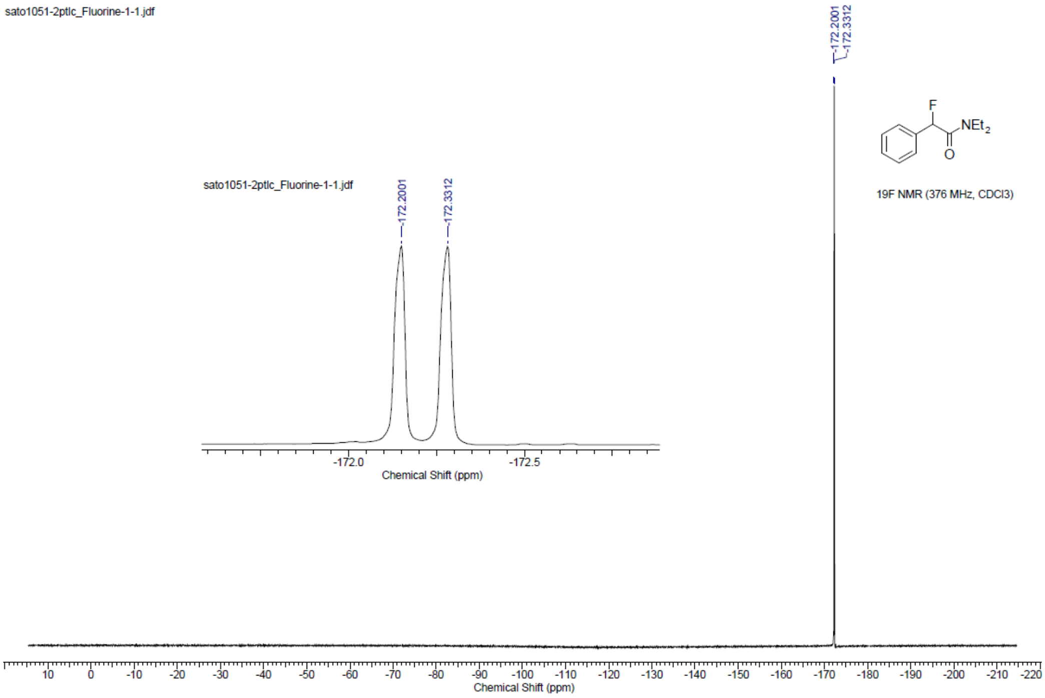

Figure S36. ^{19}F NMR of **2n** (376 MHz, $CDCl_3$)

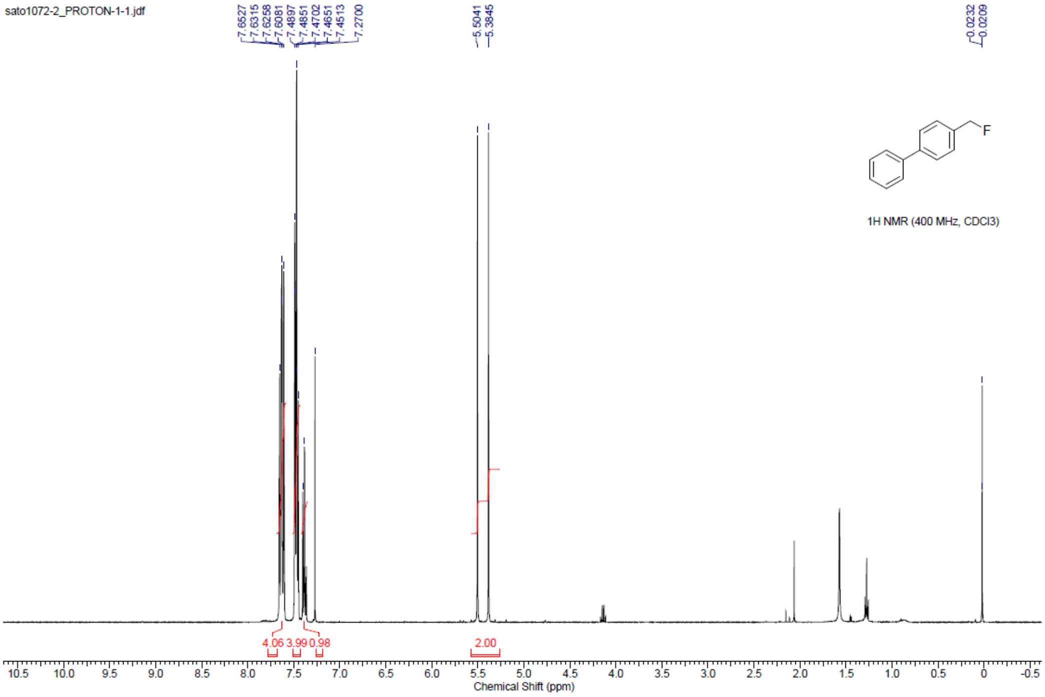

Figure S37. ^{1}H NMR of **2o** (400 MHz, $CDCl_3$)

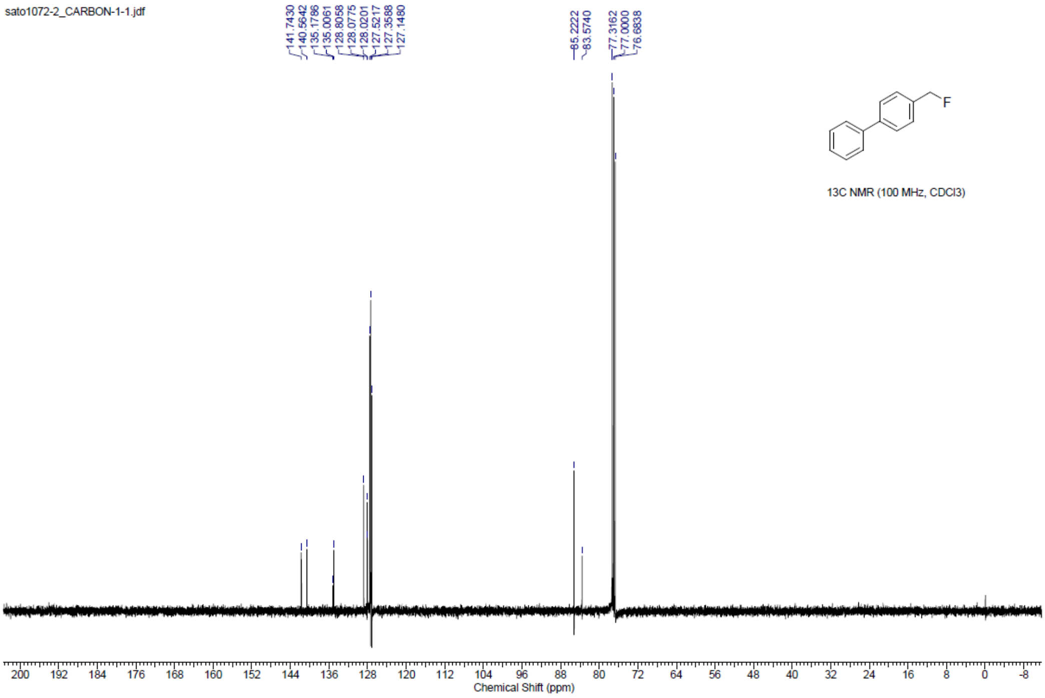

Figure S38. ^{13}C NMR of **2o** (100 MHz, $CDCl_3$)

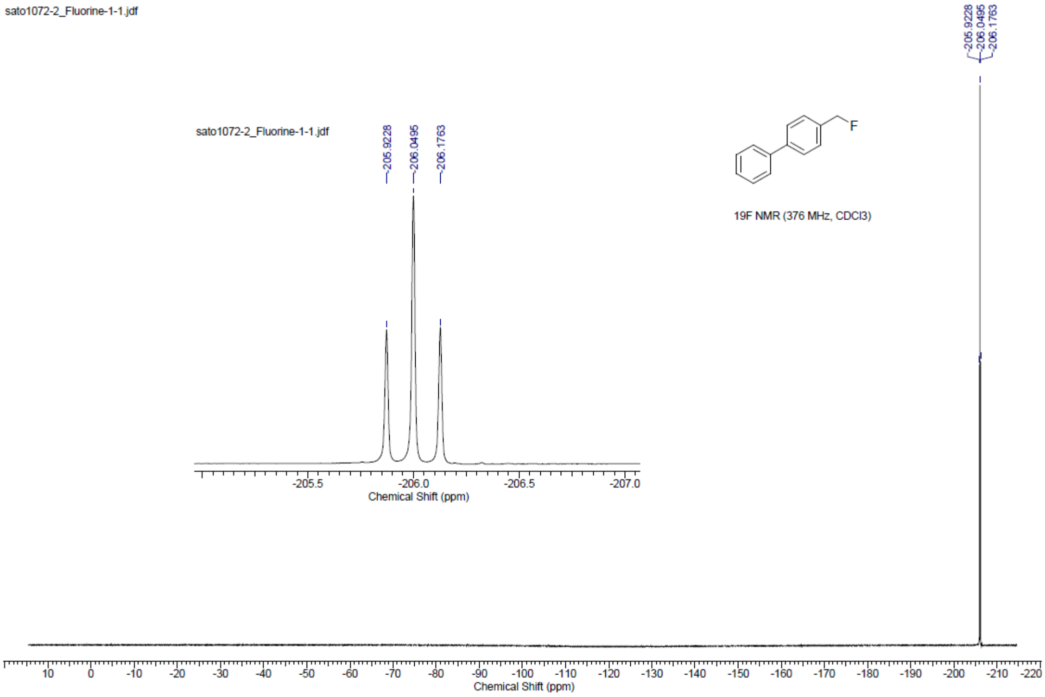

Figure S39. ^{19}F NMR of **2o** (376 MHz, $CDCl_3$)

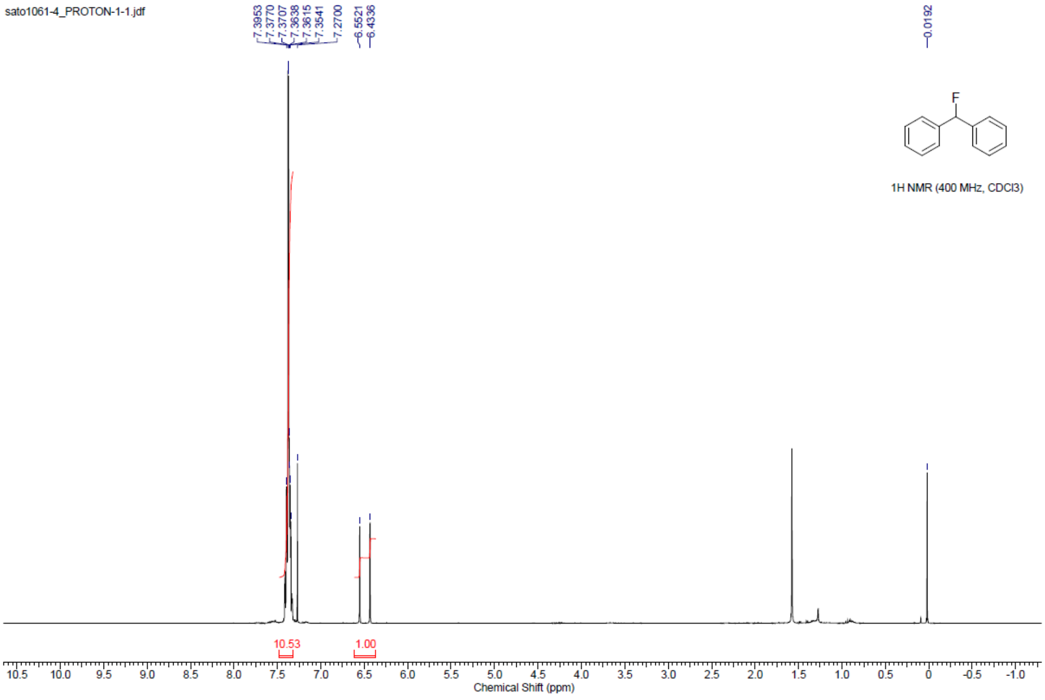

Figure S40. ¹H NMR of **2q** (400 MHz, $CDCl_3$)

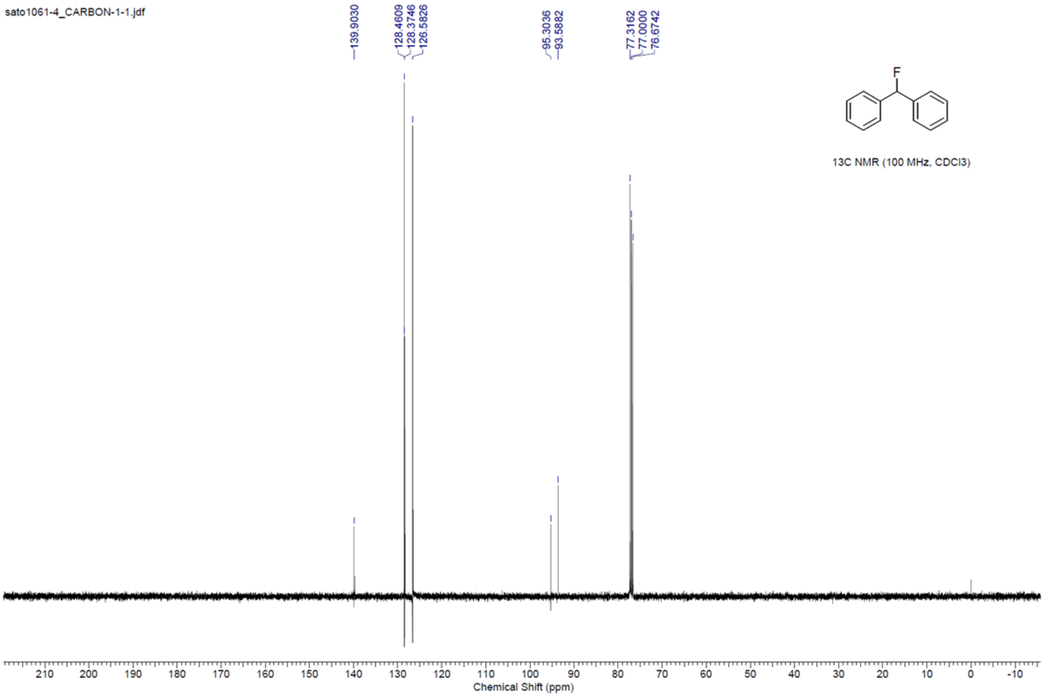

Figure S41. ¹³C NMR of **2q** (100 MHz, $CDCl_3$)

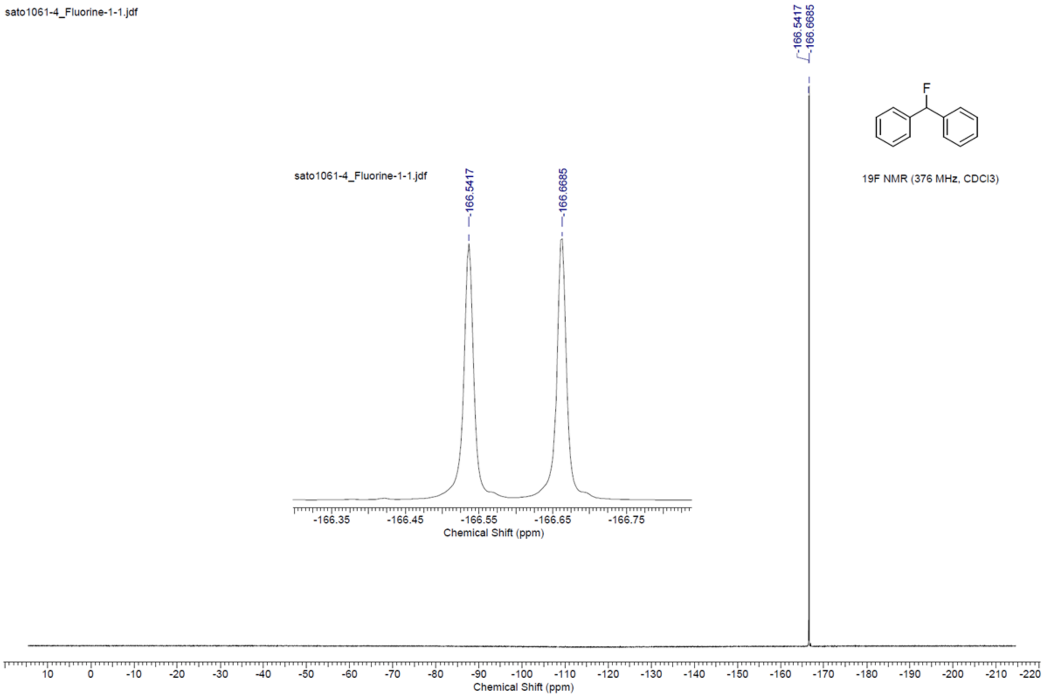

Figure S42. ^{19}F NMR of **2q** (376 MHz, $CDCl_3$)

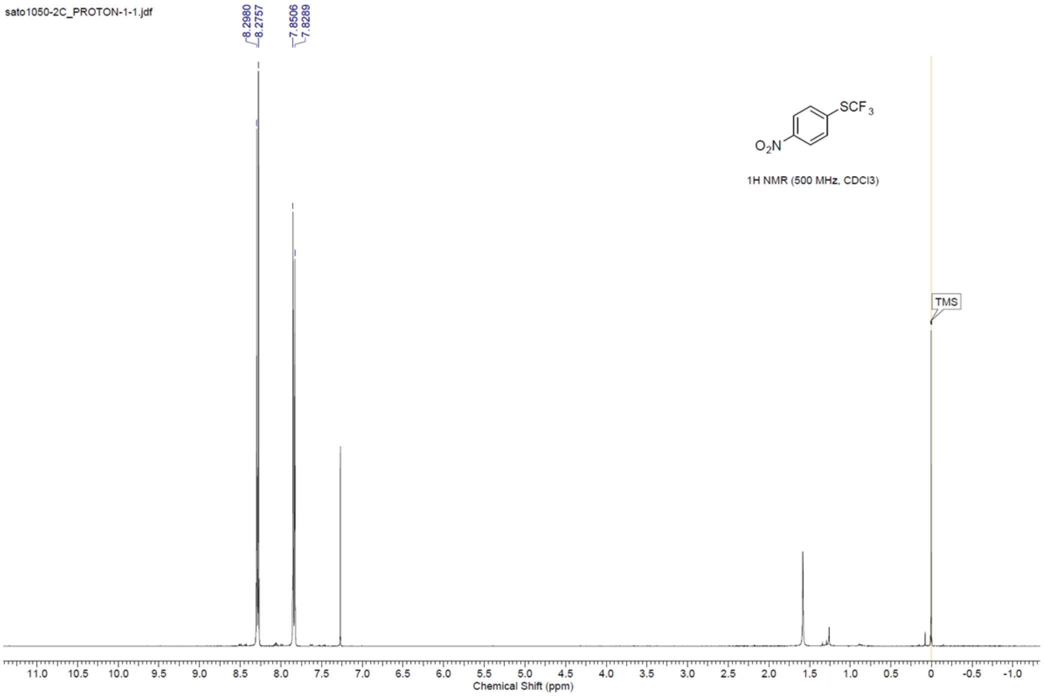

Figure S43. ^{1}H NMR of **5a** (400 MHz, $CDCl_3$)

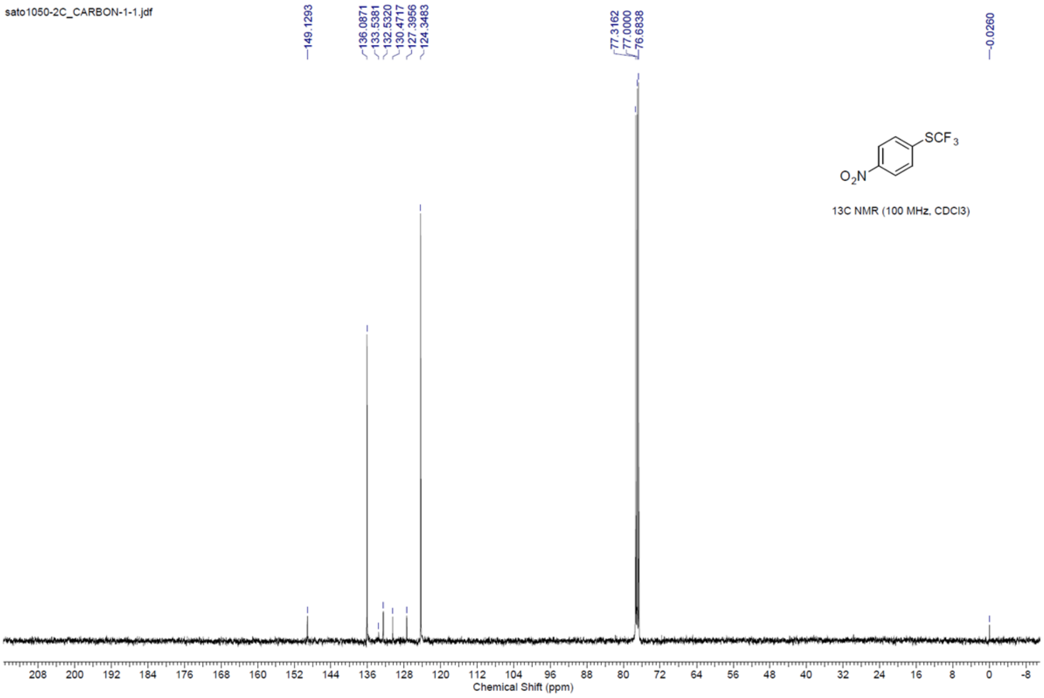

Figure S44. ^{13}C NMR of **5a** (100 MHz, $CDCl_3$)

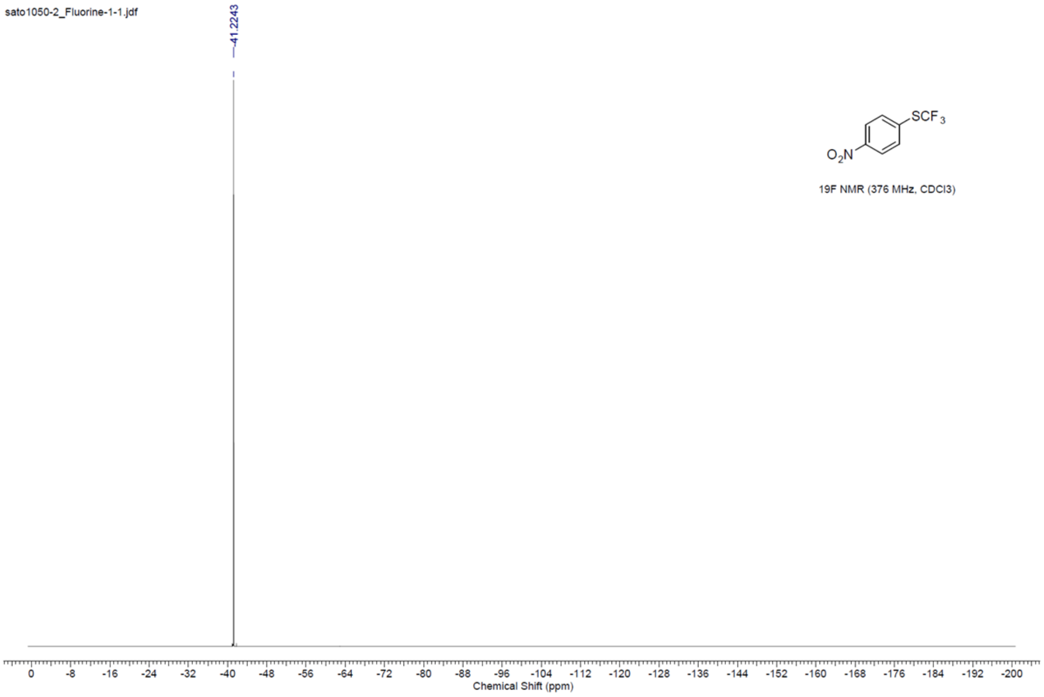

Figure S45. ^{19}F NMR of **5a** (376 MHz, $CDCl_3$)

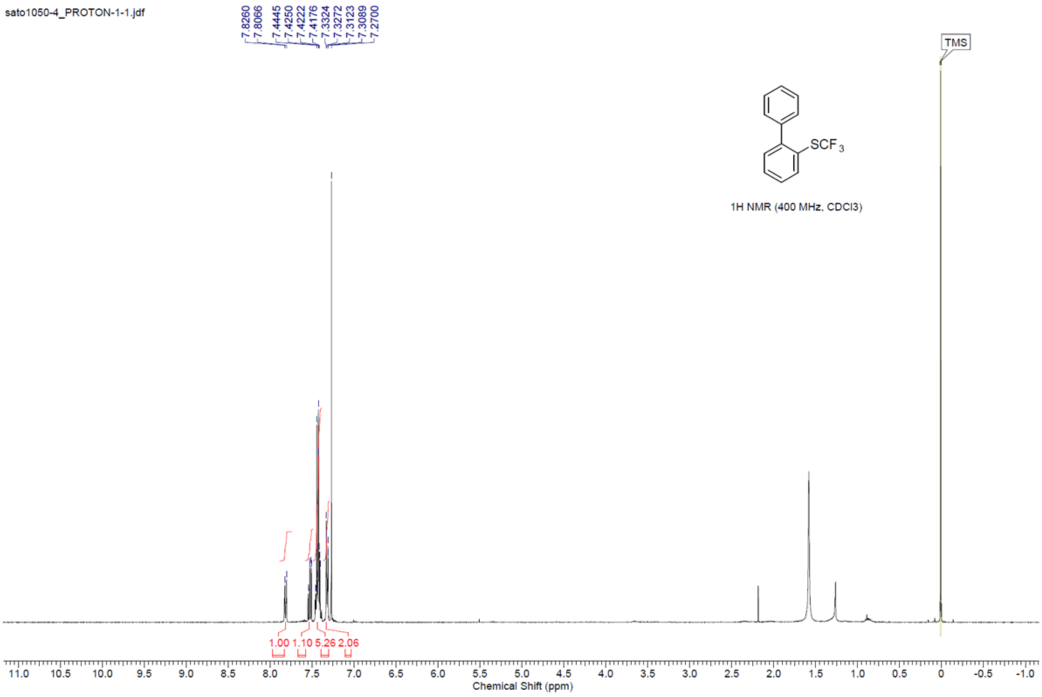

Figure S46. ^{1}H NMR of **5b** (400 MHz, $CDCl_3$)

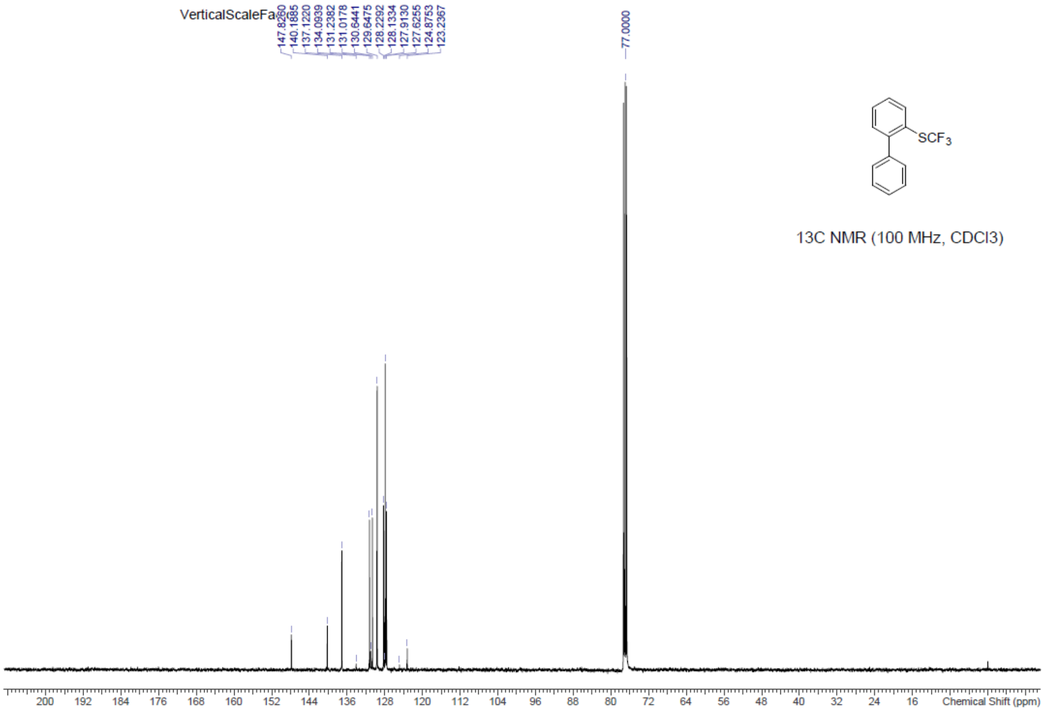

Figure S47. ^{13}C NMR of **5b** (100 MHz, $CDCl_3$)

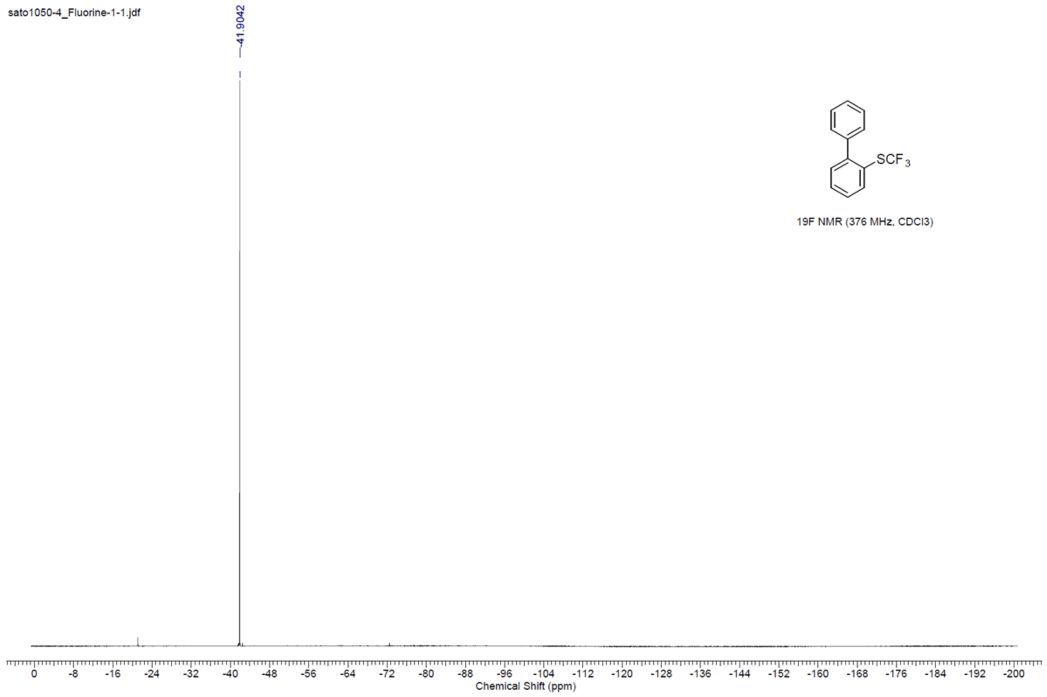

Figure S48. ^{19}F NMR of **5b** (376 MHz, $CDCl_3$)

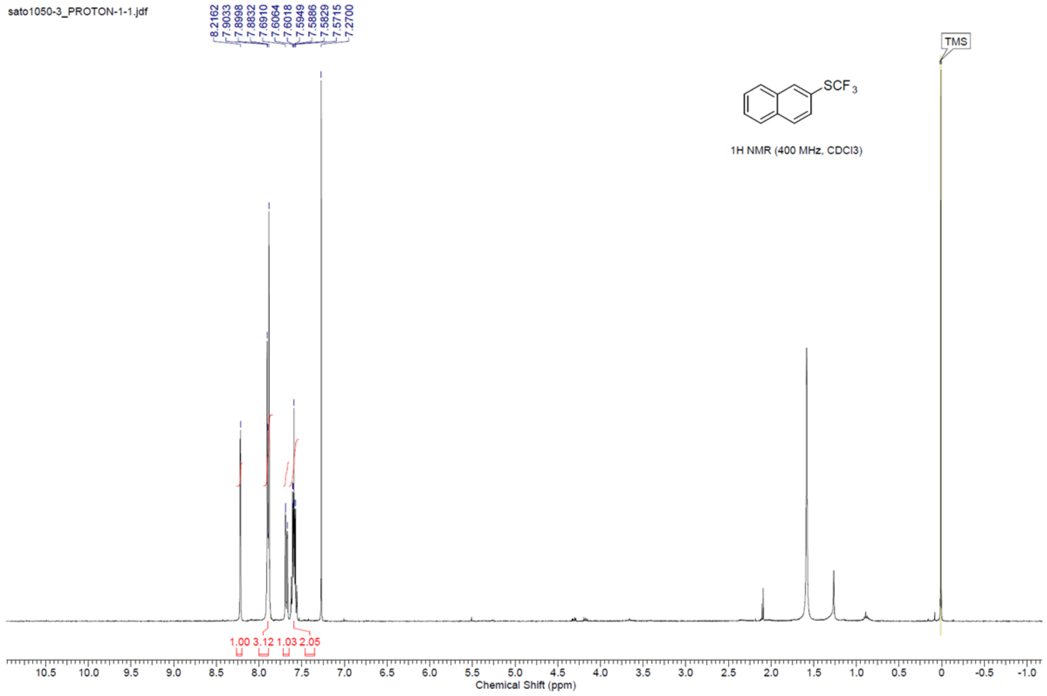

Figure S49. ^{1}H NMR of **5c** (400 MHz, $CDCl_3$)

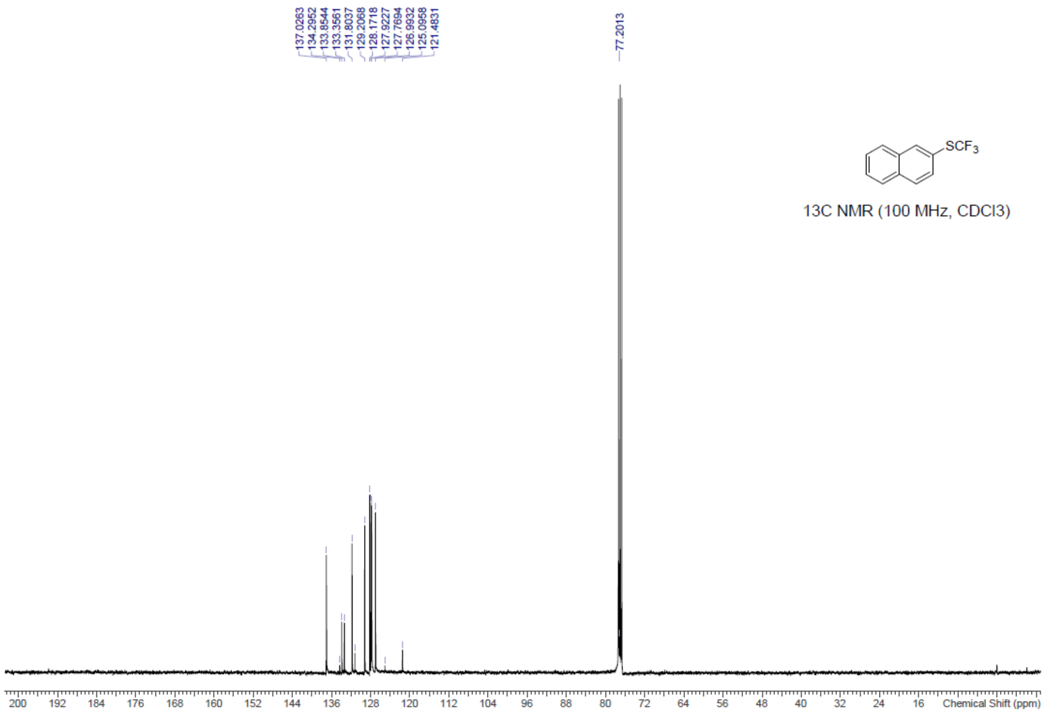

Figure S50. ^{13}C NMR of **5c** (100 MHz, $CDCl_3$)

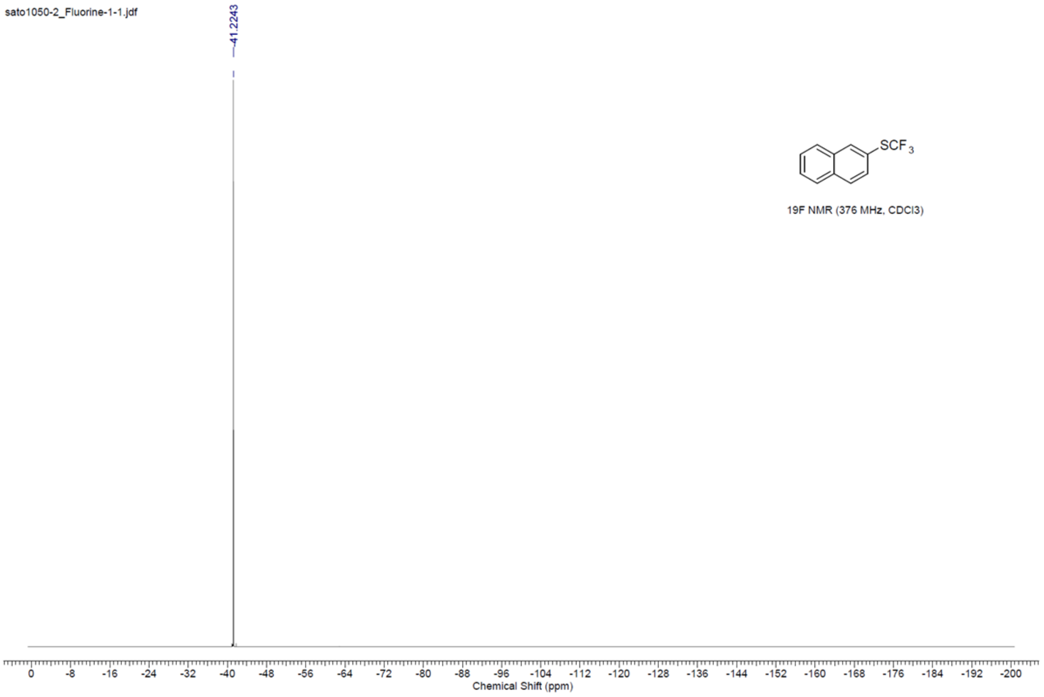

Figure S51. ^{19}F NMR of **5a** (376 MHz, $CDCl_3$)

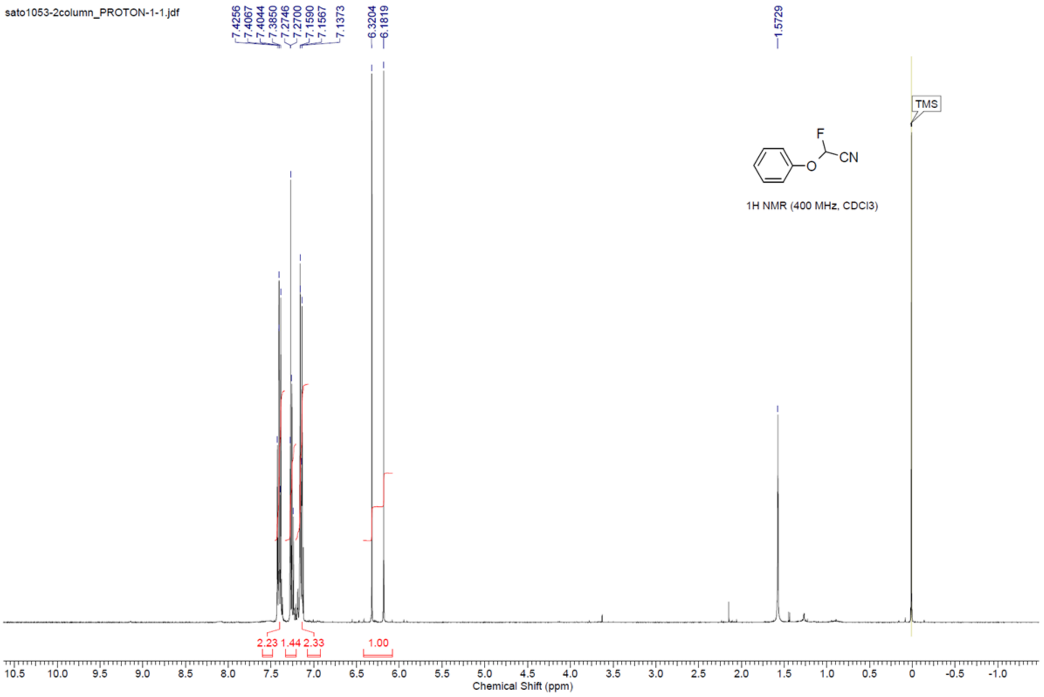

Figure S52. ^{1}H NMR of **6a** (400 MHz, $CDCl_3$)

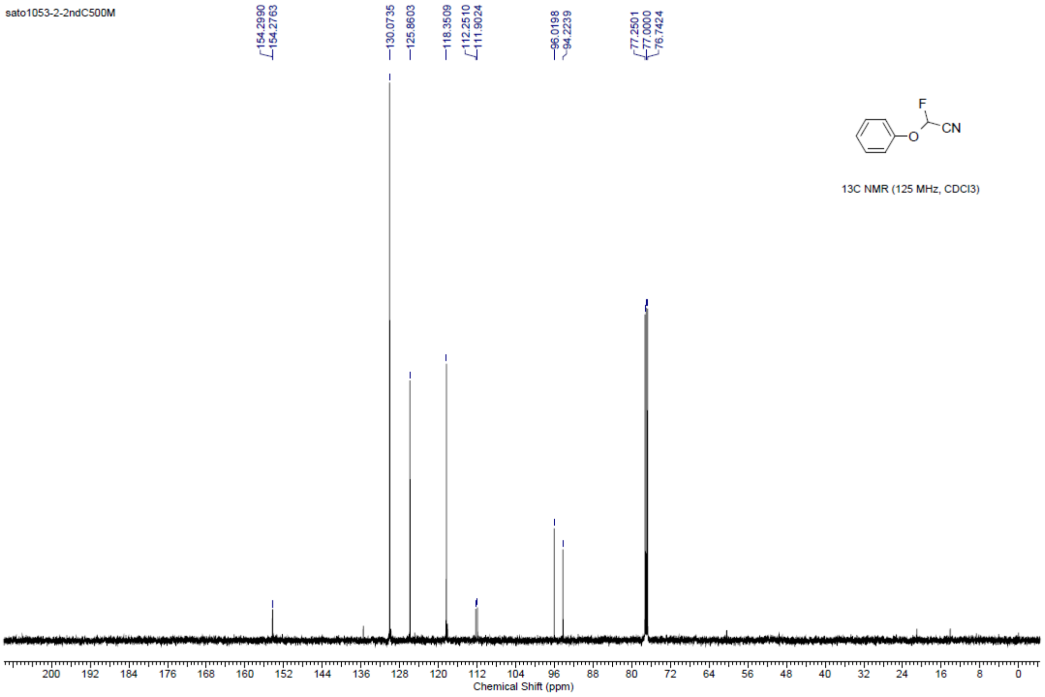

Figure S53. ^{13}C NMR of **6a** (125 MHz, $CDCl_3$)

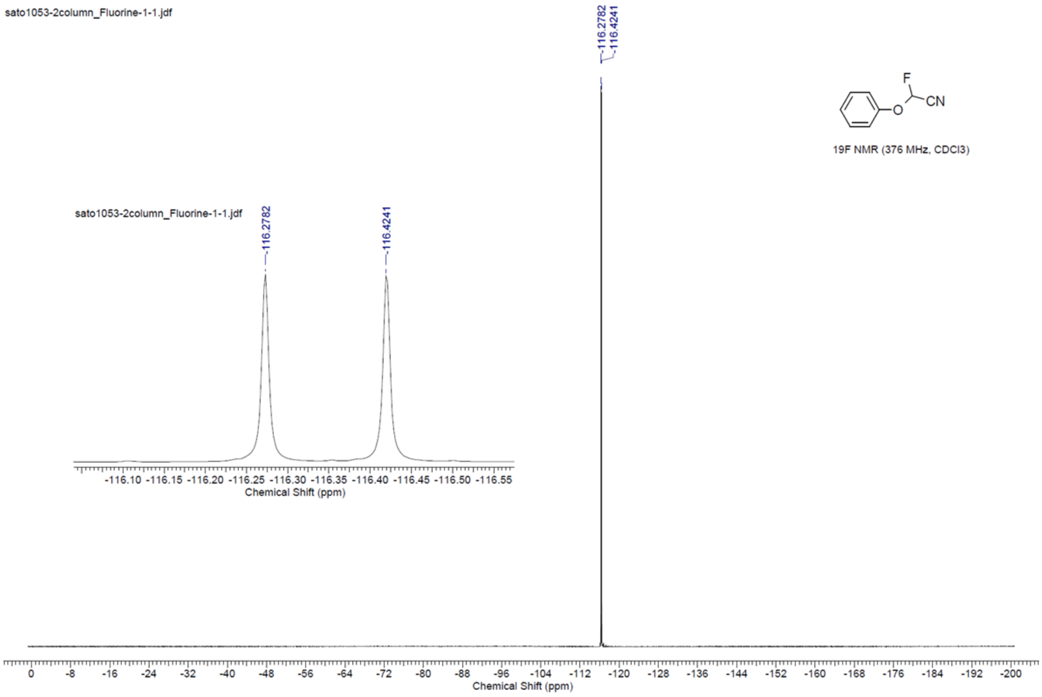

Figure S54. ^{19}F NMR of **6a** (376 MHz, $CDCl_3$)

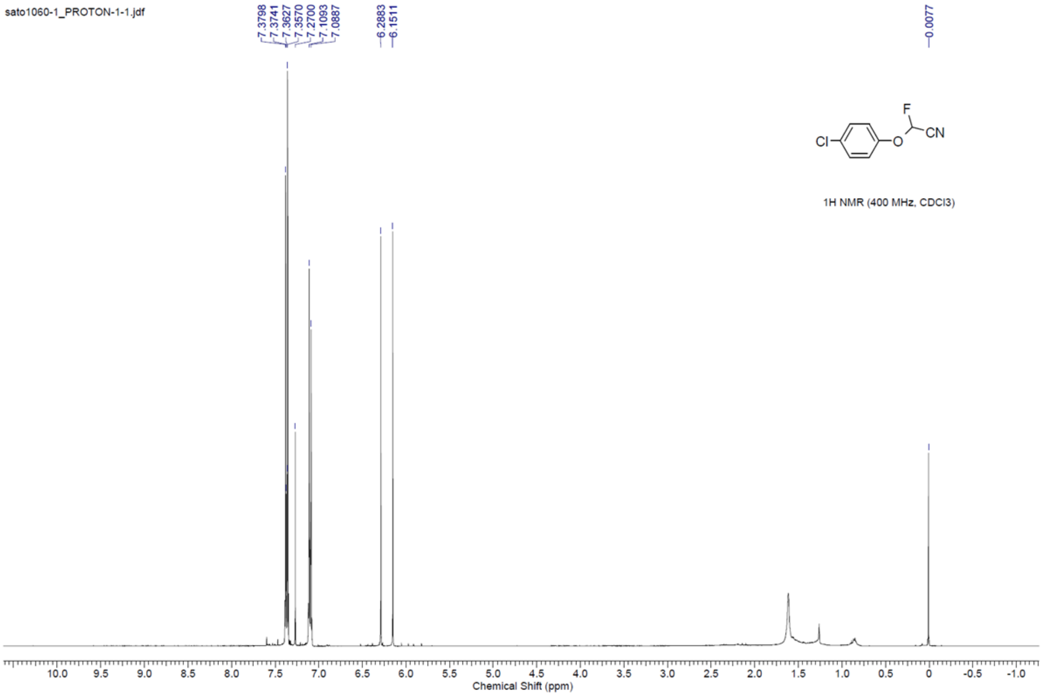

Figure S55. ^{1}H NMR of **6b** (400 MHz, $CDCl_3$)

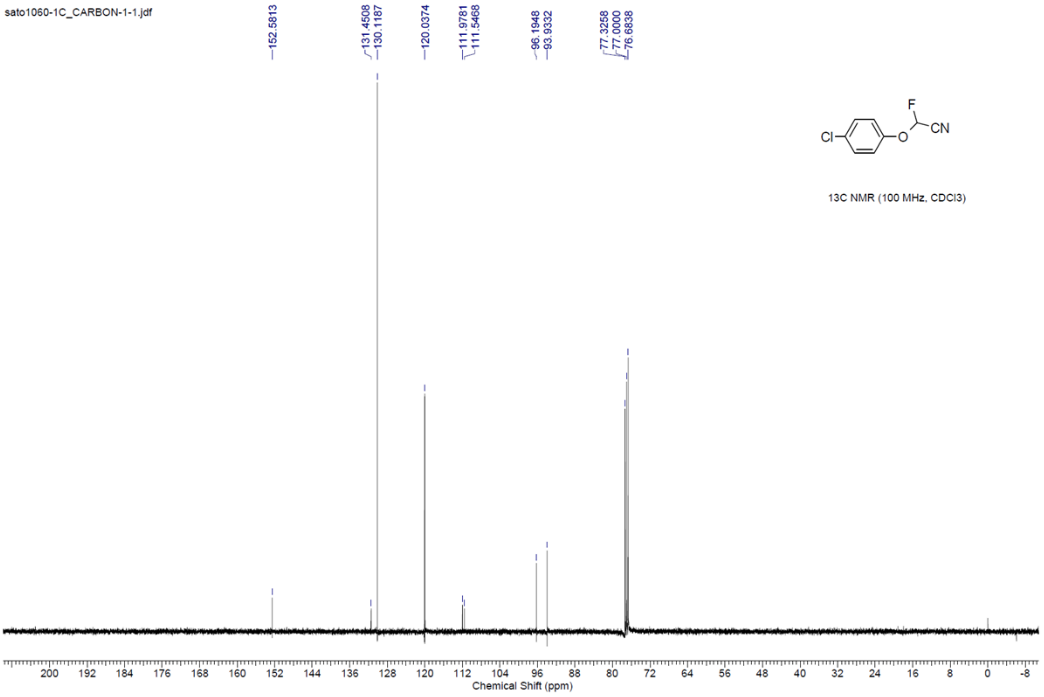

Figure S56. ^{13}C NMR of **6b** (100 MHz, $CDCl_3$)

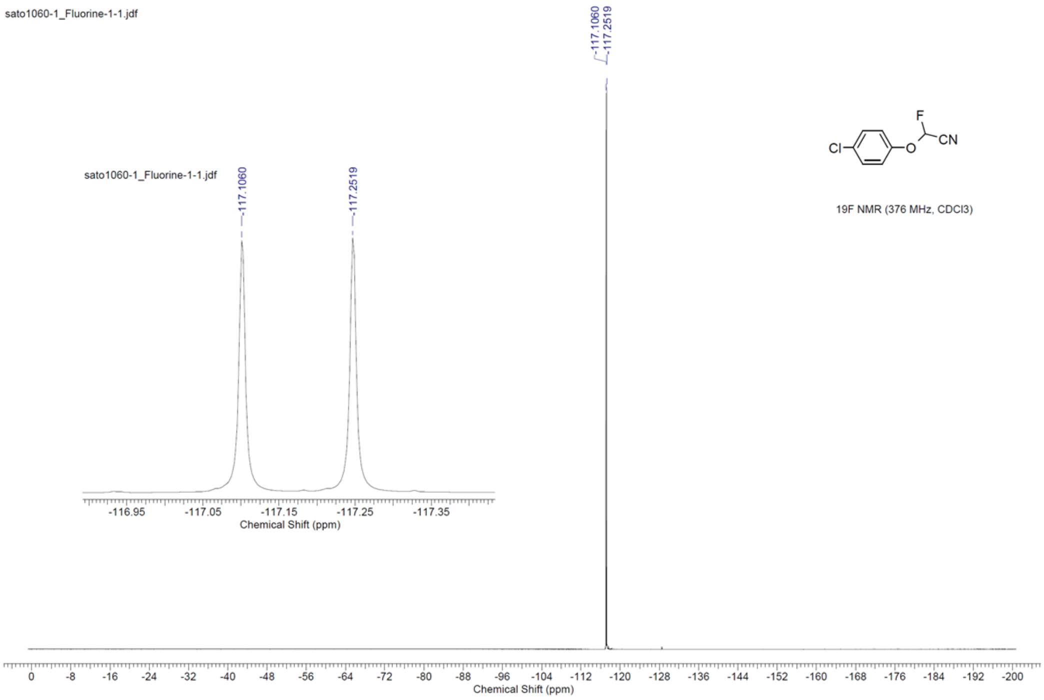

Figure S57. ^{19}F NMR of **6b** (376 MHz, $CDCl_3$)

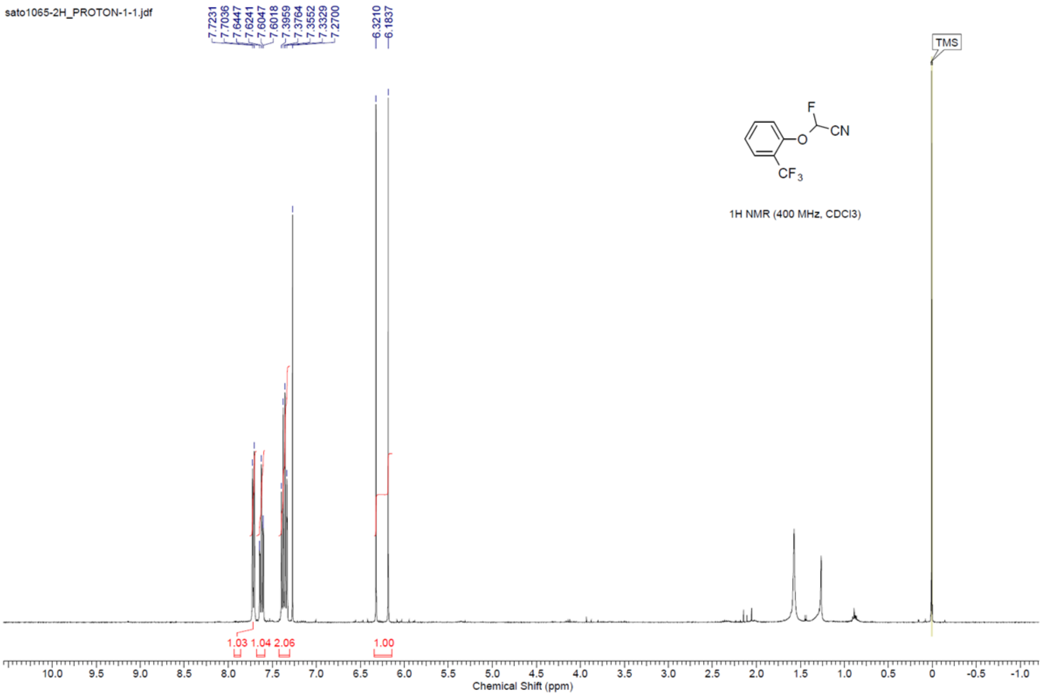

Figure S58. ^{1}H NMR of **6c** (400 MHz, $CDCl_3$)

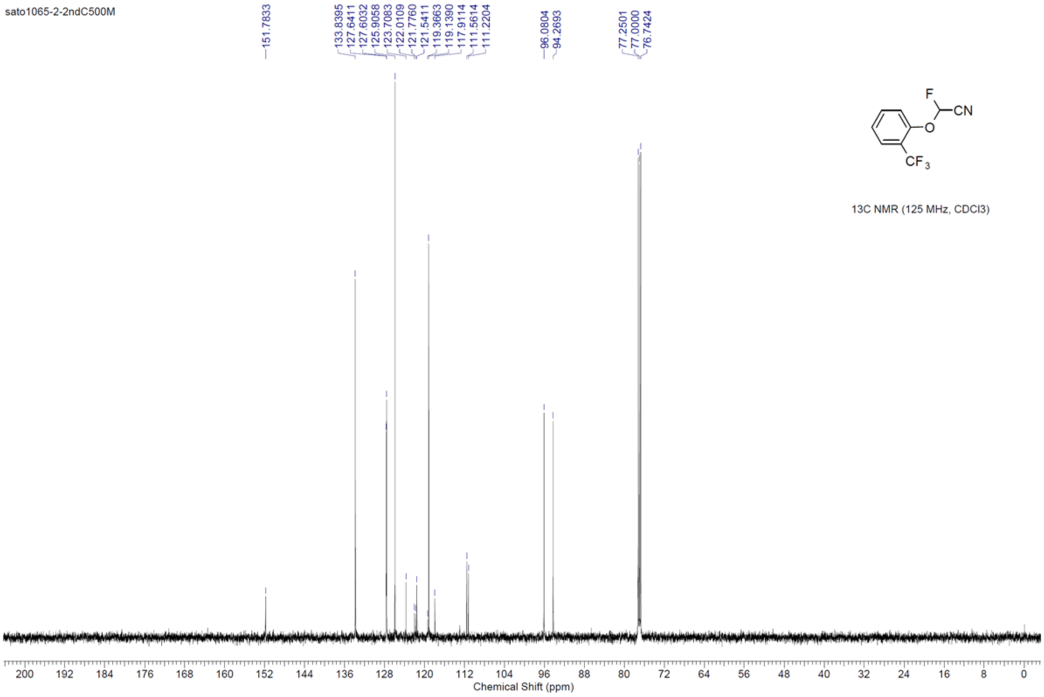

Figure S59. ^{13}C NMR of **6c** (125 MHz, $CDCl_3$)

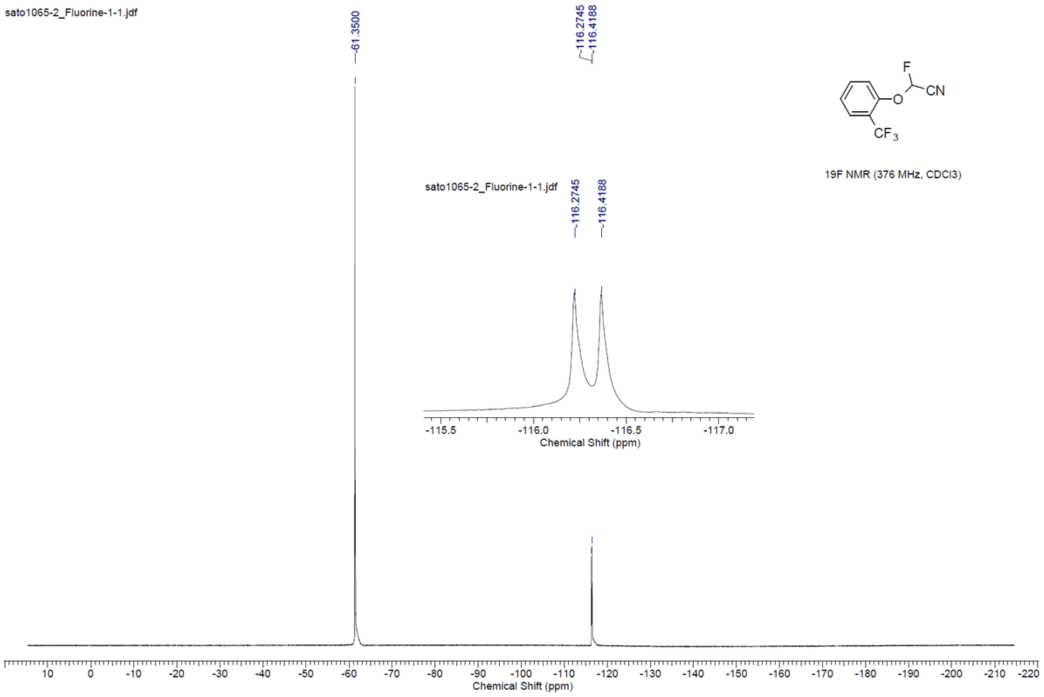

Figure S60. ^{19}F NMR of **6c** (376 MHz, $CDCl_3$)

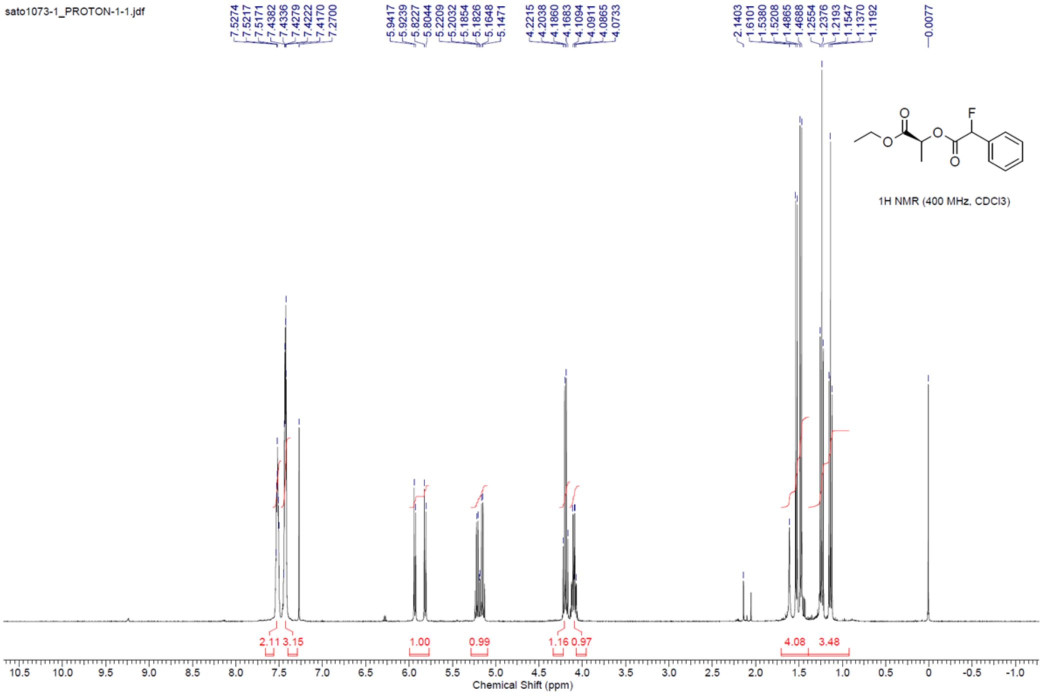

Figure S61. ^{1}H NMR of **8** (400 MHz, $CDCl_3$)

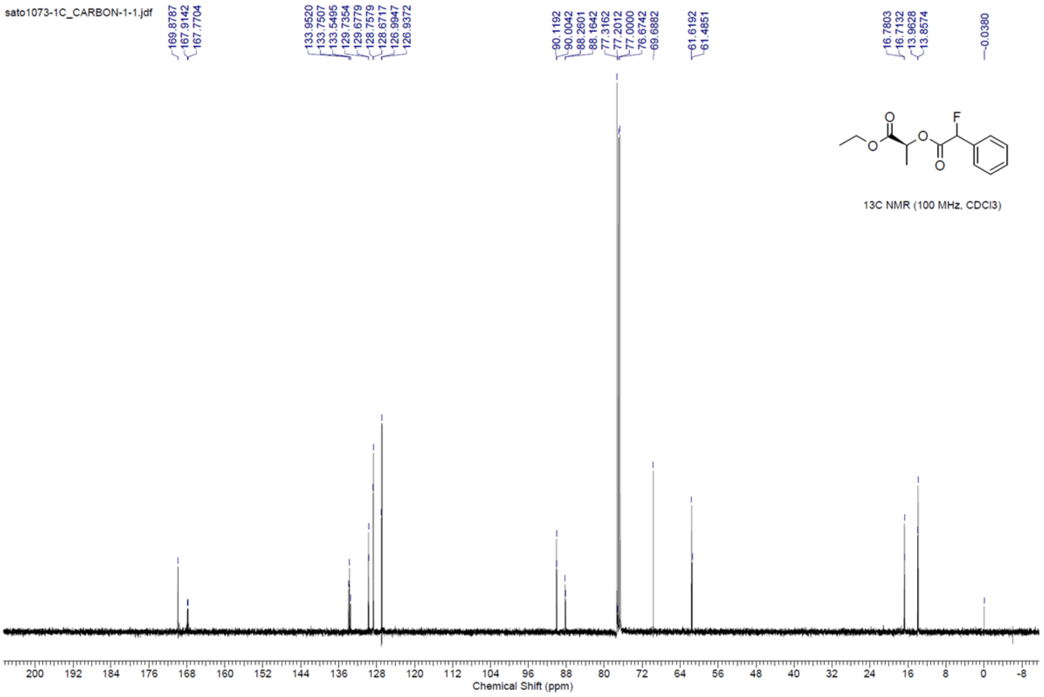

Figure S62. ^{13}C NMR of **8** (100 MHz, $CDCl_3$)

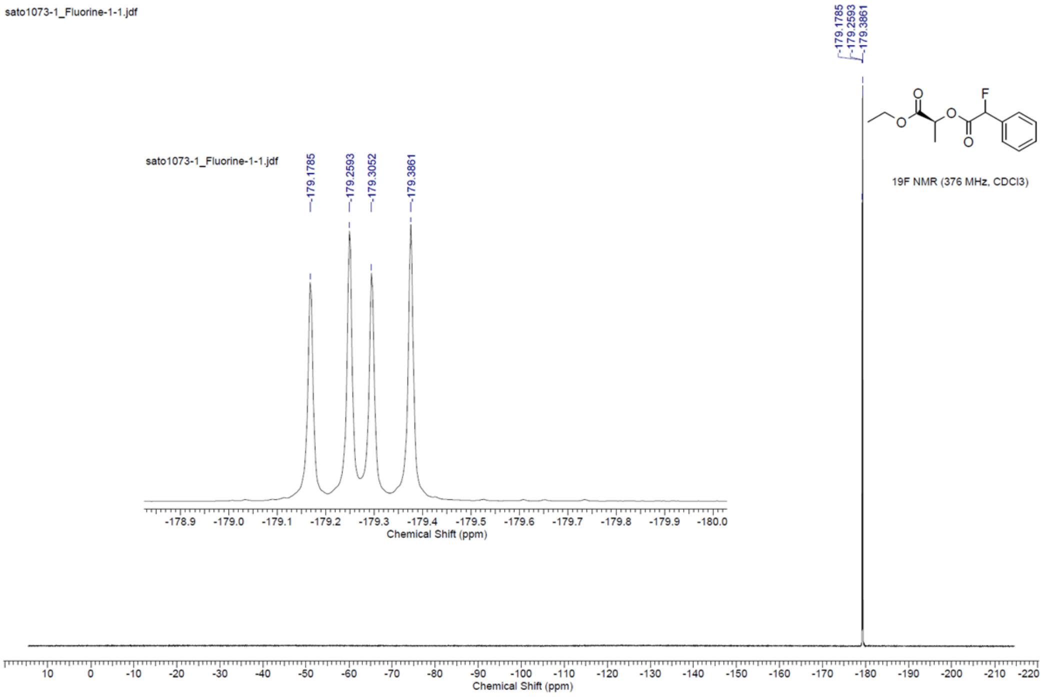

Figure S63. ^{19}F NMR of **8** (376 MHz, $CDCl_3$)

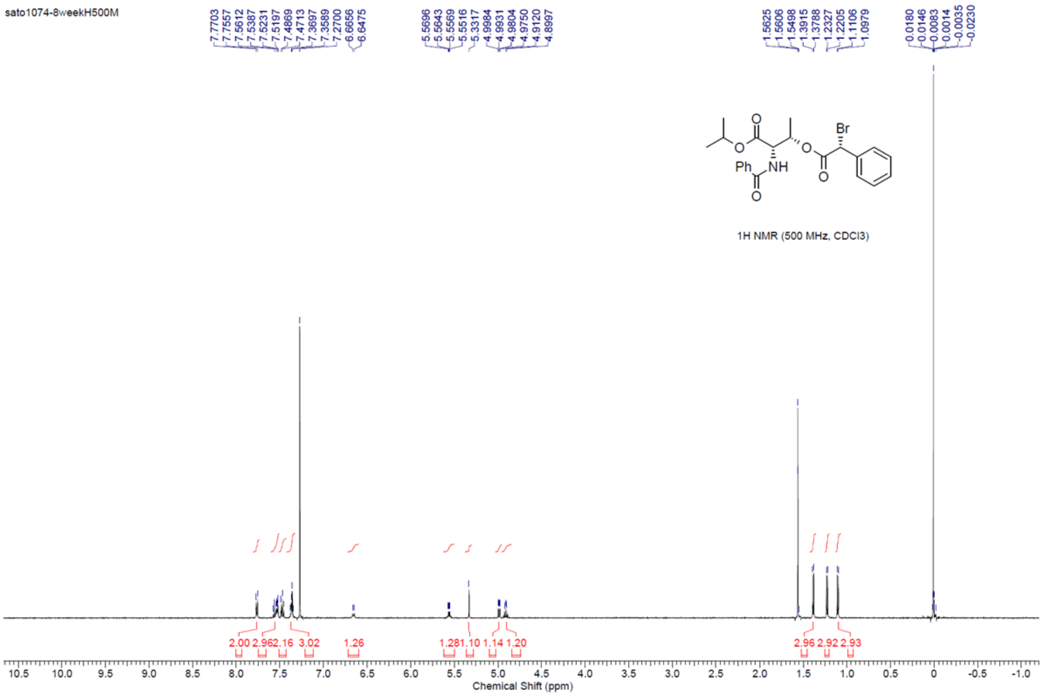

Figure S64. ^{1}H NMR of (αR)-**9** (500 MHz, $CDCl_3$)

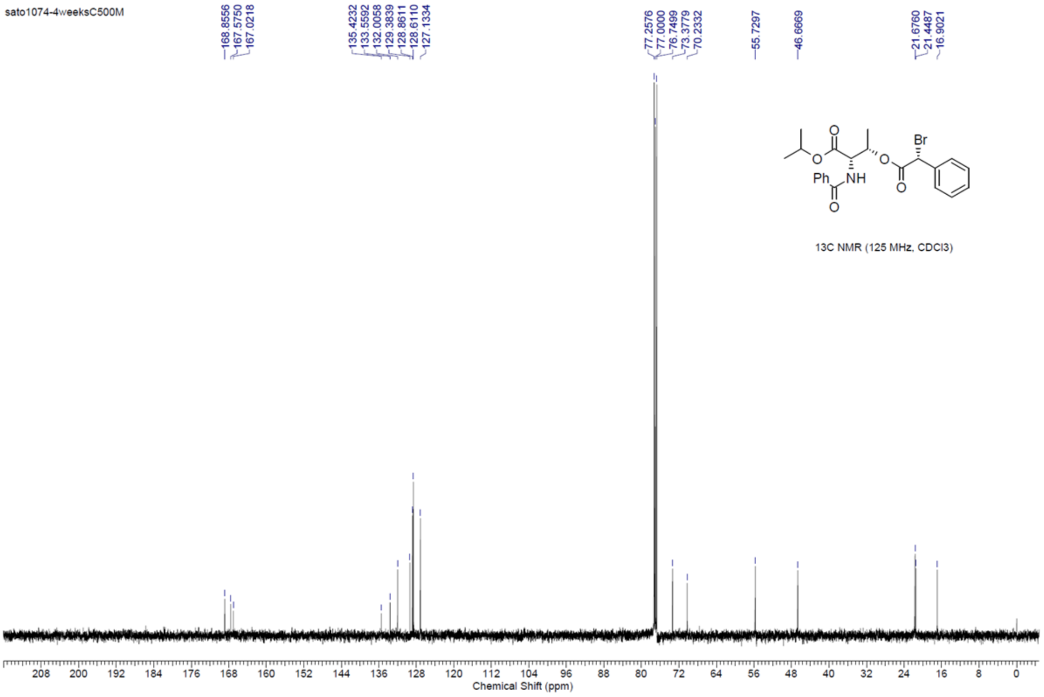

Figure S65. ^{13}C NMR of (αR)-**9** (125 MHz, $CDCl_3$)

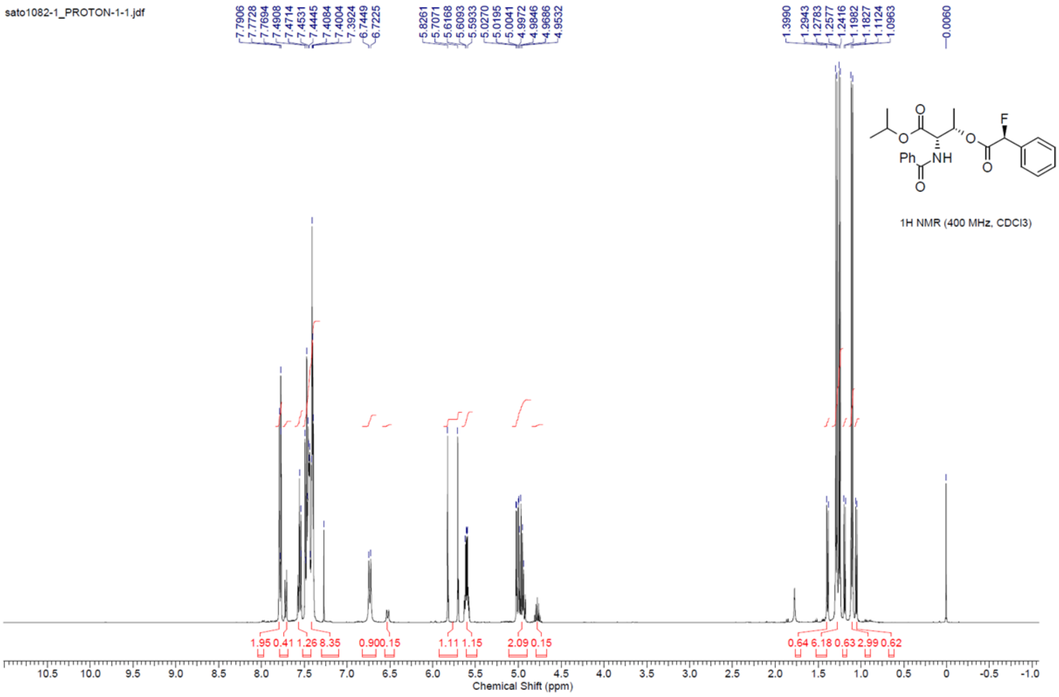

Figure S66. ^{1}H NMR of **10** (400 MHz, $CDCl_3$)

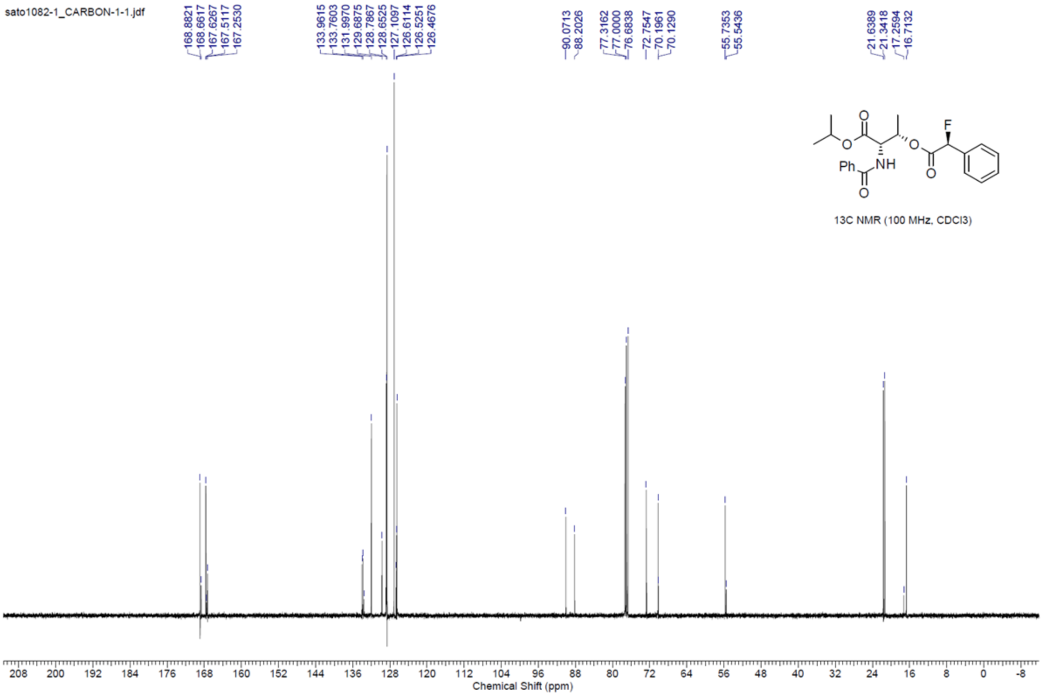

Figure S67. ^{13}C NMR of **10** (100 MHz, $CDCl_3$)

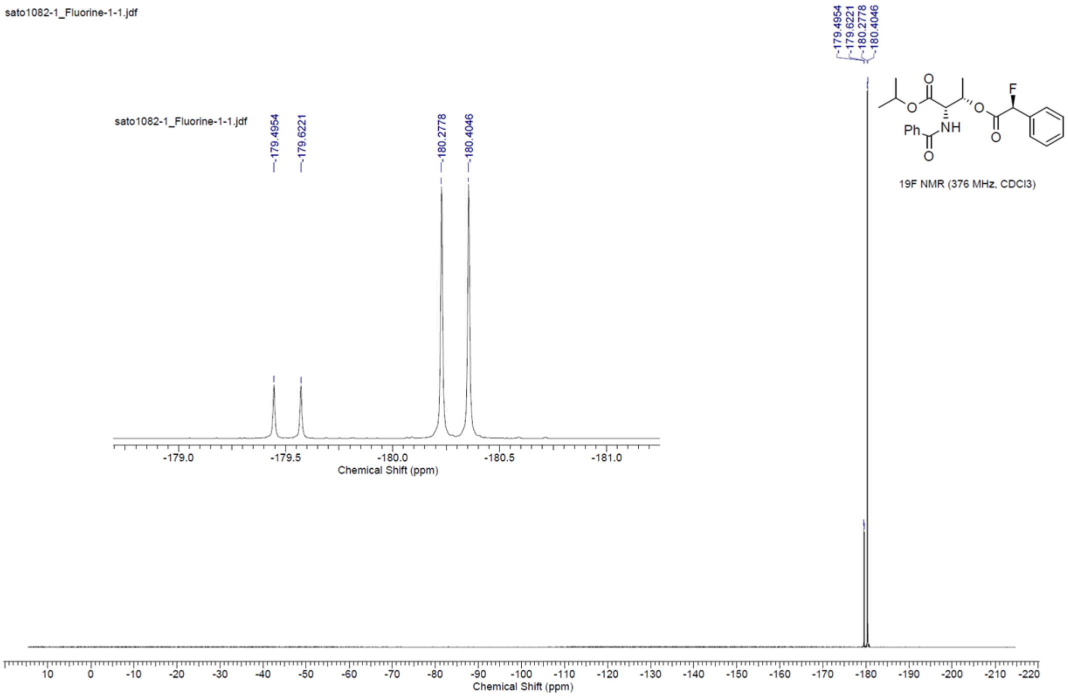

Figure S68. ^{19}F NMR of **10** (376 MHz, $CDCl_3$)
